# Consumer attitudes towards production diseases in intensive production systems

**DOI:** 10.1371/journal.pone.0210432

**Published:** 2019-01-10

**Authors:** Beth Clark, Luca A. Panzone, Gavin B. Stewart, Ilias Kyriazakis, Jarkko K. Niemi, Terhi Latvala, Richard Tranter, Philip Jones, Lynn J. Frewer

**Affiliations:** 1 School of Natural and Environmental Sciences, Newcastle University, Newcastle upon Tyne, United Kingdom; 2 Natural Resources Institute Finland (Luke), Seinäjoki, Finland; 3 School of Agriculture, Policy and Development, University of Reading, Whiteknights, Reading, United Kingdom; Universidade do Porto Instituto de Biologia Molecular e Celular, PORTUGAL

## Abstract

Many members of the public and important stakeholders operating at the upper end of the food chain, may be unfamiliar with how food is produced, including within modern animal production systems. The intensification of production is becoming increasingly common in modern farming. However, intensive systems are particularly susceptible to production diseases, with potentially negative consequences for farm animal welfare (FAW). Previous research has demonstrated that the public are concerned about FAW, yet there has been little research into attitudes towards production diseases, and their approval of interventions to reduce these. This research explores the public’s attitudes towards, and preferences for, FAW interventions in five European countries (Finland, Germany, Poland, Spain and the UK). An online survey was conducted for broilers (n = 789), layers (n = 790) and pigs (n = 751). Data were analysed by means of Kruskal-Wallis ANOVA, exploratory factor analysis and structural equation modelling. The results suggest that the public have concerns regarding intensive production systems, in relation to FAW, naturalness and the use of antibiotics. The most preferred interventions were the most “proactive” interventions, namely improved housing and hygiene measures. The least preferred interventions were medicine-based, which raised humane animal care and food safety concerns amongst respondents. The results highlighted the influence of the identified concerns, perceived risks and benefits on attitudes and subsequent behavioural intention, and the importance of supply chain stakeholders addressing these concerns in the subsequent communications with the public.

## Introduction

Consumers and the public are important stakeholders within the food chain. However, in general, the public knows little about modern animal production systems [1, 2], including intensive systems, which are increasingly used in modern farming. Intensification of livestock farming is, in general, considered as changes in the production system leading to more output being obtained per unit of input used. Intensive production systems represent a change towards more confined animal production systems, whereby animals are kept and raised within fewer production units, with a large increase in the number of animals within these units i.e. an increased stocking density [3]. Intensification can influence factors such as stocking density, group size and management practices applied on the farm, such as nutrition of animals, characteristics of housing and hygienic practices.

In this study, we have considered intensity from the perspective of production diseases in pig and poultry production systems. Production diseases usually originate from a complex interaction of pathogens present on farms, and other factors, which contribute to the disease but do not cause it. Such factors include, *inter alia*, animal genetics and the environment in which the animal is reared (e.g. housing, feed and management practices). Although production diseases occur in all types of production systems, the frequency and scale of disease can increase with the intensity of the production system used [4], with evidence to suggest that larger farms and increased stocking density can lead to reduced welfare, which in turn can negatively impact animal health and increase medication use [5]. Animal health and farm animal welfare (FAW) can be affected adversely, depending on the frequency and scale of disease [6]. Furthermore, this may generate production inefficiencies which affect profitability [7], through reduced growth and feed conversion ratios [6]. In pig production, for instance, herd size is considered as a risk factor for respiratory diseases in pigs [8, 9], although few studies have found no such associations between herd size and respiratory disease (*inter alia* [9]). In pig production, increased stocking density has been associated with the risk of respiratory diseases (e.g. [8, 9]), tail biting (e.g. [10]) and lameness in sows (e.g. [11]). By contrast, some other diseases such as postpartum dysgalactia syndrome in sow herds (e.g. [12]) or piglet mortality (e.g. [13, 14]) are more related to management and housing factors. Intensive production systems may have different approach to animal diseases when compared to extensive systems. On one hand, intensification can increase animals’ stress and the disease pressure they face. On the other hand, intensive production systems are often well-controlled, and measures are taken to control animal health. For instance, biosecurity measures tend to be applied more frequently in large than in small herds (e.g. [15, 16]). There is evidence that the prevalence of different diseases are linked with each other as well as with the level of biosecurity applied on the farm [17].

Growing societal concern about farming practices [18, 19] has been linked to animal and human wellbeing [20, 21], with poor FAW being linked to consumer concerns over product safety [22] and human and animal health [23]. Conversely, higher welfare systems are perceived as guaranteeing safer and healthier products [24, 25]. While some consumers appear appreciative of the quality and safety guaranteed by these systems, a number express concerns in relation to the management practices and associated management and welfare standards applied [20]. For example, studies have identified societal concerns in relation to antibiotic use in animal production [26–28]. Antibiotics usage can be considered as a proxy for the prevalence of production diseases [29], and may be used unnecessarily [30]. Consequently, some consumers are willing to pay premiums for animal-based products to improve FAW, to ensure food safety and reduce the risks they perceive to be associated with intensive animal production, including in relation to animal health, welfare and antibiotic usage [31]. It should be noted that concerns associated with FAW and intensive production do not always correspond to purchase and consumption practices, with sales of higher welfare products lower than reported levels of concern [1]. This indicates a potential discrepancy between an individual’s role as a citizen and as a consumer [20, 32], with individuals expressing different concerns in different contexts. For example, an individual in their role as a consumer may like to purchase and eat meat, and appreciate lower prices, but in their role as a citizen have concerns over how animals involved in food production are raised. This apparent conflict, or citizen-consumer duality, may partly explain the weak link often observed between attitudes and behaviour [33], or the distinctions between the stated attitudes of citizens and observable consumer behaviours.

There is, however, a research gap regarding both citizen and consumer concerns regarding the impacts of animal production diseases, and how this affects consumers’ intention to purchase products from intensive animal production systems. In addition, little is known about consumer attitudes towards interventions to reduce disease prevalence and health impacts, as well as negative effects on FAW [19, 31]. These concerns, linked to public perception of risk, benefit, and ethical concerns, may represent a barrier to the societal acceptance of the increased use of intensive animal production systems. As societal responses may act as a barrier to their long-term use and application [34, 35], understanding concerns, perceptions, attitudes and intentions is therefore essential for providing acceptable animal products for consumers. A greater understanding of citizen concerns will also enable the development of “bespoke” communication strategies tailored to the needs of different groups of consumers [36] and citizens, which, in turn, will act to maintain and increase societal trust in organisations and institutions throughout the supply chain under consideration, and potentially promote societal trust in food chain actors more generally [37].

To develop an understanding of how concerns over production diseases in animal production systems relate to consumer’s behavioural intention, there is a need to understand the psychological processes behind these. Several theoretical frameworks have been used to predict attitudes, including the link between attitude and behaviour, including for example the Theory of Planned Behaviour (TPB) [38]. Numerous studies have utilised measures derived from the TPB to pre-validate variables, and these have been used to successfully identify attitudes as a reliable predictor of behavioural intention [38–40]. Behavioural intention as in relation to food choices [41–43], including animal-friendly foods [44]. In addition, specific factors have been shown may determine attitudes towards an object or behaviour, including perceptions of risks and benefits associated with the topic in question [43], and associated concerns. A number of concerns have been identified in relation to FAW and intensive production systems including those related to humane treatment, naturalness and perceived risks in relation to antibiotic usage, and associated food safety issues [19]. In addition, socio-demographic and socio-economic factors including gender, age, income and education also have influence and have been shown to impact perceptions of risks, benefits and FAW [19, 31, 45, 46].

As the public, including consumers of animal products, are unfamiliar with modern animal production systems and approaches, it is unlikely that they will be familiar with production diseases and the different mitigation strategies proposed. As a consequence, they rely on knowledge from others, with trust in supply chain stakeholders having previously been shown to be an important factor in relation to both FAW and perceptions of risks [37]. Previous research has highlighted that consumers view higher FAW as an additional cue that products are safe and of high quality, with perceived “naturalness” linked to production equating to good welfare [2, 19]. An examination of the associations between these factors, intensive production systems and production diseases would therefore seem important.

Based on the evidence presented above, this study aimed to identify the predictive relationship between perceived risks, perceived benefits, concerns, intervention preferences, familiarity and trust influence and predict, attitudes towards production diseases in intensive production systems, and ultimately, the way these are reflected in consumers’ behavioural intention towards purchasing and consuming products from these systems. Moreover, the relationship between these variables and the acceptance of interventions which aim to control production diseases was examined.

## Methods

### Hypotheses

**Fig 1:**
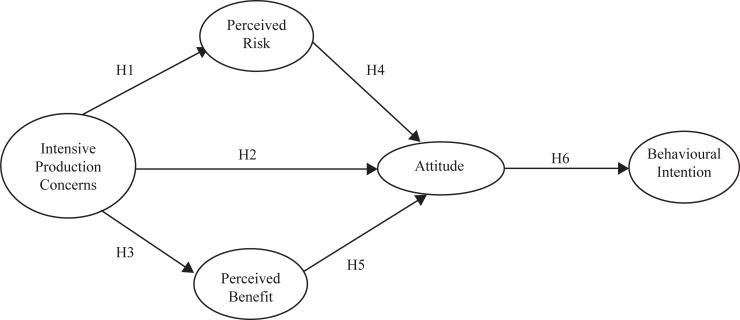
Hypotheses to be tested in the research.

This research tested the following hypotheses, outlining the relationship between concerns, perceived risks and benefits over intensive production, including production diseases, attitudes and behavioural intention (see Fig 1):

H_1_: Greater concern about animal production diseases will increase the perceived risks associated with intensive animal production systems.
H_2_: Greater concern about animal production diseases will lead to less favourable attitudes towards intensive animal production systems.
H_3_ Greater concern about animal production diseases will decrease the perceived benefits associated with intensive animal production systems.
H_4_: Perceived risks associated with intensive animal production systems will negatively influence attitudes towards these systems.
H_5_: Perceived benefits associated with intensive animal production systems will positively influence attitudes towards these systems.
H_6_: A positive attitude towards intensive animal production systems will lead to a positive behavioural intention towards products produced using these systems.

### Survey design

The design of the questionnaire was informed by the existing consumer attitude and behaviour literature, and used adapted versions of previously validated measurement scales (see also [43]). Questions were adapted to make them specific to the context of intensive animal production and production diseases (see [19, 31]). Additional questions were included to address the *a priori* hypotheses (Fig 1), existing research gaps, and to confirm findings [19, 31]. A full overview of the rationale behind the inclusion of each item can be found in Appendix A in S1 File (Table A in S1 File). Semantic differential and Likert scales were used, as these represent the preferred methodological approach to measuring attitudes, perceived risks and perceived benefits (see *inter alia* [47]), and which enables a quantitative analysis to be conducted. These scales are typically constructed from multiple items thought to be reflective of the attitudinal construct of interest. In total, seven scales were developed; 1) *perceived benefit associated with intensive animal production*, *2) perceived risks associated with intensive animal production*, *3) attitudes towards intensive production systems*, *4) trust in food chain stakeholders*, *5) attitudes towards interventions to treat and prevent production diseases*, *6) concerns related to intensive animal production systems and 7) behavioural intention towards products from intensive animal production systems*.

According to industry stakeholders, both reactive and proactive intervention measures exist to combat production diseases within intensive systems [48]. The survey included both types of measures. However, given how unfamiliar the public are with modern farming methods [18, 49], only generic interventions were included to maintain comprehensibility, and a definition of production diseases was provided to participants, which was ‘*Production diseases usually originate from a complex interaction of the viruses and bacteria which are present on farms*, *animal genetics and the environment in which the animal is reared*, *including the characteristics of housing*, *feed and management practices used*. *They differ from epidemic diseases (such as foot and mouth disease or avian influenza) which are caused by new infections from outside the farm*’. Interventions included were consistent with [48]. The descriptions of these interventions were adapted to make them less technical following the pilot study, with the revised phrasing checked by academic and industry experts. A full list of the interventions presented can be found in the section “Intervention preferences”.

Three separate versions of the survey instrument were created, for pigs, layer hens and broiler chickens. The content varied only in relation to the particular interventions associated with each animal type, and the wording of questions made them specific to each. These farm animals were chosen because they are frequently, and increasingly, reared in intensive production systems[50], due to their relatively short reproduction cycles, high feed conversion ratios [51], and grain based diets [52]. This is particularly the case in the developed world where intensive production is prioritised to take advantage of the high feed inputs to optimize feed conversion ratios [50]. Furthermore, the research project this paper stems from was only covering pigs and poultry.

The survey instrument was developed in English and piloted using 45 participants in the UK and, following translation and back translation into and from Finnish, in Finland. Following feedback, changes were made to provide definitions, such as ‘intensive production systems’. The revised survey was then translated into German, Polish and Spanish, and subsequently back-translated to check for translation accuracy and to ensure consistency in the specification of the constructs. A copy of each survey is included in Appendix B in S1 File.

### Sampling and distribution

The survey received ethical approval from Newcastle University’s faculty of Science, Agriculture and Engineering ethics committee before commencing data collection (reference number BH1241898). The survey was distributed in five geographically representative EU countries. A purposeful quota sampling technique was used to obtain samples representing citizens in each of the five countries surveyed (Finland, Germany, Poland, Spain and the UK), based on gender and age quotas to ensure as representative sample as possible in relation to each country’s population. Respondents came from a panel of the social research agency Qualtrics. All questionnaires were distributed online through Qualtrics, between 10^th^ March and 10^th^ April 2017.

### Data analysis

Data analysis was conducted in SPSS version 20 [53] and R [54]. Comparisons of participant responses between countries were then conducted using the Chi square test and Kruskal Wallis test for categorical and Likert scale data respectively. As the Shapiro-Wilk test for normality indicated that none of the variables had a normal distribution, the Kruskal Wallis test was used to check for significant differences between countries regarding household size and number of children. All tests were carried out with the null hypothesis that *there was no significant difference between countries*, at the 5% significance level.

Exploratory factor analysis (EFA) was conducted using SPSS [53], and was used as a data reduction technique to group the different questions asked into a much smaller number of latent variables for subsequent analysis. Principle factor extraction, which makes no distributional assumptions [55], was used due to the non-normal distribution of the variables included. Varimax (orthogonal) and direct-oblimin (oblique) rotations were used in the data analysis in order to compare solutions and examine correlation between the factors identified. In total, 145 variables from the pigs’ survey and 146 variables from the broilers and layers surveys were included in the EFA. i.e. Q4-Q10, Q13, and Q16 (see Appendix B in S1 File) were analysed. Only these questions were included in the EFA due to their theoretically relevant potential explanatory power. All questions were five-point Likert scales, anchored from one to five (strongly disagree to strongly agree) for all questions excluding Q7, where the semantic differential scale was anchored in seven different attitudinal pairs (Unpleasant/ pleasant; bad/good; worthless/ valuable, useless/ useful, unsafe/ safe and unethical/ ethical).

To confirm whether the data were suitable for factor analysis, several checks were performed: inspection of the correlation matrix and the anti-image matrix, the Kaiser-Meyer-Olkin (KMO; [56]) test for sampling adequacy, Bartlett’s test of sphericity and assessment of the determinant. The correlation matrix was inspected to check for multicollinearity and singularity in the data, and to assess whether variables were correlated. The criteria for deciding how many factors to include were based on the Kaiser criterion: all factors with an eigenvalue greater than one were retained [57], together with the use of a scree plot [58]. Only items with a factor loading greater than 0.4 were retained. Although a smaller loading cut-off can be used for larger samples, as for this sample, using a cut-off of 0.4 facilitates interpretation, and enables the inclusion of items which share a greater proportion of variance with the factor [59]. The face validity of the factors was also assessed by evaluating which items had loaded onto each factor. The internal consistency of each returned factor was assessed using Cronbach’s alpha [60]. Non-refined factor scores were created by calculating the average score for each item which loaded onto a factor.

Structural equation modelling (SEM) was conducted using the Lavaan and semTools packages [61, 62], using the maximum likelihood approach, applying a two-step process [63]. Separate models were created for each of the three animal production systems, due to the different intervention questions used. The measurement model describes the relationships between the latent variables included in the analysis, and their observed indicators. In this instance, the structure of the latent variables included in the model was based on the results of the exploratory factor analysis. Identification was ensured by fixing all factor loadings to one, and having at least three indicators on all factors. This was followed by the full structural model, including the causal dependencies between constructs, based on the findings from the two systematic reviews [19, 31], and of the hypotheses generated (Fig 1).

Overall model fit statistics and significance tests were generated for each path within the model. Several indices of model fit were used to examine the model, including the Tucker-Lewis Index (TLI), Comparative Fit Index (CFI), Root Mean Squared Error of Approximation (RMSEA) and Standardised Root Mean Squared Residual (SRMR) and Chi-square. A good fitting model should have RMSEA below 0.05, TFI above 0.9, CFI above 0.9 and SRMR below 0.08 and a non-significant Chi-square test (*p>0*.*05*) [64, 65].

The model was built by adding in the latent variables step-by-step based on the hypotheses (Fig 1), i.e. beginning with the attitude-pro-consumption relationship (attitude-behavioural intention to consume products from intensive animal production systems). All first item loadings on each factor were constrained to one. Modification indices (MI) were checked at each stage and made iteratively to establish whether correlation between residuals was needed, as indicated first by a high MI and second by whether it was theoretically justifiable.

Once the final model was confirmed, multi-group analysis (MSEM) was performed to assess differences across countries. MSEM works by testing for degrees of invariance or measurement equivalency across groups [59]. This establishes the moderating effect of the variables by comparing a series of models, starting from an unrestricted baseline model (model 1) against several increasingly restricted models [66]. Models 2, 3, and 4 have an increasing number of parameters restricted. If there are no differences in the model fit indices for the unrestricted model and the various restricted models, then this indicates that the parameters in measurement and structural components of the theoretical model are equivalent (i.e. invariant) across the sub-groups being compared, and that mean differences can, therefore, be attributed to these variables [59]. Absence of measurement invariance therefore indicates that there is no clear basis for drawing inferences from the model [67]. In the study reported here, the increasingly restricted models are as follows:

- Baseline/ configural invariance (model 1): measurement equivalence model with equal loadings of latent variables on factors similar across sub-groups. Factor items are constrained to be 1 with the other parameters being freely estimated;
- Metric/ weak invariance (model 2): this included model 1 constraints and factor loadings constrained across groups. This enables comparison of the relationships between the latent variables across groups. If this is rejected it means that the factor structure across groups is not the same [59, 68];
- Scaler/ strong invariance (model 3): this included model 2 constraints and the intercepts are constrained across groups. This enables comparisons of the means of the latent variables across groups as it indicates that group differences are not from an unrelated bias [68];
- Strict invariance (model 4): this included model 3 constraints and equal factor means; and
- Fully constrained: this included model 4 constraints and equal residuals (i.e. fully constrained).

## Results

### Sample statistics

#### Descriptive characteristics

Sample characteristics for each of the three surveys are presented in Tables B-D in S1 File (Appendix C in S1 File), with sample sizes of 789, 790 and 751 for broilers, layers and pigs respectively, resulting in 2,330 total responses. They are, in general, representative of national adult populations based on gender and age, albeit with some under-representation of the higher age group, a limitation of the online approach used. The sample populations in Poland and Spain had a lower proportion of respondents in the top income categories, reflective of the lower average incomes in these countries compared with the other study countries. Most respondents indicated that they were omnivores, with few respondents being vegan or vegetarian (all less than 4% and 11% respectively). Germany, Finland and the UK had the highest proportion of vegetarians.

#### General views on intensive systems

Three quarters of respondents in all countries were unfamiliar with modern farming practices (Table E of Appendix C in S1 File), i.e. they had neither lived nor worked on farms, and did not have friends or family involved with farming. Most respondents (range 51.6% - 88.5% answering no to the presented communications channels) had not heard anything about production diseases from any of the information sources mentioned in the survey, including television (57.3%, 51.6% and 58.1% responding no for layers, broilers and pigs), and the internet (64.2%, 62.1% and 66.7% responding no for layers, broilers and pigs), with these being the two sources from which their most relevant information was obtained.

The mean scores in relation to six attitudinal questions averaged three or less (Table 1), corresponding to the negative half of each scale, for all three intensive production systems (pigs, layers, and broilers) considered. Therefore, the majority of respondents viewed these systems unfavourably. Attitudes varied by country (Tables F-H of Appendix C in S1 File). For example, respondents in Germany rated intensive systems more unfavourably than respondents in other countries, especially in relation to them being ‘unpleasant’, ‘bad’ and ‘unethical’, with them having the lowest total scores and the only mean scores averaging less than three.

**Table 1:** Attitudes citizens towards intensive animal production systems for laying hens, broilers and pigs from 5 EU countries (mean rank on a linear scale: 1 to 5 ± SD).

I feel intensive animal production systems are …	Layers (n = 790)	Broilers (n = 789)	Pigs (n = 751)
**Unpleasant (1)/pleasant (5)**	2.22 ± 1.04***	2.09 ± 1.03**	2.18 ± 1.06**
**Bad (1)/good (5)**	2.44 ± 1.05***	2.32 ± 1.05***	2.43 ± 1.04***
**Worthless (1)/valuable (5)**	3.06 ± 1.08***	2.72 ± 1.05***	2.76 ± 1.03***
**Useless (1)/useful (5)**	3.06 ± 1.08***	3.06 ± 1.11***	3.09 ± 1.06***
**Unsafe (1)/safe (5)**	2.64 ± 1.07**	2.63 ± 1.08***	2.65 ± 1.08***
**Unethical (1)/ethical (5)**	2.17 ± 1.04***	2.12 ± 1.06***	2.23 ± 1.10***

***p<0.001

** p<0.01

*p<0.05 indicates significant differences between countries for each attitudinal factor obtained by using a Chi-square test. Some scores have been reversed from their initial presentation in the survey (Appendix A in S1 File) to provide consistency for analysis.

In relation to current purchasing behaviour (Table I of Appendix C in S1 File), most respondents were either unsure whether they purchased foods produced by intensive animal production systems or stated that they did not (strongly disagree/ disagree/ neither agree nor disagree; 67.4% layers, 64.3% broilers, 66.7% pigs). As the majority of such food purchases will have come from intensive systems, this divergence could be due to respondents being uncertain about how the food they purchase has been produced. In terms of food purchase intentions, the majority of respondents indicated that they were unlikely to consider purchasing foods from intensive animal production systems (strongly agree/agree; 70.1% layers, 69.4% broilers, 68.9% pigs). However, they also stated that they did not plan to reduce their consumption of, or avoid purchasing products from these systems. This varied between countries in relation to this (Table I of Appendix C in S1 File).

Despite their negative attitudes towards intensive production systems, resulting in a lack of perceived benefits, just over half of our respondents perceived themselves as being concerned about FAW (strongly agree/agree; 59.7% layers, 57.7% broilers, 57.5% pigs), or intensive production systems (strongly agree/agree; 51.5% layers, 53.4% broilers, 51.5% pigs).

When asked which stakeholders could be trusted to provide them with accurate information on production diseases, *veterinarians*, *animal health authorities*, *animal welfare organisations*, *quality assurance schemes* and *consumer organisations* received the highest mean scores for trust (Table J of Appendix C in S1 File). Stakeholders further down the supply chain, such as animal transporters, received lower mean scores for trust.

In general, there were perceived to be few benefits associated with any of the intensive animal production systems considered (Tables 2–4), with the majority of statements receiving average scores at the lower end of the Likert scale (i.e. less than 3), indicating that the listed factors were not perceived as benefits. In most countries the most likely risks arising from these production systems were perceived to be *increased animal stress* and *an unnatural production method*. The main benefits perceived by participants were perceived to be greater protection for animals (from predators and bad weather), economic benefits (i.e. cheaper and more cost-efficient products) and increased animal product availability for consumers, as indicated by their higher mean scores.

**Table 2:** Risk and benefit perceptions of respondents in the five study countries regarding intensive broiler chicken systems (mean response on a linear scale 1 (strongly disagree) to 5 (strongly agree) ± SD; n = 789).

Perceived as a benefit			Perceived as a risk		
**More cost-efficient production method**	**3.63 ± 1.06**	** **	Increased animal stress	**3.99 ± 1.03**	***
**Cheaper food of animal origin**	**3.58 ± 1.14**	** **	An unnatural production method	**3.82 ± 1.07**	** **
**Increased availability of animal based products**	**3.56 ± 1.08**	** **	Increased incidence of animal diseases	**3.71 ± 1.02**	** **
**Greater protection from predators**	**3.42 ± 1.12**	** **	Reduced nutritional quality of food	**3.64 ± 1.05**	** **
**Greater protection from bad weather**	**3.41 ± 1.13**	***	Compromised FAW monitoring	**3.64 ± 1.04**	** **
**Benefits to agriculture**	**3.16 ± 1.64**	** **	Reduced human food quality	**3.60 ± 1.05**	** **
**More professionally run farms**	**3.04 ± 1.15**	** **	Decreased consumer trust in food	**3.59 ± 1.04**	** **
**Benefits to consumers**	**3.01 ± 1.18**	** **	Reduced human food safety	**3.54 ± 1.06**	** **
**Faster treatment of animal diseases**	**2.96 ± 1.22**	** **	Negative effects on consumer health	**3.54 ± 1.05**	** **
**Benefits to the environment**	**2.94 ± 1.15**	** **	An unsustainable approach to animal production	**3.46 ± 1.09**	** **
**Benefits to you personally**	**2.91 ± 1.26**	** **	Slower treatment of animal diseases	**3.44 ± 1.06**	** **
**Improved human food safety**	**2.89 ± 1.24**	** **	Risks to consumers	**3.38 ± 1.06**	** **
**Benefits to your family**	**2.88 ± 1.23**	** **	Risks to your family	**3.36 ± 1.06**	** **
**Improved FAW monitoring**	**2.84 ± 1.27**	** **	Risks to you personally	**3.34 ± 1.06**	** **
**A more sustainable approach to animal production**	**2.80 ± 1.21**	** **	Risks to the environment	**3.27 ± 1.00**	** **
**Increased consumer trust in food**	**2.78 ± 1.24**	** **	Risks to agriculture	**3.24 ± 1.05**	***
**Improved human food quality**	**2.77 ± 1.28**	** **	Less professionally run farms	**3.05 ± 1.11**	***
**Improved consumer health**	**2.77 ± 1.26**	** **	Less protection from predators	**2.85 ± 1.10**	** **
**Improved nutritional quality of food**	**2.72 ± 1.27**	** **	More expensive food of animal origin	**2.76 ± 1.10**	** **
**Reduced incidence of animal diseases**	**2.71 ± 1.29**	** **	Decreased availability of animal based foods	**2.75 ± 1.10**	** **
**A natural production method**	**2.54 ± 1.32**	** **	Non-cost efficient production method	**2.74 ± 1.12**	** **
**Reduced animal stress**	**2.49 ± 1.32**	** **	Less protection from bad weather	**2.74 ± 1.10**	** **

*** = p<0.002 Bonferroni adjusted p value, for Wilcoxon signed rank tests. Comparisons were made between each statement and the subsequent statement when ranked by mean score.

**Table 3:** Risk and benefit perceptions of respondents in the five study countries regarding intensive layer hen production systems (mean response on a linear scale 1 (strongly disagree) to 5 (strongly agree) ± SD; n = 790).

Perceived as a benefit			Perceived as a risk		
**More cost-efficient production method**	3.59 ± 1.11		Increased animal stress	3.96 ± 1.00	
**Cheaper food of animal origin**	3.58 ± 1.10		An unnatural production method	3.82 ± 1.04	
**Increased availability of animal based products**	3.52 ± 1.05		Increased incidence of animal diseases	3.74 ± 1.03	
**Greater protection from bad weather**	3.43 ± 1.06		Compromised FAW monitoring	3.64 ± 1.05	
**Greater protection from predators**	3.41 ± 1.08	***	Reduced human food quality	3.61 ± 1.07	
**Benefits to agriculture**	3.16 ± 1.12		Reduced nutritional quality of food	3.59 ± 1.07	
**More professionally run farms**	3.07 ± 1.14		Decreased consumer trust in food	3.53 ± 1.05	
**Benefits to consumers**	3.06 ± 1.18		Reduced human food safety	3.52 ± 1.06	
**Faster treatment of animal diseases**	2.97 ± 1.19		An unsustainable approach to animal production	3.50 ± 1.07	
**Benefits to your family**	2.90 ± 1.19		Negative effects on consumer health	3.50 ± 1.05	
**Benefits to the environment**	2.90 ± 1.16		Slower treatment of animal diseases	3.43 ± 1.07	
**Benefits to you personally**	2.89 ± 1.19		Risks to the environment	3.36 ± 1.01	
**Improved FAW monitoring**	2.87 ± 1.24		Risks to consumers	3.33 ± 1.10	
**Improved human food safety**	2.87 ± 1.20		Risks to your family	3.32 ± 1.08	
**A more sustainable approach to animal production**	2.84 ± 1.19		Risks to you personally	3.23 ± 1.09	
**Increased consumer trust in food**	2.81 ± 1.19		Risks to agriculture	3.21 ± 1.07	
**Improved human food quality**	2.76 ± 1.24		Less professionally run farms	3.10 ± 1.10	***
**Improved consumer health**	2.76 ± 1.23		Non-cost efficient production method	2.94 ± 1.10	
**Improved nutritional quality of food**	2.71 ± 1.23		Less protection from predators	2.94 ± 1.10	
**Reduced incidence of animal diseases**	2.69 ± 1.26		Decreased availability of animal based foods	2.82 ± 1.12	
**A natural production method**	2.56 ± 1.33		Less protection from bad weather	2.80 ± 1.11	
**Reduced animal stress**	2.47 ± 1.31		More expensive food of animal origin	2.77 ± 1.13	

*** = p<0.002 Bonferroni adjusted p value, for Wilcoxon signed rank tests. Comparisons were made between each statement and the subsequent statement when ranked by mean score.

**Table 4:** Risk and benefit perceptions of respondents in the five study countries regarding intensive pig production systems (mean response on a linear scale 1 (strongly disagree) to 5 (strongly agree) ± SD; n = 751).

Perceived as a benefit			Perceived as a risk		
**More cost-efficient production method**	3.66 ± 1.07		Increased animal stress	3.88 ± 1.10	
**Cheaper food of animal origin**	3.65 ± 1.05		An unnatural production method	3.78 ± 1.10	***
**Increased availability of animal based products**	3.56 ± 1.03		Increased incidence of animal diseases	3.61 ± 1.08	
**Greater protection from predators**	3.42 ± 1.09		Reduced human food quality	3.56 ± 1.07	
**Greater protection from bad weather**	3.41 ± 1.09	***	Compromised FAW monitoring	3.55 ± 1.06	
**Benefits to agriculture**	3.18 ± 1.14		Decreased consumer trust in food	3.51 ± 1.09	
**More professionally run farms**	3.12 ± 1.17		Reduced nutritional quality of food	3.50 ± 1.07	
**Faster treatment of animal diseases**	3.10 ± 1.20		Reduced human food safety	3.46 ± 1.08	
**Benefits to consumers**	3.07 ± 1.17		Negative effects on consumer health	3.42 ± 1.08	
**Improved FAW monitoring**	3.00 ± 1.25		An unsustainable approach to animal production	3.40 ± 1.05	
**Improved human food safety**	2.99 ± 1.25		Risks to consumers	3.34 ± 1.10	
**Benefits to the environment**	2.96 ± 1.17		Risks to your family	3.33 ± 1.11	
**A more sustainable approach to animal production**	2.89 ± 1.24		Slower treatment of animal diseases	3.31 ± 1.08	
**Benefits to your family**	2.89 ± 1.21		Risks to you personally	3.30 ± 1.01	
**Improved consumer health**	2.87 ± 1.26		Risks to the environment	3.27 ± 1.02	
**Benefits to you personally**	2.87 ± 1.22		Risks to agriculture	3.23 ± 1.04	***
**Improved human food quality**	2.85 ± 1.29		Less professionally run farms	3.04 ± 1.10	
**Increased consumer trust in food**	2.83 ± 1.25		Less protection from predators	2.91 ± 1.09	
**Reduced incidence of animal diseases**	2.80 ± 1.28		Non-cost efficient production method	2.78 ± 1.11	
**Improved nutritional quality of food**	2.80 ± 1.28	***	Less protection from bad weather	2.78 ± 1.10	
**A natural production method**	2.54 ± 1.34		More expensive food of animal origin	2.75 ± 1.12	
**Reduced animal stress**	2.52 ± 1.32		Decreased availability of animal based foods	2.72 ± 1.08	

*** = p<0.002 Bonferroni adjusted p value, for Wilcoxon signed rank tests. Comparisons were made between each statement and the subsequent statement when ranked by mean score.

Although the level of agreement for specific concerns differed between countries (Table K of Appendix C in S1 File), the greatest concerns were common across the five countries. These related to the impacts of intensive production systems on human health, i.e. *food safety and preventative antibiotic use*, particularly *antibiotic residues in food and antibiotic resistance* as a result of the use of antibiotics in animals (all averaging 4 (agree) across general, human and animal focused concerns). The use of medicines to treat animal diseases was associated with less concern than their preventative use. In the cases of layer hens and pigs, respondents from some countries also indicated greater concerns associated with the impacts of animal diseases on animal welfare. In contrast, there were generally lower levels of concern expressed about the use of proactive and preventative health measures, such as *vaccination* and *probiotic use* (averaging 3.5 (neither agree nor disagree/agree or less; Table K of Appendix C in S1 File), with the exclusion of the preventative use of antibiotics.

#### Intervention preferences

Tables 5–7 and Tables L-N in S1 File (Appendix C in S1 File) show the acceptability, to respondents, of different interventions to control production diseases in intensive broiler chicken, layer hen and pig systems. Significant differences existed between countries for most interventions. *Doing nothing* was consistently unacceptable to the majority of respondents, followed by *the preventative use of veterinary drugs*, and the difference between these two interventions was significant across all animal types. The results suggest that respondents generally accept, or are unsure, about the different preventative measures, but are less accepting of “reactive” treatments such as medical intervention, except where these address the main concerns identified in *General views on intensive systems*. The low level of acceptability of the use of veterinary drugs may be related to the perceived risk of antimicrobial resistance resulting from their use, including in relation to human and animal health. Respondents from Spain and the UK were more accepting of the preventative use of veterinary drugs in broiler chickens and pigs compared to respondents in Finland, Germany or Poland. German respondents were less accepting of breeding for genetically disease-resistant pigs compared to respondents in other countries (Table N of Appendix C in S1 File). Respondents in Germany were also less accepting of the use of feed supplements for all animal types compared to respondents in other countries, with Finnish respondents in addition less accepting of their use for layers (Table M of Appendix C in S1 File).

**Table 5:** Mean acceptability scores of interventions to control production diseases in broiler chickens (n = 789).

Intervention	Mean Acceptability Score		
**Enhanced hygiene to prevent diseases**	4.19	±	0.91
**Housing that allows birds greater freedom to move**	4.18	±	0.98
**Providing materials and an environment where birds can perform natural behaviours**	4.15	±	0.92
**Reducing the number of chickens in a given area**	4.12	±	0.90
**Improvements in housing design**	4.10	±	0.89
**Enhanced maintenance of the quality of the bedding**	4.04	±	0.88
**Housing that protects the birds from adverse natural conditions**	4.00	±	0.90
**Enhanced control of air movement in chicken houses**	3.94	±	0.94
**Providing farmers with a price premium that encourages enhanced animal health**	3.94	±	0.93***
**Adjustments to feed composition**	3.63	±	0.97
**Changes in the amount and time of light provision**	3.56	±	1.01
**Adjustments in the quantity of feed available**	3.45	±	1.01
**The use of vaccination**	3.41	±	0.99
**Using antibiotics and medicines to treat sick birds**	3.33	±	1.05***
**Use of feed supplements e.g. probiotics**	3.12	±	1.09***
**The preventive use of veterinary drugs, including antibiotics**	2.88	±	1.17***
**Doing nothing**	2.12	±	1.19

*** = p < 0.003 from pairwise Wilcoxon signed rank tests with -Bonferroni adjusted P value. Comparisons were made between each intervention and the subsequent intervention when ranked by mean acceptability score.

**Table 6:** Mean acceptability scores of interventions to control production diseases in laying hens (n = 790).

Intervention	Mean Acceptability Score		
**Enhanced hygiene and disease prevention measures**	4.18	±	0.86
**Providing materials and an environment where birds can perform natural behaviours**	4.16	±	0.92
**Housing that allows birds greater freedom to move**	4.11	±	0.99
**Reducing the number of chickens in a given area**	4.10	±	0.94
**Improvements in housing design**	4.07	±	0.90
**Enhanced maintenance of the quality of the bedding**	4.06	±	0.88
**Housing that protects the birds from adverse natural conditions**	3.99	±	0.92
**Enhanced control of air movement in chicken houses**	3.92	±	0.92
**Providing farmers with a price premium that encourages enhanced animal health**	3.91	±	0.96***
**Adjustments to feed composition**	3.65	±	0.95
**Changes in the amount and time of light provision**	3.59	±	1.02
**Adjustments in the quantity of feed available**	3.46	±	0.96
**The use of vaccination**	3.45	±	0.98
**Using antibiotics and medicines to treat sick birds**	3.34	±	1.06
**Use of feed supplements e.g. probiotics**	3.19	±	1.07
**The preventive use of veterinary drugs, including antibiotics**	3.17	±	1.21***
**Doing nothing**	2.12	±	1.21

*** = p < 0.003 from pairwise Wilcoxon signed rank tests with Bonferroni adjusted p value Comparisons were made between each intervention and the subsequent intervention when ranked by mean acceptability score.

**Table 7:** Mean acceptability scores of interventions to control production diseases in pigs (n = 751).

Intervention	Mean Acceptability Score		
**Providing enrichment materials so pigs can perform natural behaviours**	4.19	±	0.88
**Improvements in pigs’ diet composition**	4.18	±	0.88
**Efficient monitoring of pigs and pig housing conditions**	4.17	±	0.87
**Enhanced hygiene and disease prevention measures**	4.16	±	0.87
**Enhanced control of air movement in pig houses**	4.16	±	0.87
**Improvements in housing design**	4.13	±	0.89
**Reducing the number of pigs in a given area**	4.13	±	0.91
**Providing farmers with a price premium that encourages enhanced animal health**	4.09	±	0.92
**Housing that protects the pigs from adverse natural conditions**	3.96	±	0.94
**Adjustments in the quantity of pig feed available**	3.85	±	0.91***
**The use of vaccination**	3.52	±	0.97
**Using medicines and antibiotics to treat sick pigs**	3.37	±	1.02
**Breeding for genetically tougher or more resilient pigs**	3.26	±	1.14
**Use of feed supplements e.g. probiotics**	3.18	±	1.10***
**The preventive use of veterinary drugs, including antibiotics**	2.75	±	1.18***
**Doing nothing**	2.36	±	1.30

*** = p < 0.003 from pairwise Wilcoxon signed rank tests with Bonferroni adjusted p value. Comparisons were made between each intervention and the subsequent intervention when ranked by mean acceptability score.

Despite national differences, there were similarities in terms of the interventions that were, and were not, perceived as being most acceptable. The most preferred interventions were preventive measures that involved *changes to housing design*, *enhanced hygiene*, *reducing stocking densities* and *provision of enrichment materials*. These interventions were perceived to be more natural and less invasive compared to the other proposed interventions.

In relation to the rationale behind respondents’ level of acceptability of the different interventions, significant differences were observed across the five countries for each of the interventions presented. There were some similarities across animal species and countries, with medicine-based interventions being least preferred due to food safety concerns. The more acceptable housing and hygiene-based interventions were perceived as being more humane (Appendix D in S1 File).

Of interest is the relatively low level of acceptability of interventions that would be key in efforts to reduce the use of pharmaceuticals, particularly antimicrobials, in all countries. This suggests potential misunderstanding amongst the general public of the true nature of interventions to control production diseases involving the genetic improvement of animals, use of vaccination and use of feed supplements, including probiotics.

### Identification of driving factors for behavioural intention

Full results of the exploratory factor analysis (EFA), including the items included within each factor and the mean factor scores can be found in Tables O-T in S1 File (Appendix E in S1 File). The exploratory factor analysis for broilers, layers and pigs had KMO values of 0.945, 0.947 and 0.946 respectively, which are greater than the value of 0.5 which Kaiser [56] describes as acceptable for factor analysis to proceed, with Bartlett’s test of sphericity producing significant results for all scales indicating that factor analysis was appropriate. Comparison of the unrotated, orthogonal and oblique rotations for all scales led to the oblique factor rotation being chosen for inclusion in the analysis, due to the correlations that were present between most factors identified within each scale. 16, 17 and 15 factors were retained for broilers, layers and pigs which explained 69.41%, 70.11% and 68.93% of the data respectively. Scale reliability for all returned factors (latent variables) was good, being above the generally accepted value of 0.7 for all factors, with the exception of factor 16 for broilers and factor 15 for layers, whereby Cronbach’s alpha scores of 0.62 and 0.67 (media) for the layers, broilers and pigs surveys respectively. However, it has been noted that, for psychological variables, values below 0.7 can be expected [67], with reliability score values from 0.5 to 0.6 suggested as the minimum acceptable level in these instances ([69], as cited in [70]). Therefore, all the factors seem to be reliable.

The results of the EFA reflect those of the descriptive statistics, in relation to the types of concerns, risks and benefits held and the type of interventions preferred, with interventions being grouped into factors based on similar concerns e.g. those to do with medicine. EFA results led to the choice of five factors (latent variables) for inclusion in the SEM. These were chosen in relation to their representativeness of the variables included in Fig 1, with the concern factor containing a number of items in relation to production diseases.

### Consequences of concerns and attitude on purchase intentions

The five variables included in each of the models are summarised in Table 8 along with a brief description of the construct. The same model (Fig 1) was tested for each of the animal types studied. Evaluation of the five-factor model for each animal type is summarised in Table 9 and indicates average to good model fit. Table 10 summarises the direct effects for each animal type.

**Table 8:** The five variables included in the structural equation modelling analysis.

Construct	Description
**Pro-consumption**	Pro-consumption behavioural intention of products from intensive animal production systems
**Attitude**	Attitude towards intensive animal production systems
**Perceived benefit**	Perceived benefits of intensive animal production systems
**Perceived risk**	Perceived risks of intensive animal production systems
**Concern**	Perceived concern over intensive animal production systems, including in relation to production diseases

**Table 9:** Goodness of fit statistics for the structural equation modelling for each animal type.

	Layers (n = 790)	Broilers (n = 789)	Pigs (n = 751)
**X^2^**	**X**^2^(479) = 1703.700, p = 0.000	**X**^2^ (335) = 1122.421, p = 0.000	**X**^2^ (721) = 2934.676, p = 0.000
**TLI**	0.93	0.941	0.904
**CFI**	0.937	0.948	0.911
**SRMR**	0.056	0.072	0.077
RMSEA*	0.057 (0.054, 0.060)	0.055 (0.051, 0.058)	0.063 (0.061, 0.065)

*Results are presented with 95% confidence intervals. TLI = Tucker-Lewis Index, CFI = Comparative fit index, SRMR = Standardised root mean squared residual, RMSEA = Root mean squared error of approximation

**Table 10:** Direct effects of the SEM model presented in Fig 1, for broilers, layer hens and pigs.

	Layers (n = 790)	Broilers (n = 789)	Pigs (n = 751)
**Perceived benefit**			
Concern	-0.014	0.002	-0.051*
**Perceived risk**			
Concern	0.439***	0.552***	0.526
**Attitude**			
Perceived risk	-0.308***	-0.291***	-0.277***
Perceived benefit	0.468***	0.303***	0.336***
Concern	-0.294***	-0.177***	-0.168***
**Intention**			
Attitude	0.727***	1.008***	0.819***
**R^2^ attitude**	0.438	0.369	0.349
**R^2^ intention**	0.291	0.417	0.325

* p<0.05

***p<0.001

All relationships were significant except between concern and perceived benefit for layer hens and broilers, and for between concern and perceived risks for pigs. Although differences existed between animal types, several similar trends were identified. Attitude had a large positive and significant effect on respondents’ behavioural intention to consume products derived from all animal types, indicating that a positive attitude towards intensive animal production systems leads to a greater intention to purchase products derived from them. Perceived risk and general concern had a consistently negative effect on attitude across animal types, being largest for broiler chickens, indicating that general concerns and perceived risks have a greater effect on attitudes to purchase chicken meat. The concern-risk, and the concern-benefit relationships were a similar size across all three animal types, indicating a small effect of concern on perceived benefits, and a moderate effect of concern on perceived risks. To summarise, hypotheses H1 to H6 are supported by the model for pigs and broilers, although H3 is not significant for broilers; H3 is also not supported for layer hens, with concern having a slight positive effect on perceived benefit, although this was non-significant.

Multi-group analysis by MSEM was conducted to establish the potential moderating effects of country and Tables U-W in S1 File (Appendix F in S1 File) summarises the results of these tests across the four models outlined in the ‘Data Analysis’ section above for each of the three animal types. Results of the MSEM show significant differences between the baseline model and the series of increasingly restricted models, with measurement invariance not being demonstrated for models 2, 3 and 4. This means that the structure of the latent variables cannot be said to be the same across countries and subsequently, models cannot be compared across countries. This is unsurprising as cultural differences between each of the five countries could lead to different prioritisation and consideration of concerns, risks and benefits.

## Discussion

### Public concerns

Three separate surveys were used to assess citizen attitudes towards, and concerns about, intensive broiler chicken, layer hen and pig production systems, including perceptions of risks and benefits and concerns about production diseases. The findings demonstrate that the public perceive such intensive systems as having benefits, primarily “anthropocentric” benefits (i.e. benefits to humans), such as reducing cost and increasing availability of animal-based products. They did however, express some concerns about these intensive production systems. Indeed, the majority of respondents had a tendency to view these production systems unfavourably on a number of dimensions, for example viewing them as unpleasant, bad, worthless, useless, unsafe, and unethical rather than the opposite. This supports the findings of the most recent Eurobarometer survey [18], where 82% of respondents believed that farm animals should be better protected than they are currently. Given that a key part of the sustainability of a system is its’ level of public acceptance [71], actions to increase acceptance of intensive systems will be required to strengthen this dimension of their sustainability.

A number of elevated risks were identified in relation to intensive production systems, with the most agreement surrounding issues of animal stress, unnaturalness of the production method, and livestock disease. Elevated perceptions of risk were also associated with prophylactic antibiotic usage, antibiotic resistance, antibiotic residues and food safety. When asked about their concerns in relation to animal health, similar concerns arose in relation to production diseases in general and whether minimum welfare standards were being achieved in each of the three production systems. These findings reflect consumer/ public concerns revealed in a recent review [19], and the perceived risks identified in the survey. They also indicate that the public cannot dissociate the risks to food safety and human health from animal health and wellbeing in animal production systems, with production diseases in particular being perceived to have negative consequences for food safety and quality.

Despite their concerns over FAW and intensive animal production, the public may have little or no understanding of modern farming practices [24], and are unfamiliar with the norms associated with FAW [33, 72]. The results of our survey confirm the lack of public knowledge surrounding production diseases, with respondents having a general unfamiliarity with farming. This is unsurprising given that there is little communication about production diseases, and interventions designed to mitigate these, made available to the public by most mainstream media, outside of reporting of economically damaging notifiable infectious diseases, such as foot and mouth disease [73] or avian influenza [74]. In light of this, attitudes towards and perceptions of production diseases may be informed by knowledge and concerns about these widely reported epidemic disease outbreaks. Care should, therefore, be taken when communicating information about production diseases to clearly differentiate them from epidemic disease outbreaks, in order to enable the public to distinguish between the two. Moreover, our results suggest that most consumers do not associate the food they consume with intensive production systems, suggesting that public unfamiliarity with current production systems leads them to believe that intensive production systems do not constitute the majority of European production capacity.

The use of antibiotics, and the related issue of antimicrobial resistance (AMR), were repeatedly identified as concerns both in relation to human and animal health, and intensive animal production systems in general. Antibiotics, and the use of other veterinary medicines, have obvious benefits to animal health [4]. However, the prophylactic use of antibiotics, including their use as growth promoters, was identified as a concern, including in relation to AMR, despite it being emphasised in the survey that this is banned in the EU. AMR represents a transboundary risk insomuch that it does not respect, and can easily cross, international borders [75]. Given the prevalence of AMR globally, including in relation to key types of antibiotics [76], it is imperative to address the concerns that the public, and consumers may hold, in both communication and policy strategies.

Global considerations are particularly important when considering AMR, with the illegal usage of antimicrobials occurring within both developed and developing countries e.g. Italy and China [77, 78]. Three of the top five countries in terms of use of antimicrobials in animal production systems were low or middle-income countries, indicating a shift towards more intensive production systems within these regions [79]. International standards, and enforcement of responsible practice is therefore required to ensure public trust and perceived safety, of animal products especially from regions where antimicrobial usage is less regulated. In addition, as the areas where intensive animal production is increasing are also areas where little is known about related consumer perceptions, it is important to explore public perceptions in these countries.

### Interventions preferred by the public to counter diseases

The most preferred interventions identified were proactive disease prevention measures, as opposed to more reactive disease treatments. These prevention measures included changes to housing design, increasing the floor space available for animals and enhanced hygiene. However, several types of prevention measures were not favoured by the citizens included in the survey, i.e. those that depended on use of pharmaceutical products, i.e. vaccination or prophylactic use of medicines. The use of probiotics and changes to feed composition were also not preferred in poultry, which could be due to a lack of understanding or clarity as to exactly what these interventions involve. For example, changes to feed composition could be construed as feed denial, rather than changing nutrient balance, and probiotics could be construed as a pharmaceutical intervention, or the feeding of products which are not natural to the animal’s diet. This highlights a need to provide sufficient information when explaining to the public what these different management strategies involve. When ranked by mean scores according to their acceptability, respondents to the broiler and laying hens, the results suggested that the participants ranked these measures almost identically. Measures for pigs had a slightly different acceptability ranking. In particular, feed-related measures were ranked substantially more favourably in pigs than in poultry, perhaps due to perceptions that pigs, being natural scavengers, can thrive on a much wider range of foodstuffs. It was interesting to note that there was relatively little difference in preference for the type of intervention measures preferred across animal types.

The least preferred interventions related to factors identified by respondents as concerns e.g. antibiotic usage, vaccination and feed supplementation in poultry. Although the latter two were not among the highest ranked concerns, respondents were more accepting of more natural, or less invasive, strategies. They were preferred in relation to both food safety and humane treatment reasons, again emphasising that the public cannot separate animal wellbeing from human food safety. This further emphasises the need for effective communication and assurance regarding methods to ensure the safety of the animal products.

The results of the survey highlight several concerns held by the public in relation to the interventions used in intensive animal production systems, more specifically in relation to more reactive interventions that were rated as unacceptable to respondents. This ties in with the results of review of public attitudes towards animal welfare in the context of intensive animal production systems [19], which suggested that intensive animal production systems were perceived to breach the concepts of good welfare, humane treatment and naturalness. In addition, the interventions that were least preferred were those that could also have implications for public health, for example as a consequence of compromised food safety or increasing the risk of AMR. This relates back to the priority concerns of consumers when making food choices, and the dual perspectives of FAW, in that the benefits of these systems can be viewed from both anthropocentric and zoocentric perspectives. This has implications for communication of the benefits needing to be tailored to consumers to accommodate these perspectives.

The concerns in relation to prophylactic antibiotic use identified in [19], were supported by findings of the survey, and again showed that public concerns with FAW were related to concerns about human health. It may be that respondent’s use “intensive production” as a heuristic, or decision rule, to indicate that any further information regarding these production systems and interventions will align with this initial decision and processed accordingly [80]. Intensive systems, as a term, may be interpreted and used as a cue for a number of perceived negative consequences. It is important to acknowledge this within policy design and communication strategies, as failure to do so could result in decreased stakeholder trust and subsequent increase in concern and perceived risks.

The nature of the least acceptable interventions is consistent with previous research, which has identified that the public are concerned about eating potentially contaminated meat, particularly after disease epidemics. For example, Breakwell [73] conducted focus groups after the UK foot and mouth disease crisis of 2001. The results of this research suggested that there was no indication that consumers would not consume meat from vaccinated animals, when vaccination was presented as an alternative to the culling strategy employed. However, Scudamore [81] reports that the consumers had some reservations about eating meat from animals vaccinated against foot and mouth disease, and Zingg and Siegrist [41] also reported a reluctance on the part of consumers to eat meat from animals vaccinated against an epidemic disease. Given the lack of public acceptability surrounding such interventions, their use should be justified clearly when being discussed in communication with the public, or only be used when the other proactive measures are not effective enough to ensure animal health and wellbeing, or when the alternatives are not economically viable.

The intervention preferences identified in the survey align with those expressed in a consultation of pig and poultry industry stakeholders [48], in which s stakeholder preference for intervention measures such as biosecurity, improved ventilation, health monitoring and litter quality was identified. The combined results suggest that communication needs to focus on animal physical and psychological wellbeing. This is obviously the case for animal production systems currently, but clearly there is a need to emphasise this to external stakeholders, such as the public, who are not familiar with the current practices and standards used.

Identifying and understanding public attitudes and concerns in relation to intensive farming practices, will ensure that the processes and interventions align with the values, needs and expectations of society [82]. Communicating the proactive management measures to mitigate production diseases is, therefore, important to ensure that these, and the associated risk management procedures, align with societal preferences, and highlights the benefits of ongoing societal discussions in building consumer trust through transparency [83]. Best practice examples of this include activities within the UK poultry sector to reduce the amount of antimicrobials used within production systems [84]. The proactive interventions preferred also emphasise the key aspects of good FAW as identified by Clark *et al* [19], especially naturalness, and the importance of housing-based interventions. Repetition of this theme across the perceived risks, lack of benefits, identified concerns and preferred interventions, highlights the need to ensure that production systems are perceived to address these concerns.

### Trust

Despite the EU having some of the most stringent animal health and welfare regulations globally, most groups of stakeholders were not trusted by respondents to provide reliable information about these systems, with respondents distrusting most supply chain stakeholders. Organizations *independent* of the animal production process were viewed as more trustworthy by respondents, but were still not trusted to provide information. These organisations included animal health, welfare and consumer organisations, quality assurance schemes, and governing bodies. This suggests that it is important to have independent, third-party stakeholders and systems in place to provide assurance to both consumers and the wider public that the food they consume is being produced to requisite standards. Collaborative messages provided across stakeholder groups with different perceived vested interests may also promote trust. This is of particular importance in the current case as welfare standards cannot be identified from the product itself, i.e. FAW is a credence attribute [85]. Transparency, and communication, of how this independent assurance is guaranteed, and the subsequent results of the evaluations of independence, is also important for maintaining public trust [83].

These findings also have implications for market-based solutions for ensuring the standards of higher welfare products, such as certification schemes and associated labelling. Although these offer guarantees, Lassoused and Hobbs [86] found that brands were not enough to enhance consumer confidence in food safety alone. The findings from the consumer survey support this, with the highest level of trust in stakeholders in relation to information provision being those external to the food chain. External accreditation and assurance therefore potentially act as guarantees, and a means of traceability and food standards for consumers, giving them confidence in the product choices which they are making. This implies that co-ordinated communications from the whole food system are needed to improve public confidence in food supply.

The findings reflect several of the factors known to mediate perceived risks, namely in relation to naturalness, the perceived level of control individuals have over the concerns raised, the unfamiliarity with the topic and the lack of consumer trust in the stakeholders involved. In addition, the only experience respondents are likely to have had with animal disease is related disease epidemics reported by the media, rather than production diseases, which may increase their perceived risks and concerns. Actions to ensure that all supply chain actors are perceived as trustworthy is therefore vital in ensuring that perceived risks and concerns are reduced. Mechanisms for improving trust in the food industry include considering what is concerning the public, and adopting intervention measures that are perceived to be more natural, and being more proactive in communications. Thus these mediating factors should be considered by stakeholders, including policy makers, with policy interventions designed either to enhance use of interventions most trusted by the public as a means of increasing public trust in the whole food system, or developing communication programmes to increase trust in interventions not currently preferred, or both [36, 86]. This should also enable any public scepticism to be addressed [87].

### Limitations

A limitation of the online survey methodology used in our study is that it may disadvantage some groups from participating, such as those in older age categories–these were in fact underrepresented across each of the study countries. In some countries, the proportion of respondents with a university degree is fairly high and there is also some variation in terms of other socio-demographic parameters. Although underrepresented groups could have been targeted by other means, such as by face-to-face, or by telephone interviewing, this was not feasible in this case due to resources limitations and differences which may arise because of different methodologies being applied. Future research could also look to explore attitudes towards other intensively farmed animals, such as dairy cows, to provide a comparison to the animals investigated within this study.

As with all SEM analyses, there could exist multiple equivalent model solutions and this could be the case here. In other disciplines, such as ecology, model averaging is used to reduce the uncertainty surrounding model selection, and this is something that could be assessed through additional analysis to this data set analysed here. In addition, measurement invariance was not achieved in the MSEM across countries. Further analysis could be conducted to explore these differences further. Despite this limitation, the model constructed was based on theoretical and evidence-based decisions, and has reasonable goodness of fit statistics. In addition, the findings from the model were supported by the descriptive analysis of the results have been interpreted more generally, to enable any uncertainty to be taken into consideration.

## Conclusion

This research sought to explore the attitudes of the public in five EU countries towards intensive animal production systems, production diseases and some associated mitigation strategies. The results indicated that, while the public is largely unfamiliar with modern animal production, they none-the-less perceive intensive production systems negatively. Various perceived risks and benefits associated with intensive animal production were identified as being salient to attitude formation, and these are translated into concerns, primarily in relation to antibiotic and medicine usage, together with the implications for animal and human health and food safety.

More natural and proactive interventions to control production diseases were preferred which primarily involved changes to housing, housing-related management and hygiene practices, with more reactive and medicine based interventions being least preferred, as they linked into general health and welfare concerns associated with intensive livestock production. Concerns, perceived risks and benefits were identified, and shown to be influential regarding attitude formation, on attitudes and subsequent consumer behavioural intentions. Given this lack of public awareness, coupled with elevated concern, stakeholders need to be more proactive in terms of the information they are providing to the public.

## Supporting information

S1 File (DOCX)

## References

[1] European Commission. Attitudes of EU citizens towards animal welfare. 2007.

[2] Kjærnes U, Lavik R. Farm animal welfare and food consumption practices: Results from surveys in seven countries. Cardiff: School of City and Regional Planning, Cardiff Univeristy, 2007.

[3] Fraser D. Animal welfare and the intensification of animal production. An alternative interpretation. 2005.

[4] Perry BD, Grace D, Sones K. Current drivers and future directions of global livestock disease dynamics. Proceedings of the National Academy of Sciences. 2013;110(52):20871–7. 10.1073/pnas.1012953108 PMC3876220 21576468

[5] Dawkins MS. Animal welfare and efficient farming: is conflict inevitable? Animal Production Science. 2017;57(2):201–8.

[6] Bengtsson B, Greko C. Antibiotic resistance—consequences for animal health, welfare, and food production. Upsala Journal of Medical Sciences. 2014;119(2):96–102. 10.3109/03009734.2014.901445 24678738 PMC4034566

[7] Niemi JK, Jones P, Tranter R, Heinola K. Quantification of costs of existing pathologies for pigs and poultry systems. 2015.

[8] Stärk KD. Epidemiological investigation of the influence of environmental risk factors on respiratory diseases in swine—a literature review. The Veterinary Journal. 2000;159(1):37–56. 10.1053/tvjl.1999.0421 10640410

[9] Maes D, Segales J, Meyns T, Sibila M, Pieters M, Haesebrouck F. Control of Mycoplasma hyopneumoniae infections in pigs. Veterinary microbiology. 2008;126(4):297–309. 10.1016/j.vetmic.2007.09.008 17964089 PMC7130725

[10] D’Eath R, Arnott G, Turner S, Jensen T, Lahrmann H, Busch M, et al Injurious tail biting in pigs: how can it be controlled in existing systems without tail docking? Animal. 2014;8(9):1479–97. 10.1017/S1751731114001359 25130712

[11] Willgert KJ, Brewster V, Wright AJ, Nevel A. Risk factors of lameness in sows in England. Preventive veterinary medicine. 2014;113(2):268–72. 10.1016/j.prevetmed.2013.10.004 24331733

[12] Papadopoulos GA, Vanderhaeghe C, Janssens GP, Dewulf J, Maes DG. Risk factors associated with postpartum dysgalactia syndrome in sows. The Veterinary Journal. 2010;184(2):167–71. 10.1016/j.tvjl.2009.01.010 19230728

[13] KilBride A, Mendl M, Statham P, Held S, Harris M, Cooper S, et al A cohort study of preweaning piglet mortality and farrowing accommodation on 112 commercial pig farms in England. Preventive veterinary medicine. 2012;104(3–4):281–91. 10.1016/j.prevetmed.2011.11.011 22197175

[14] Pandolfi F, Edwards S, Robert F, Kyriazakis I. Risk factors associated with the different categories of piglet perinatal mortality in French farms. Preventive veterinary medicine. 2017;137:1–12. 10.1016/j.prevetmed.2016.12.005 28107875

[15] Ribbens S, Dewulf J, Koenen F, Mintiens K, De Sadeleer L, de Kruif A, et al A survey on biosecurity and management practices in Belgian pig herds. Preventive veterinary medicine. 2008;83(3–4):228–41. 10.1016/j.prevetmed.2007.07.009 17850906

[16] Niemi JK, Sahlström L, Kyyrö J, Lyytikäinen T, Sinisalo A. Farm characteristics and perceptions regarding costs contribute to the adoption of biosecurity in Finnish pig and cattle farms. Review of Agricultural, Food and Environmental Studies. 2016;97(4):215–23.

[17] Pandolfi F, Edwards SA, Maes D, Kyriazakis I. connecting Different Data sources to assess the interconnections between Biosecurity, health, Welfare, and Performance in commercial Pig Farms in great Britain. Frontiers in veterinary science. 2018;5:41 10.3389/fvets.2018.00041 29560358 PMC5845643

[18] European Commission. Attitudes of Europeans towards animal welfare European Union: Director Generate for Health and Social; 2016. Special Eurobarometer 442:[Available from: http://ec.europa.eu/COMMFrontOffice/publicopinion/index.cfm/Survey/getSurveyDetail/instruments/SPECIAL/surveyKy/2096.

[19] Clark B, Stewart GB, Panzone LA, Kyriazakis I, Frewer LJ. A Systematic Review of Public Attitudes, Perceptions and Behaviours Towards Production Diseases Associated with Farm Animal Welfare. Journal of Agricultural and Environmental Ethics. 2016:1–24. 10.1007/s10806-016-9615-x

[20] Boogaard BK, Bock BB, Oosting SJ, Wiskerke JSC, van der Zijpp AJ. Social Acceptance of Dairy Farming: The Ambivalence Between the Two Faces of Modernity. Journal of Agricultural and Environmental Ethics. 2011;24(3):259–82.

[21] Spooner JM, Schuppli CA, Fraser D. Attitudes of Canadian citizens toward farm animal welfare: A qualitative study. Livestock Science. 2014;163(1):150–8.

[22] Verbeke W, Pérez-Cueto FJA, Barcellos MDd, Krystallis A, Grunert KG. European citizen and consumer attitudes and preferences regarding beef and pork. Meat Science. 2010;84(2):284–92. 10.1016/j.meatsci.2009.05.001 20374787

[23] Bennett RM, Butterworth A, Jones PJ, Kehlbacher A, Tranter RB. Valuation of animal welfare benefits: A report to DEFRA. University of Reading, 2012.

[24] Harper G, Henson S. Consumer concerns about animal welfare and the impact on food choice. Reading: Centre for Food Economics Research, The University of Reading, 2001.

[25] Lusk JL, Norwood FB, Prickett RW. Consumer preferences for farm animal welfare: Results of a nationwide telephone survey. Oklahoma State University, Department of Agricultural Economics 2007.

[26] Bernard JC, Bernard DJ. What is it about organic milk? An experimental analysis. American Journal of Agricultural Economics. 2009;91(3):826–36.

[27] Vanhonacker F, van Poucke E, Tuyttens F, Verbeke W. Citizens' Views on Farm Animal Welfare and Related Information Provision: Exploratory Insights from Flanders, Belgium. Journal of Agricultural and Environmental Ethics. 2010;23(6):551–69.

[28] Miele M, Veissier I, Evans A, Botreau R. Animal welfare: establishing a dialogue between science and society. Animal Welfare. 2011;20(1):103.

[29] Hughes P, Heritage J. Antibiotics as animal growth promoters. 2002.

[30] Hudson JA, Frewer LJ, Jones G, Brereton PA, Whittingham MJ, Stewart G. The agri-food chain and antimicrobial resistance: A review. Trends in Food Science & Technology. 2017;69(Part A):131–47. 10.1016/j.tifs.2017.09.007.

[31] Clark B, Stewart GB, Panzone LA, Kyriazakis I, Frewer LJ. Citizens, consumers and farm animal welfare: A meta-analysis of willingness-to-pay studies. Food Policy. 2017;68:112–27. 10.1016/j.foodpol.2017.01.006.

[32] Grunert KG. Future trends and consumer lifestyles with regard to meat consumption. Meat science. 2006;74(1):149–60. 10.1016/j.meatsci.2006.04.016 22062724

[33] Te Velde H, Aarts N, Van Woerkum C. Dealing with ambivalence: Farmers' and consumers' perceptions of animal welfare in livestock breeding. Journal of Agricultural and Environmental Ethics. 2002;15(2):203–19.

[34] Frewer L. The public and effective risk communication. Toxicology letters. 2004;149(1–3):391–7. 10.1016/j.toxlet.2003.12.049 15093286

[35] Fischer AR, Frewer LJ. Consumer familiarity with foods and the perception of risks and benefits. Food Quality and Preference. 2009;20(8):576–85.

[36] de Jonge J, Van Trijp JCM, van der Lans IA, Renes RJ, Frewer LJ. How trust in institutions and organizations builds general consumer confidence in the safety of food: A decomposition of effects. Appetite. 2008;51(2):311–7. 10.1016/j.appet.2008.03.008 18450326

[37] Poortinga W, Pidgeon NF. Trust in risk regulation: Cause or consequence of the acceptability of GM food? Risk analysis. 2005;25(1):199–209. 10.1111/j.0272-4332.2005.00579.x 15787769

[38] Ajzen I. From intentions to actions: A theory of planned behavior Action control: Springer; 1985 p. 11–39.

[39] Ajzen I. The theory of planned behavior. Organizational behavior and human decision processes. 1991;50(2):179–211.

[40] Ajzen I. The theory of planned behaviour: reactions and reflections. Taylor & Francis; 2011. 10.1080/08870446.2011.613995 21929476

[41] Zingg A, Siegrist M. People’s willingness to eat meat from animals vaccinated against epidemics. Food Policy. 2012;37(3):226–31. 10.1016/j.foodpol.2012.02.001.

[42] Fischer ARH, Frewer LJ. Consumer familiarity with foods and the perception of risks and benefits. Food Quality and Preference. 2009;20(8):576–85. 10.1016/j.foodqual.2009.06.008.

[43] Poínhos R, van der Lans IA, Rankin A, Fischer ARH, Bunting B, Kuznesof S, et al Psychological determinants of consumer acceptance of personalised nutrition in 9 European countries. PloS one. 2014;9(10):e110614 10.1371/journal.pone.0110614 25334009 PMC4204923

[44] Nocella G, Boecker A, Hubbard L, Scarpa R. Eliciting Consumer Preferences for Certified Animal-Friendly Foods: Can Elements of the Theory of Planned Behavior Improve Choice Experiment Analysis? Psychology & Marketing. 2012;29(11):850–68. 10.1002/mar.20569 WOS:000309740600004.

[45] Kirk SFL, Greenwood D, Cade JE, Pearman AD. Public perception of a range of potential food risks in the United Kingdom. Appetite. 2002;38(3):189–97. 10.1006/appe.2001.0478 12071684

[46] Food and Acriculture Organization of the United Nations, Organization WH. The application of risk communication to food standards and safety matters. Rome: Food and Agriculture Organization, 1998.

[47] van Dijk H, Fischer AR, Frewer LJ. Consumer Responses to Integrated Risk-Benefit Information Associated with the Consumption of Food. Risk Analysis. 2011;31(3):429–39. 10.1111/j.1539-6924.2010.01505.x 20880220

[48] Jones P, Niemi JK, Tranter R. List of stakeholder preferred interventions. 2016.

[49] Benard M, de Cock Buning T. Exploring the Potential of Dutch Pig Farmers and Urban-Citizens to Learn Through Frame Reflection. Journal of Agricultural and Environmental Ethics. 2013;26(5):1015–36.

[50] Gilbert M, Conchedda G, Van Boeckel TP, Cinardi G, Linard C, Nicolas G, et al Income disparities and the global distribution of intensively farmed chicken and pigs. PLoS One. 2015;10(7):e0133381 10.1371/journal.pone.0133381 26230336 PMC4521704

[51] High Level Panel of Experts on Food Security and Nutrition. Sustainable agricultural development for food security and nutrition: what roles for livestock? A report by the High Level Panel of Experts on Food Security and Nutrition of the Committee on World Food Security. 2016.

[52] Steinfeld H, Gerber P. Livestock production and the global environment: Consume less or produce better? Proceedings of the National Academy of Sciences. 2010;107(43):18237–8. 10.1073/pnas.1012541107 PMC2972985 20935253

[53] Corp I. IBM SPSS Statistics for Windows version 20.0. Armonk: New York 2011.

[54] Team RC. R: A language and environment for statistical computing. Vienna, Austria: R Foundation for Statistical Computing; 2015.

[55] Fabrigar LR, Wegener DT, MacCallum RC, Strahan EJ. Evaluating the use of exploratory factor analysis in psychological research. Psychological methods. 1999;4(3):272.

[56] Kaiser HF. An index of factorial simplicity. Psychometrika. 1974;39(1):31–6.

[57] Kaiser HF. The application of electronic computers to factor analysis. Educational and psychological measurement. 1960;20(1):141–51.

[58] Cattell RB. The scree test for the number of factors. Multivariate behavioral research. 1966;1(2):245–76. 10.1207/s15327906mbr0102_10 26828106

[59] Stevens JP. Applied multivariate statistics for the social sciences. 4th edn ed. Mahwah, New Jersey: Lawrence Erlbaum Associated, Inc.; 2002.

[60] Field A. Discovering statistics using SPSS. 4th edn ed. London: Sage Publications; 2013.

[61] Rosseel Y. lavaan: an R package for structural equation modeling and more Version 0.4–9 (BETA). Ghent University; 2011.

[62] Contributors. s. (2016). semTools: Useful tools for structural equation modeling. R package version 0.4–14. R Package available on CRAN2016.

[63] Anderson JC, Gerbing DW. Structural equation modeling in practice: A review and recommended two-step approach. Psychological bulletin. 1988;103(3):411.

[64] Lt Hu, Bentler PM. Cutoff criteria for fit indexes in covariance structure analysis: Conventional criteria versus new alternatives. Structural equation modeling: a multidisciplinary journal. 1999;6(1):1–55.

[65] McDonald RP, Ho M-HR. Principles and practice in reporting structural equation analyses. Psychological methods. 2002;7(1):64 11928891 10.1037/1082-989x.7.1.64

[66] Byrne BM. Structural equation modeling: Perspectives on the present and the future. International Journal of Testing. 2001;1(3–4):327–34.

[67] Kline R. Principles and practice of structural equation modelling. 4th edn ed. London: The Guildford Press; 2016.

[68] Evermann J. Multiple-Group Analysis Using the sem Package in the R System. Structural Equation Modeling: A Multidisciplinary Journal. 2010;17(4):677–702. 10.1080/10705511.2010.510070

[69] Nunnally JC. Psychometric theory. New York McGraw-Hill; 1967.

[70] Peterson RA. A meta-analysis of Cronbach's coefficient alpha. Journal of consumer research. 1994;21(2):381–91.

[71] Broom DM. Animal welfare: an aspect of care, sustainability, and food quality required by the public. Journal of veterinary medical education. 2010;37(1):83–8. 10.3138/jvme.37.1.83 20378884

[72] Lassen J, Sandøe P, Forkman B. Happy pigs are dirty!–conflicting perspectives on animal welfare. Livestock Science. 2006;103(3):221–30.

[73] Breakwell GM. Public perceptions concerning animal vaccination: A case study of foot and mouth 2001. 2003.

[74] Department of Environment FaRA. Avian influenza (bird flu) in winter 2016 to 2017 2017. Available from: https://www.gov.uk/government/news/avian-influenza-bird-flu-in-winter-2016-to-2017.

[75] You KG, Bong CW, Lee CW. Antibiotic resistance and plasmid profiling of Vibrio spp. in tropical waters of Peninsular Malaysia. Environmental monitoring and assessment. 2016;188(3):1–15. 26884358 10.1007/s10661-016-5163-0

[76] Liu Y-Y, Wang Y, Walsh TR, Yi L-X, Zhang R, Spencer J, et al Emergence of plasmid-mediated colistin resistance mechanism MCR-1 in animals and human beings in China: a microbiological and molecular biological study. The Lancet infectious diseases. 2016;16(2):161–8. 10.1016/S1473-3099(15)00424-7 26603172

[77] Conti GO, Copat C, Wang Z, D'Agati P, Cristaldi A, Ferrante M. Determination of illegal antimicrobials in aquaculture feed and fish: an ELISA study. Food Control. 2015;50:937–41.

[78] Zhong Y, Huang Z, Wu L. Identifying critical factors influencing the safety and quality related behaviors of pig farmers in China. Food Control. 2017;73:1532–40.

[79] Van Boeckel TP, Brower C, Gilbert M, Grenfell BT, Levin SA, Robinson TP, et al Global trends in antimicrobial use in food animals. Proceedings of the National Academy of Sciences. 2015;112(18):5649–54. 10.1073/pnas.1503141112 PMC4426470 25792457

[80] Finucane ML, Alhakami A, Slovic P, Johnson SM. The affect heuristic in judgments of risks and benefits. Journal of behavioral decision making. 2000;13(1):1.

[81] Scudamore JM. Consumer attitudes to vaccination of food-producing animals. Revue scientifique et technique (International Office of Epizootics). 2007;26(2):451–9. 17892165

[82] Asveld L, Ganzevles J, Osseweijer P. Trustworthiness and responsible research and innovation: the case of the bio-economy. Journal of Agricultural and Environmental Ethics. 2015;28(3):571–88.

[83] Van Kleef E, Houghton JR, Krystallis A, Pfenning U, Rowe G, Van Dijk H, et al Consumer evaluations of food risk management quality in Europe. Risk Analysis. 2007;27(6):1565–80. 10.1111/j.1539-6924.2007.00989.x 18093053

[84] Griffiths R. British Poultry Sector reduces antibiotic use by 44%. British Poultry Council. 2016 27th 7.

[85] Grunert KG, Bredahl L, Brunsø K. Consumer perception of meat quality and implications for product development in the meat sector—a review. Meat science. 2004;66(2):259–72. 10.1016/S0309-1740(03)00130-X 22064127

[86] Lassoued R, Hobbs JE. Consumer confidence in credence attributes: The role of brand trust. Food Policy. 2015;52:99–107.

[87] Frewer L, Salter B. Public attitudes, scientific advice and the politics of regulatory policy: the case of BSE. Science and public policy. 2002;29(2):137–45.

