# Supplementary material for: Consumer attitudes towards production diseases in intensive production systems
Source: PLoS One. 2019 Jan 10;14(1):e0210432. doi: 10.1371/journal.pone.0210432 (PMC6328233; doi:10.1371/journal.pone.0210432)
Supplement: S1 File — (DOCX) [file pone.0210432.s001.docx]

# Supporting information

## A: Constructs, items and rationale for the survey

***Table A Constructs, items and rationale for the survey***

| Name of Scale | Source | Question Asked | Example Item | Response | Rationale |
| --- | --- | --- | --- | --- | --- |
| General concern | [1] | *To what extent do you agree or disagree that you are concerned about the…* | Use of antibiotics in production animals to treat diseases. | Five point Likert scale anchored by Strongly disagree – Strongly agree | A number of concerns in relation to modern animal production were identified in [2, 3]. |
| Human health concern | [1] | *To what extent do you agree or disagree that the following pose a risk to human health* | Use of antibiotics in production animals to treat diseases | Five point Likert scale anchored by Strongly disagree – Strongly agree | [2] identified modern animal production raised several concerns in relation to human health. |
| Animal health concern | [1] | *To what extent do you agree or disagree that the following pose a risk to animal health* | Use of antibiotics in production animals to treat diseases | Five point Likert scale anchored by Strongly disagree – Strongly agree | [2, 3] identified modern animal production raised a number of concerns in relation to animal health. |
| Risk perception | [4-7] | *Intensive farming systems offer …* | Increased incidence of animal diseases.  Reduced consumer health. | Five point Likert scale anchored by Strongly disagree – Strongly agree | Perceived risks have been shown to influence attitudes [4] |
| Benefit perception | [4-6] | *Intensive farming systems offer …* | Improved animal physical health.  Faster treatment of animal diseases. | Five point Likert scale anchored by Strongly disagree – Strongly agree | Perceived benefits have been shown to influence attitudes [4] |
| Trust and responsibility | [1, 4, 8-10] | *To what extent do you agree/ disagree that each of the following sources …* | Can be trusted to act to prevent animal diseases in production systems.  Is responsible for the prevention of production diseases. | Five point Likert scale anchored by Strongly disagree – Strongly agree | Trust has been shown to be important in relation to perceived risks and benefits, and perceptions of FAW [11]. |
| Attitude to intensive animal production | [4, 5, 12] | *Intensive animal production systems are …* | Worthless to valuable.  Bad to good. | Five-point dichotomous scale | Attitudes are known to influence behavioural intention [13-15]. Reviews by [2, 3] indicated a number of items to include. |
| Intention to purchase | [4, 16] | *To what extent to you agree or disagree that …* | I intend to purchase  I would consider purchasing  I am definitely going to purchase | Five point Likert scale anchored by Strongly disagree – Strongly agree | Behavioural intention has been shown to predict behaviour [13-15]. |
| Familiarity and knowledge | [17, 18] | *Do you currently have or have had connections to raising farm (production) animals?*  *I would describe myself as …* | Paying a high degree of attention to the media | Categorical | Familiarity and knowledge are known to influence perceptions of FAW [2]. |
| Attitudes to different interventions | [19] | *To what extent do you agree or disagree that these are acceptable?*  *Which of the following reasons influenced your choice the most?* | Adjustments to feed composition  Use of feed supplements e.g. probiotics  Changes in the amount and time of light provision | Five point Likert scale anchored by Strongly disagree – Strongly agree  Categorical | Attitude to different interventions may vary as per a recent stakeholder survey [19]. |
| Likelihood of different interventions |  | *How likely do you think it will be that these interventions will be used?* | Adjustments to feed composition  Use of feed supplements e.g. probiotics | Five point Likert scale anchored by Very unlikely – Very likely | Perceived likelihood of different interventions may vary as per a recent stakeholder survey [19]. |
| Perceived ethical obligation | [20, 21] | I feel that I have an ethical obligation to … | Avoid animal products from intensive production systems | Five point Likert scale anchored by Strongly disagree – Strongly agree | Attitude has been shown to be influenced by perceived ethical obligation, and attitudes towards FAW closely related to individuals ethical beliefs [22, 23]. |
| Self-identity | [20, 21] | *I think of myself as someone who is …* | Concerned about intensive animal production systems  Farm animal welfare | Five point Likert scale anchored by Strongly disagree – Strongly agree | Attitude has been shown to be influenced by self-identity, and attitudes towards FAW closely related to individuals beliefs [22, 23]. |
| Responsibility for costs |  | *Which actor(s) in the food chain do you think should be responsible for bearing the costs?* | Livestock farmers/ producers through making less profit | Categorical yes or no | Perceived responsibility has been shown to vary between stakeholders in relation to FAW [2] |
| Age | [24, 25] | *What is your age?* | 18-24 years’ old  25-34 years’ old  35-44 years’ old | Categorical | Concern over FAW varies with age [2, 3] |
| Gender | None | *Gender* | Male  Female  I do not wish to specify | Categorical | Concern over FAW varies between genders [2, 3]. |
| Income | [26] | *What is your yearly household income before tax?* | <€10-000  €10,001-€20,000  I do not wish to specify | Categorical – tailored to each country’s currency | Concern over FAW varies with income [2, 3]. |
| Education | [27] | *Which of the following best describes your highest level of education attained?* | Less than primary education  Primary education  Secondary education | Categorical based on ISCED categories. | Concern over FAW varies with education [2, 3]. |
| Place of residence | [28, 29] | *How would you describe where you live?* | City centre  Town or suburb  Rural area | Categorical based on EU definition of | Place of residence may influence familiarity with farming [2], |
| Religion | [30] | *Which of the following best describes your religion?* | Christian  Atheist | Categorical – based on EU breakdown of | Concern over FAW varies by religion [2]. |
| Diet |  | *Which of the below best describes your dietary choices?* | I eat meat and plants  I am vegetarian | Categorical, multiple answer | Concern over FAW varies by diet [3]. |
| Household composition |  | *How many persons are there in your household?*  *How many persons under 18 years old are there in your household?* |  | Open ended | Household composition may influence purchase intentions. |
| Responsibility for shopping |  | *Do you have the main responsibility for shopping in your household?* |  | Categorical | Responsibility for shopping is important when measure purchase (behavioural) intention. |
| Employment |  | *What is your employment status?* | Employed full time  Employed part time | Categorical | Employment can act as a proxy where income is not disclosed. |

## B: Blank survey instruments

## Broilers

Dear Sir/ Madam,

We write to invite you to take part in a research project which aims to understand what the European public think about chicken production systems in Europe. We are asking you in our survey for your views on ways of reducing production diseases in broiler chickens, which are chickens raised for meat production.

Production diseases usually originate from a complex interaction of the viruses and bacteria which are present on farms, animal genetics and the environment in which the animal is reared, including the characteristics of housing, feed and management practices used. They differ from epidemic diseases (such as foot and mouth disease or avian influenza) which are caused by new infections from outside the farm.

Answering our questions will take around 30 minutes. Participation in the study is voluntary and you have the right to decline the invitation or to withdraw from the study at any time. Your answers will be recorded and analyzed. Responses will be treated confidentially and reported so that individual respondents cannot be identified. The results will be used for research purposes only.

After completion of the survey, as a token of our thanks, you will be entered into a prize draw to win a £50 voucher. We thank you in advance for your time and contributions to this research.

Yours faithfully,

Professor Lynn J. Frewer

Newcastle University

[prohealth@newcastle.ac.uk](mailto:prohealth@newcastle.ac.uk)

By ticking this box, I agree to consent to take part in this research.


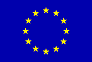
This survey is part of the PROHEALTH project which has received funding from the European Union’s Seventh Framework Programme (FP7/2007-2013) for research, technological development and demonstration under Grant Agreement n°613574. This research is funded by the European Commission project “PROHEALTH” (<http://www.fp7-prohealth.eu/>).

**1 Do you currently have or have had connections to raising farm (production) animals?** Please tick

| Yes, I currently live or have lived on a farm raising production animals |  |
| --- | --- |
| Yes, I currently work or have worked on a farm raising production animals |  |
| Yes, my family or close friends live or have lived on a farm raising production animals |  |
| No |  |

**2 Have you visited a working broiler chicken farm in the last 5 years?** Please tick

| Yes |  |
| --- | --- |
| No |  |

**3 Have you seen or heard anything about production diseases in broiler chicken production systems from any of the sources listed below in the past 3 months?** Please tick.

| **Source of information** | **Yes** | **No** |
| --- | --- | --- |
| Television |  |  |
| Radio |  |  |
| Newspaper |  |  |
| Magazine |  |  |
| The internet |  |  |
| Social media |  |  |
| Friends or family members |  |  |

**4 To what extent do you agree or disagree that the following organisations or individuals should take action for ensuring the *health* of broiler chickens** Please tick one box in each row.

|  | **Strongly Disagree** | **Disagree** | **Neither agree nor disagree** | **Agree** | **Strongly Agree** |
| --- | --- | --- | --- | --- | --- |
|  | **1** | **2** | **3** | **4** | **5** |
| European Commission |  |  |  |  |  |
| National government |  |  |  |  |  |
| Animal health authorities e.g. RSPCA |  |  |  |  |  |
| Veterinarians |  |  |  |  |  |
| Animal welfare organisations |  |  |  |  |  |
| Veterinary medicine producers |  |  |  |  |  |
| Animal breeding companies |  |  |  |  |  |
| Animal feed producers |  |  |  |  |  |
| Animal housing manufacturers |  |  |  |  |  |
| Farmers |  |  |  |  |  |
| Animal Transporters |  |  |  |  |  |
| Slaughterhouses |  |  |  |  |  |
| Quality assurance systems e.g. Freedom Food |  |  |  |  |  |
| Food manufacturers |  |  |  |  |  |
| Food retailers |  |  |  |  |  |
| Consumer organisations |  |  |  |  |  |
| The general public |  |  |  |  |  |
| You as a consumer |  |  |  |  |  |

**5 To what extent do you agree or disagree that the following organisations or individuals should take action for ensuring the *welfare* of broiler chickens** Please tick one box in each row.

|  | **Strongly Disagree** | **Disagree** | **Neither agree nor disagree** | **Agree** | **Strongly Agree** |
| --- | --- | --- | --- | --- | --- |
|  | **1** | **2** | **3** | **4** | **5** |
| European Commission |  |  |  |  |  |
| National government |  |  |  |  |  |
| Animal health authorities |  |  |  |  |  |
| Veterinarians |  |  |  |  |  |
| Animal welfare organisations e.g. RSPCA |  |  |  |  |  |
| Veterinary medicine producers |  |  |  |  |  |
| Animal breeding companies |  |  |  |  |  |
| Animal feed producers |  |  |  |  |  |
| Animal housing manufacturers |  |  |  |  |  |
| Farmers |  |  |  |  |  |
| Animal Transporters |  |  |  |  |  |
| Slaughterhouses |  |  |  |  |  |
| Quality assurance systems e.g. Freedom Food |  |  |  |  |  |
| Food manufacturers |  |  |  |  |  |
| Food retailers |  |  |  |  |  |
| Consumer organisations |  |  |  |  |  |
| The general public |  |  |  |  |  |
| You as a consumer |  |  |  |  |  |

**6 Below is a list of organisations and individuals. We would like you to tell us the extent to which you agree or disagree that they can be trusted to provide the public with accurate information about production diseases in broiler chicken production systems?** Please tick one box in each row.

| **Information source** | **Strongly disagree** | **Disagree** | **Neither agree nor disagree** | **Agree** | **Strongly agree** |
| --- | --- | --- | --- | --- | --- |
|  | **1** | **2** | **3** | **4** | **5** |
| European Commission |  |  |  |  |  |
| National government |  |  |  |  |  |
| Animal health authorities |  |  |  |  |  |
| Veterinarians |  |  |  |  |  |
| Animal welfare organisations e.g. RSPCA |  |  |  |  |  |
| Veterinary medicine producers |  |  |  |  |  |
| Animal breeding companies |  |  |  |  |  |
| Animal feed producers |  |  |  |  |  |
| Animal housing manufacturers |  |  |  |  |  |
| Farmers |  |  |  |  |  |
| Animal Transporters |  |  |  |  |  |
| Slaughterhouses |  |  |  |  |  |
| Quality assurance systems e.g. Freedom Food |  |  |  |  |  |
| Food manufacturers |  |  |  |  |  |
| Food retailers |  |  |  |  |  |
| Consumer organisations |  |  |  |  |  |
| Social media, e.g. Twitter |  |  |  |  |  |
| Traditional media, e.g. newspapers |  |  |  |  |  |

**7 Please rate how you feel about intensive broiler chicken production systems by putting one tick for each row.**

Intensive production systems are associated with a change towards more confined production systems with fewer production units (farms), and a large increase in the number of animals within these. Animals within these systems are generally raised in large numbers, in specialised indoor environments that offer a higher degree of environmental control.

Intensive broiler production systems are …

| Unpleasant | Fairly unpleasant | Neither unpleasant nor pleasant | Fairly pleasant | Pleasant |
| --- | --- | --- | --- | --- |
|  |  |  |  |  |

| Good | Fairly good | Neither good nor bad | Fairly bad | Bad |
| --- | --- | --- | --- | --- |
|  |  |  |  |  |

| Worthless | Fairly worthless | Neither worthless nor valuable | Fairly valuable | Valuable |
| --- | --- | --- | --- | --- |
|  |  |  |  |  |

| Useful | Fairly useful | Neither useful nor useless | Fairly useless | Useless |
| --- | --- | --- | --- | --- |
|  |  |  |  |  |

| Unsafe | Fairly unsafe | Neither safe nor unsafe | Fairly safe | Safe |
| --- | --- | --- | --- | --- |
|  |  |  |  |  |

| Ethical | Fairly ethical | Neither ethical nor unethical | Fairly unethical | Unethical |
| --- | --- | --- | --- | --- |
|  |  |  |  |  |

**8 To what extent do you agree or disagree that intensive broiler chicken production systems offer the following benefits compared to non-intensive broiler chicken production systems:** Please tick one box for each row.

| **Intensive broiler chicken systems are associated with:** | **Strongly disagree** | **Disagree** | **Neither agree nor disagree** | **Agree** | **Strongly agree** |
| --- | --- | --- | --- | --- | --- |
| Reduced animal stress |  |  |  |  |  |
| Reduced incidence of animal diseases |  |  |  |  |  |
| Faster treatment of animal diseases |  |  |  |  |  |
| Improved animal welfare monitoring |  |  |  |  |  |
| Improved human food safety |  |  |  |  |  |
| Improved human food quality |  |  |  |  |  |
| Improved nutritional quality of human food |  |  |  |  |  |
| Improved consumer health |  |  |  |  |  |
| Cheaper food of animal origin |  |  |  |  |  |
| Increased availability of animal-based food products |  |  |  |  |  |
| Benefits to the environment e.g. reduced CO_2_ footprint |  |  |  |  |  |
| A more sustainable approach to animal production |  |  |  |  |  |
| A more cost-efficient production method |  |  |  |  |  |
| Greater protection from predators |  |  |  |  |  |
| Greater protection from bad weather |  |  |  |  |  |
| More professionally run livestock farms |  |  |  |  |  |
| Benefits to agriculture |  |  |  |  |  |
| Benefits to you personally |  |  |  |  |  |
| Benefits to your family |  |  |  |  |  |
| Benefits to consumers |  |  |  |  |  |
| Increased consumer trust in the food they buy |  |  |  |  |  |
| A natural production method |  |  |  |  |  |

**9 Please indicate the extent to which you agree or disagree that intensive broiler chicken production systems are associated with the following risks compared to non-intensive broiler chicken production systems:** Please tick one box in each row.

| **Intensive broiler chicken systems are associated with:** | **Strongly disagree** | **Disagree** | **Neither agree nor disagree** | **Agree** | **Strongly agree** |
| --- | --- | --- | --- | --- | --- |
|  | **1** | **2** | **3** | **4** | **5** |
| Increased animal stress |  |  |  |  |  |
| Increased incidence of animal diseases |  |  |  |  |  |
| Slower treatment of animal diseases |  |  |  |  |  |
| Compromised animal welfare monitoring |  |  |  |  |  |
| Reduced human food safety |  |  |  |  |  |
| Reduced human food quality |  |  |  |  |  |
| Reduced nutritional quality of human food |  |  |  |  |  |
| Negative effects on consumer health |  |  |  |  |  |
| More expensive food of animal origin |  |  |  |  |  |
| Decreased availability of animal-based food products |  |  |  |  |  |
| Risks to the environment e.g. increased CO_2_ footprint |  |  |  |  |  |
| An unsustainable approach to animal production |  |  |  |  |  |
| A non-cost-efficient method of production |  |  |  |  |  |
| Risks to agriculture |  |  |  |  |  |
| Less protection from predators |  |  |  |  |  |
| Less protection from bad weather |  |  |  |  |  |
| Less professionally run livestock farms |  |  |  |  |  |
| Risks to you personally |  |  |  |  |  |
| Risks to your family |  |  |  |  |  |
| Risks to consumers |  |  |  |  |  |
| Decreased consumer trust in the food they buy |  |  |  |  |  |
| An unnatural production method |  |  |  |  |  |

**10 Please indicate to what extent you agree or disagree with the following statements.** Please tick one box in each row.

| **I am concerned about:** | **Strongly disagree** | **Disagree** | **Neither agree nor disagree** | **Agree** | **Strongly agree** |
| --- | --- | --- | --- | --- | --- |
| The current minimum animal welfare standards associated with broiler chicken production |  |  |  |  |  |
| Whether minimum animal welfare standards are actually achieved in broiler chicken production systems |  |  |  |  |  |
| Use of antibiotics in production animals as a growth promoter ^1^ |  |  |  |  |  |
| Use of antibiotics in production animals to prevent diseases |  |  |  |  |  |
| Use of antibiotics in production animals to treat diseases |  |  |  |  |  |
| Use of vaccinations to prevent animal diseases |  |  |  |  |  |
| Use of other veterinary medicines to treat animal diseases |  |  |  |  |  |
| Use of probiotics^2^ to prevent animal diseases |  |  |  |  |  |
| Antibiotic residues in foods |  |  |  |  |  |
| Impacts of animal diseases on human health |  |  |  |  |  |
| Impacts of animal diseases on animal welfare |  |  |  |  |  |
| Impact of animal diseases on food quality |  |  |  |  |  |
| Impact on animal diseases on food safety |  |  |  |  |  |
| Impact of animal diseases on the environment |  |  |  |  |  |
| Antibiotic resistance as a result of the use of antibiotics in animals |  |  |  |  |  |
| Animal production diseases in general |  |  |  |  |  |

**^1^Antiobiotic use as a growth promoter has been banned within the EU since 2006, but is still allowed in other parts of the world**

^2^Probiotics are microorganisms, such as bacteria and yeast, introduced to the body for their potentially beneficial properties.

**11 Please indicate to what extent you agree or disagree with the following statements.** Please tick one box in each row.

| **The following pose a risk to human health** | **Strongly disagree** | **Disagree** | **Neither agree nor disagree** | **Agree** | **Strongly agree** |
| --- | --- | --- | --- | --- | --- |
| The current minimum animal welfare standards associated with broiler chicken production |  |  |  |  |  |
| Whether minimum animal welfare standards are actually achieved in broiler chicken production systems |  |  |  |  |  |
| Use of antibiotics in production animals as a growth promoter |  |  |  |  |  |
| Use of antibiotics in production animals to prevent diseases |  |  |  |  |  |
| Use of antibiotics in production animals to treat diseases |  |  |  |  |  |
| Use of vaccinations to prevent animal diseases |  |  |  |  |  |
| Use of other veterinary medicines to treat animal diseases |  |  |  |  |  |
| Use of probiotics to prevent animal diseases |  |  |  |  |  |
| Antibiotic residues in foods |  |  |  |  |  |
| Impact of animal diseases on food quality |  |  |  |  |  |
| Impact on animal diseases on food safety |  |  |  |  |  |
| Antibiotic resistance as a result of the use of antibiotics in animals |  |  |  |  |  |
| Animal production diseases in general |  |  |  |  |  |

**12 Please indicate to what extent you agree or disagree with the following statements.** Please tick one box in each row.

| **The following pose a risk to animal health:** | **Strongly disagree** | **Disagree** | **Neither agree nor disagree** | **Agree** | **Strongly agree** |
| --- | --- | --- | --- | --- | --- |
| The current minimum animal welfare standards associated with broiler chicken production |  |  |  |  |  |
| Whether minimum animal welfare standards are actually achieved in broiler chicken production systems |  |  |  |  |  |
| Use of antibiotics in production animals as a growth promoter |  |  |  |  |  |
| Use of antibiotics in production animals to prevent diseases |  |  |  |  |  |
| Use of antibiotics in production animals to treat diseases |  |  |  |  |  |
| Use of vaccinations to prevent animal diseases |  |  |  |  |  |
| Use of other veterinary medicines to treat animal diseases |  |  |  |  |  |
| Use of probiotics to prevent animal diseases |  |  |  |  |  |
| Antibiotic residues in animal feeds |  |  |  |  |  |
| Antibiotic resistance as a result of the use of antibiotics in animals |  |  |  |  |  |
| Animal production diseases in general |  |  |  |  |  |

**13 A number of interventions can be used to prevent production diseases and treat sick birds in broiler chicken production systems. To what extent do you agree or disagree that these are acceptable?** Please tick one box in each row.

| **Interventions** | **Strongly disagree** | **Disagree** | **Neither agree nor disagree** | **Agree** | **Strongly agree** |
| --- | --- | --- | --- | --- | --- |
| Adjustments in the quantity of feed available |  |  |  |  |  |
| Adjustments to feed composition |  |  |  |  |  |
| Changes in the amount and time of light provision |  |  |  |  |  |
| Enhanced control of air movement in chicken houses |  |  |  |  |  |
| Enhanced maintenance of the quality of the bedding |  |  |  |  |  |
| Enhanced hygiene to prevent diseases |  |  |  |  |  |
| Housing that allows birds greater freedom to move |  |  |  |  |  |
| The preventive use of veterinary drugs, including antibiotics |  |  |  |  |  |
| Use of feed supplements e.g. probiotics |  |  |  |  |  |
| Providing farmers with a price premium that encourages enhanced animal health |  |  |  |  |  |
| Providing materials and an environment where birds can perform natural behaviors |  |  |  |  |  |
| Improvements in housing design |  |  |  |  |  |
| Housing that protects the birds from adverse natural conditions |  |  |  |  |  |
| Reducing the number of chickens in a given area |  |  |  |  |  |
| Using antibiotics and medicines to treat sick birds |  |  |  |  |  |
| The use of vaccination |  |  |  |  |  |
| Doing nothing |  |  |  |  |  |

**14 For each of the proposed interventions in question 13, please indicate which of the following reasons influenced your choice the most.** Please tick one box in each row.

| **Interventions** | **Naturalness** | **Animal experience** | **Food safety** | **Humane animal care** | **Other** |
| --- | --- | --- | --- | --- | --- |
| Adjustments in the quantity of feed available |  |  |  |  |  |
| Adjustments to feed composition |  |  |  |  |  |
| Changes in the amount and time of light provision |  |  |  |  |  |
| Enhanced control of air movement in chicken houses |  |  |  |  |  |
| Enhanced maintenance of the quality of the bedding |  |  |  |  |  |
| Enhanced hygiene and disease prevention measures |  |  |  |  |  |
| Housing that allows birds greater freedom to move |  |  |  |  |  |
| The preventive use of veterinary drugs, including antibiotics |  |  |  |  |  |
| Use of feed supplements e.g. probiotics |  |  |  |  |  |
| Providing farmers with a price premium that encourages enhanced animal health |  |  |  |  |  |
| Providing materials and an environment where birds can perform natural behaviors |  |  |  |  |  |
| Improvements in housing design |  |  |  |  |  |
| Housing that protects the birds from adverse natural conditions |  |  |  |  |  |
| Reducing the number of chickens in a given area |  |  |  |  |  |
| Using antibiotics and medicines to treat sick birds |  |  |  |  |  |
| The use of vaccination |  |  |  |  |  |
| Doing nothing |  |  |  |  |  |

**15 How likely do you think it is that these interventions will be used. Please indicate your opinion below** Please tick one box in each row.

| **Interventions** | **Very unlikely** | **Unlikely** | **Neither likely nor unlikely** | **Likely** | **Very likely** |
| --- | --- | --- | --- | --- | --- |
| Adjustments in the quantity of feed available |  |  |  |  |  |
| Adjustments to feed composition |  |  |  |  |  |
| Changes in the amount and time of light provision |  |  |  |  |  |
| Enhanced control of air movement in chicken houses |  |  |  |  |  |
| Enhanced maintenance of the quality of the bedding |  |  |  |  |  |
| Enhanced hygiene to prevent diseases |  |  |  |  |  |
| Housing that allows birds greater freedom to move |  |  |  |  |  |
| The preventive use of veterinary drugs, including antibiotics |  |  |  |  |  |
| Use of feed supplements e.g. probiotics |  |  |  |  |  |
| Providing farmers with a price premium that encourages enhanced animal health |  |  |  |  |  |
| Providing materials and an environment where birds can perform natural behaviors |  |  |  |  |  |
| Improvements in housing design |  |  |  |  |  |
| Housing that protects the birds from adverse natural conditions |  |  |  |  |  |
| Reducing the number of chickens in a given area |  |  |  |  |  |
| Using antibiotics and medicines to treat sick birds |  |  |  |  |  |
| The use of vaccination |  |  |  |  |  |
| Doing nothing |  |  |  |  |  |

**16 Please indicate extent to which you agree or disagree with the following statements by ticking one box in each row.**

|  | **Strongly disagree** | **Disagree** | **Neither agree nor disagree** | **Agree** | **Strongly agree** |
| --- | --- | --- | --- | --- | --- |
| I purchase foods produced using intensive production systems |  |  |  |  |  |
| I intend to purchase foods produced using intensive production systems |  |  |  |  |  |
| I would consider purchasing foods produced by intensive production systems |  |  |  |  |  |
| I plan to reduce my consumption of foods from intensive production systems |  |  |  |  |  |
| I avoid purchasing foods from intensive production systems |  |  |  |  |  |
| I feel that I have an obligation to ***purchase*** animal products from intensive production systems |  |  |  |  |  |
| I feel that I have an ethical obligation to ***avoid*** animal products from intensive production systems |  |  |  |  |  |
| I think of myself as someone who is concerned about intensive animal production systems |  |  |  |  |  |
| I think of myself as someone who is concerned about farm animal welfare |  |  |  |  |  |

**17 Which actor(s) in the food chain do you think should bear the costs incurred as a result of any interventions introduced to prevent production diseases in intensive production systems?** Please tick one box in each row.

| **Stakeholder** | **Yes** | **No** |
| --- | --- | --- |
| Livestock farmers/ producers through making less profit |  |  |
| Food manufacturers through making less profit |  |  |
| Food retailers through making less profit |  |  |
| Consumers, through higher product prices |  |  |
| The general public, through increased taxes |  |  |
| National government through providing subsidies and funds |  |  |
| European Commission, through providing subsidies |  |  |

**18 Gender**

| Male |  |
| --- | --- |
| Female |  |
| I do not wish to specify |  |

**19 How old are you?**

|  |
| --- |

Years

**20 Which of the following best describe your highest education level attained.**

| Less than primary education |  |
| --- | --- |
| Primary education |  |
| Secondary education |  |
| Vocational education |  |
| University education |  |

**21 What is your yearly household income before tax?**

| < £8,000 |  |
| --- | --- |
| £8,001 - £16,000 |  |
| £16,001 - £32,000 |  |
| £32,001 -£40,000 |  |
| £40,001 -£64,000 |  |
| > £64,001 |  |
| I do not wish to specify |  |

**22 Which of the below describes your dietary choices?** Tick all that apply

| I eat pork |  |
| --- | --- |
| I eat poultry meat |  |
| I eat eggs |  |
| I eat meat and plants |  |
| I am vegetarian |  |
| I am vegan |  |
| I do not wish to specify |  |

**23 How many persons are there in your household?**

|  |
| --- |

**24 How many persons under 18 years old are there in your household?**

|  |
| --- |

**25 Are you the person who does the main food shopping in your household?**

| Yes |  |
| --- | --- |
| No |  |
| Joint responsibility |  |

**26 How would you describe where you live?**

| City centre |  |
| --- | --- |
| Town or suburb |  |
| Rural area |  |

**26 Which of the following best describes your religious beliefs?**

| Buddhist |  |
| --- | --- |
| Christian |  |
| Hindu |  |
| Jewish |  |
| Muslim |  |
| Sikh |  |
| Other |  |
| Atheist/ agnostic |  |
| I do not wish to specify |  |

**27 What is your employment status?** *Tick all that apply*

| Employed full-time |  |
| --- | --- |
| Employed part-time |  |
| Retired |  |
| Homemaker |  |
| Student |  |
| Unemployed |  |
| I do not wish to specify |  |

**Thank you for your valuable contribution to our project.**

If you would like to find out more about the project and the research taking place, please visit our website, <http://www.fp7-prohealth.eu/>.

Please remember that you are free to withdraw from this study at any time, without having to provide any underlying reason for doing so, and can so by contacting [prohealth@newcastle.ac.uk](mailto:prohealth@newcastle.ac.uk) .

If you would like to be entered into the prize draw please provide your contact details below.

## Layers

Dear Sir/ Madam,

We write to invite you to take part in a research project which aims to understand what the European public think about chicken production systems in Europe. We are asking you in our survey for your views on ways of reducing production diseases in layer hens, which are chickens used to lay eggs.

Production diseases usually originate from a complex interaction of the viruses and bacteria which are present on farms, animal genetics and the environment in which the animal is reared, including the characteristics of housing, feed and management practices used. They differ from epidemic diseases (such as foot and mouth disease or avian influenza) which are caused by new infections from outside the farm.

Answering our questions will take around 30 minutes. Participation in the study is voluntary and you have the right to decline the invitation or to withdraw from the study at any time. Your answers will be recorded and analyzed. Responses will be treated confidentially and reported so that individual respondents cannot be identified. The results will be used for research purposes only.

After completion of the survey, as a token of our thanks, you will be entered into a prize draw to win a £50 voucher. We thank you in advance for your time and contributions to this research.

Yours faithfully,

Professor Lynn J. Frewer

Newcastle University

[prohealth@newcastle.ac.uk](mailto:prohealth@newcastle.ac.uk)

By ticking this box, I agree to consent to take part in this research.


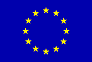
This survey is part of the PROHEALTH project which has received funding from the European Union’s Seventh Framework Programme (FP7/2007-2013) for research, technological development and demonstration under Grant Agreement n°613574. This research is funded by the European Commission project “PROHEALTH” (<http://www.fp7-prohealth.eu/>).

**1 Do you currently have or have had connections to raising farm (production) animals?** Please tick

| Yes, I currently live or have lived on a farm raising production animals |  |
| --- | --- |
| Yes, I currently work or have worked on a farm raising production animals |  |
| Yes, my family or close friends live or have lived on a farm raising production animals |  |
| No |  |

**2 Have you visited a working layer hen farm in the last 5 years?** Please tick

| Yes |  |
| --- | --- |
| No |  |

**3 Have you seen or heard anything about production diseases in layer hen production systems from any of the sources listed below in the past 3 months?** Please tick.

| **Source of information** | **Yes** | **No** |
| --- | --- | --- |
| Television |  |  |
| Radio |  |  |
| Newspaper |  |  |
| Magazine |  |  |
| The internet |  |  |
| Social media |  |  |
| Friends or family members |  |  |

**4 To what extent do you agree or disagree that the following organisations or individuals should take action for ensuring the *health* of layer hens** Please tick one box in each row.

|  | **Strongly Disagree** | **Disagree** | **Neither agree nor disagree** | **Agree** | **Strongly Agree** |
| --- | --- | --- | --- | --- | --- |
|  | **1** | **2** | **3** | **4** | **5** |
| European Commission |  |  |  |  |  |
| National government |  |  |  |  |  |
| Animal health authorities |  |  |  |  |  |
| Veterinarians |  |  |  |  |  |
| Animal welfare organisations e.g. RSPCA |  |  |  |  |  |
| Veterinary medicine producers |  |  |  |  |  |
| Animal breeding companies |  |  |  |  |  |
| Animal feed producers |  |  |  |  |  |
| Animal housing manufacturers |  |  |  |  |  |
| Farmers |  |  |  |  |  |
| Animal Transporters |  |  |  |  |  |
| Slaughterhouses |  |  |  |  |  |
| Quality assurance systems e.g. Freedom Food |  |  |  |  |  |
| Food manufacturers |  |  |  |  |  |
| Food retailers |  |  |  |  |  |
| Consumer organisations |  |  |  |  |  |
| The general public |  |  |  |  |  |
| You as a consumer |  |  |  |  |  |

**5 To what extent do you agree or disagree that the following organisations or individuals should take action for ensuring the *welfare* of layer hens** Please tick one box for each row.

|  | **Strongly Disagree** | **Disagree** | **Neither agree nor disagree** | **Agree** | **Strongly Agree** |
| --- | --- | --- | --- | --- | --- |
|  | **1** | **2** | **3** | **4** | **5** |
| European Commission |  |  |  |  |  |
| National government |  |  |  |  |  |
| Animal health authorities |  |  |  |  |  |
| Veterinarians |  |  |  |  |  |
| Animal welfare organisations e.g. RSPCA |  |  |  |  |  |
| Veterinary medicine producers |  |  |  |  |  |
| Animal breeding companies |  |  |  |  |  |
| Animal feed producers |  |  |  |  |  |
| Animal housing manufacturers |  |  |  |  |  |
| Farmers |  |  |  |  |  |
| Animal Transporters |  |  |  |  |  |
| Slaughterhouses |  |  |  |  |  |
| Quality assurance systems e.g. Freedom Food |  |  |  |  |  |
| Food manufacturers |  |  |  |  |  |
| Food retailers |  |  |  |  |  |
| Consumer organisations |  |  |  |  |  |
| The general public |  |  |  |  |  |
| You as a consumer |  |  |  |  |  |

**6 Below is a list of organisations, individuals and media. We would like you to tell us the extent to which you agree or disagree that they can be trusted to provide the public with accurate information about production diseases in layer hen production systems?** Please tick one box in each row.

| **Information source** | **Strongly disagree** | **Disagree** | **Neither agree nor disagree** | **Agree** | **Strongly agree** |
| --- | --- | --- | --- | --- | --- |
|  | **1** | **2** | **3** | **4** | **5** |
| European Commission |  |  |  |  |  |
| National government |  |  |  |  |  |
| Animal health authorities |  |  |  |  |  |
| Veterinarians |  |  |  |  |  |
| Animal welfare organisations e.g. RSPCA |  |  |  |  |  |
| Veterinary medicine producers |  |  |  |  |  |
| Animal breeding companies |  |  |  |  |  |
| Animal feed producers |  |  |  |  |  |
| Animal housing manufacturers |  |  |  |  |  |
| Farmers |  |  |  |  |  |
| Animal Transporters |  |  |  |  |  |
| Slaughterhouses |  |  |  |  |  |
| Quality assurance systems e.g. Freedom Food |  |  |  |  |  |
| Food manufacturers |  |  |  |  |  |
| Food retailers |  |  |  |  |  |
| Consumer organisations |  |  |  |  |  |
| Social media, e.g. Twitter |  |  |  |  |  |
| Traditional media, e.g. newspapers |  |  |  |  |  |

**7 Please rate how you feel about intensive layer hen production systems by putting one tick for each row.**

Intensive production systems are associated with a change towards more confined production systems with fewer production units (farms), and a large increase in the number of animals within these. Animals within these systems are generally raised in large numbers, in specialised indoor environments that offer a higher degree of environmental control.

Intensive layer hen production systems are …

| Unpleasant | Fairly unpleasant | Neither unpleasant nor pleasant | Fairly pleasant | Pleasant |
| --- | --- | --- | --- | --- |
|  |  |  |  |  |

| Good | Fairly good | Neither good nor bad | Fairly bad | Bad |
| --- | --- | --- | --- | --- |
|  |  |  |  |  |

| Worthless | Fairly worthless | Neither worthless nor valuable | Fairly valuable | Valuable |
| --- | --- | --- | --- | --- |
|  |  |  |  |  |

| Useful | Fairly useful | Neither useful nor useless | Fairly useless | Useless |
| --- | --- | --- | --- | --- |
|  |  |  |  |  |

| Unsafe | Fairly unsafe | Neither safe nor unsafe | Fairly safe | Safe |
| --- | --- | --- | --- | --- |
|  |  |  |  |  |

| Ethical | Fairly ethical | Neither ethical nor unethical | Fairly unethical | Unethical |
| --- | --- | --- | --- | --- |
|  |  |  |  |  |

**8 To what extent do you agree or disagree that intensive layer hen production systems offer the following benefits compared to non-intensive layer hen production systems:** Please tick one box for each row.

| **Intensive layer hen systems are associated with:** | **Strongly disagree** | **Disagree** | **Neither agree nor disagree** | **Agree** | **Strongly agree** |
| --- | --- | --- | --- | --- | --- |
| Reduced animal stress |  |  |  |  |  |
| Reduced incidence of animal diseases |  |  |  |  |  |
| Faster treatment of animal diseases |  |  |  |  |  |
| Improved animal welfare monitoring |  |  |  |  |  |
| Improved human food safety |  |  |  |  |  |
| Improved human food quality |  |  |  |  |  |
| Improved nutritional quality of human food |  |  |  |  |  |
| Improved consumer health |  |  |  |  |  |
| Cheaper food of animal origin |  |  |  |  |  |
| Increased availability of animal-based food products |  |  |  |  |  |
| Benefits to the environment e.g. reduced CO_2_ footprint |  |  |  |  |  |
| A more sustainable approach to animal production |  |  |  |  |  |
| A more cost-efficient production method |  |  |  |  |  |
| Greater protection from predators |  |  |  |  |  |
| Greater protection from bad weather |  |  |  |  |  |
| More professionally run livestock farms |  |  |  |  |  |
| Benefits to agriculture |  |  |  |  |  |
| Benefits to you personally |  |  |  |  |  |
| Benefits to your family |  |  |  |  |  |
| Benefits to consumers |  |  |  |  |  |
| Increased consumer trust in the food they buy |  |  |  |  |  |
| A natural production method |  |  |  |  |  |

**9 Please indicate the extent to which you agree or disagree that intensive layer hen production systems are associated with the following risks compared to non-intensive layer hen production systems:** Please tick one box in each row.

| **Intensive layer hen systems are associated with** | **Strongly disagree** | **Disagree** | **Neither agree nor disagree** | **Agree** | **Strongly agree** |
| --- | --- | --- | --- | --- | --- |
| Increased animal stress |  |  |  |  |  |
| Increased incidence of animal diseases |  |  |  |  |  |
| Slower treatment of animal diseases |  |  |  |  |  |
| Compromised animal welfare monitoring |  |  |  |  |  |
| Reduced human food safety |  |  |  |  |  |
| Reduced human food quality |  |  |  |  |  |
| Reduced nutritional quality of human food |  |  |  |  |  |
| Negative effects on consumer health |  |  |  |  |  |
| More expensive food of animal origin |  |  |  |  |  |
| Decreased availability of animal-based food products |  |  |  |  |  |
| Risks to the environment e.g. increased CO_2_ footprint |  |  |  |  |  |
| An unsustainable approach to animal production |  |  |  |  |  |
| A non-cost-efficient method of production |  |  |  |  |  |
| Risks to agriculture |  |  |  |  |  |
| Less protection from predators |  |  |  |  |  |
| Less protection from bad weather |  |  |  |  |  |
| Less professionally run livestock farms |  |  |  |  |  |
| Risks to you personally |  |  |  |  |  |
| Risks to your family |  |  |  |  |  |
| Risks to consumers |  |  |  |  |  |
| Decreased consumer trust in the food they buy |  |  |  |  |  |
| An unnatural production method |  |  |  |  |  |

**10 Please indicate to what extent you agree or disagree with the following statements.** Please tick one box in each row.

| **I am concerned about:** | **Strongly disagree** | **Disagree** | **Neither agree nor disagree** | **Agree** | **Strongly agree** |
| --- | --- | --- | --- | --- | --- |
| The current minimum animal welfare standards associated with layer hen production |  |  |  |  |  |
| Whether minimum animal welfare standards are actually achieved in layer hen production systems |  |  |  |  |  |
| Use of antibiotics in production animals as a growth promoter^1^ |  |  |  |  |  |
| Use of antibiotics in production animals to prevent diseases |  |  |  |  |  |
| Use of antibiotics in production animals to treat diseases |  |  |  |  |  |
| Use of vaccinations to prevent animal diseases |  |  |  |  |  |
| Use of other veterinary medicines to treat animal diseases |  |  |  |  |  |
| Use of probiotics^2^ to prevent animal diseases |  |  |  |  |  |
| Antibiotic residues in foods |  |  |  |  |  |
| Impacts of animal diseases on human health |  |  |  |  |  |
| Impacts of animal diseases on animal welfare |  |  |  |  |  |
| Impact of animal diseases on food quality |  |  |  |  |  |
| Impact on animal diseases on food safety |  |  |  |  |  |
| Impact of animal diseases on the environment |  |  |  |  |  |
| Antibiotic resistance as a result of the use of antibiotics in animals |  |  |  |  |  |
| Animal production diseases in general |  |  |  |  |  |

**^1^Antiobiotic use as a growth promoter has been banned within the EU since 2006, but is still allowed in other parts of the world**

^2^Probiotics are microorganisms, such as bacteria and yeast, introduced to the body for their potentially beneficial properties.

**11 Please indicate to what extent you agree or disagree with the following statements.** Please tick one box in each row.

| **The following pose a risk to human health** | **Strongly disagree** | **Disagree** | **Neither agree nor disagree** | **Agree** | **Strongly agree** |
| --- | --- | --- | --- | --- | --- |
| The current minimum animal welfare standards associated with layer hen production |  |  |  |  |  |
| Whether minimum animal welfare standards are actually achieved in layer hen production systems |  |  |  |  |  |
| Use of antibiotics in production animals as a growth promoter |  |  |  |  |  |
| Use of antibiotics in production animals to prevent diseases |  |  |  |  |  |
| Use of antibiotics in production animals to treat diseases |  |  |  |  |  |
| Use of vaccinations to prevent animal diseases |  |  |  |  |  |
| Use of other veterinary medicines to treat animal diseases |  |  |  |  |  |
| Use of probiotics to prevent animal diseases |  |  |  |  |  |
| Antibiotic residues in food |  |  |  |  |  |
| Impact of animal diseases on food quality |  |  |  |  |  |
| Impact on animal diseases on food safety |  |  |  |  |  |
| Antibiotic resistance as a result of the use of antibiotics in animals |  |  |  |  |  |
| Animal production diseases in general |  |  |  |  |  |

**12 Please indicate to what extent you agree with the following statements.** Please tick one box in each row.

| **The following pose a risk to animal health:** | **Strongly disagree** | **Disagree** | **Neither agree nor disagree** | **Agree** | **Strongly agree** |
| --- | --- | --- | --- | --- | --- |
| The current minimum animal welfare standards associated with layer hen production |  |  |  |  |  |
| Whether minimum animal welfare standards are actually achieved in layer hen production systems |  |  |  |  |  |
| Use of antibiotics in production animals as a growth promoter |  |  |  |  |  |
| Use of antibiotics in production animals to prevent diseases |  |  |  |  |  |
| Use of antibiotics in production animals to treat diseases |  |  |  |  |  |
| Use of vaccinations to prevent animal diseases |  |  |  |  |  |
| Use of other veterinary medicines to treat animal diseases |  |  |  |  |  |
| Use of probiotics to prevent animal diseases |  |  |  |  |  |
| Antibiotic residues in animal feeds |  |  |  |  |  |
| Antibiotic resistance as a result of the use of antibiotics in animals |  |  |  |  |  |
| Animal production diseases in general |  |  |  |  |  |

**13 A number of interventions can be used to prevent production diseases and treat sick birds in layer hen production systems. To what extent do you agree or disagree that these are acceptable?** Please tick one box in each row.

| **Interventions** | **Strongly disagree** | **Disagree** | **Neither agree nor disagree** | **Agree** | **Strongly agree** |
| --- | --- | --- | --- | --- | --- |
| Adjustments in the quantity of feed available |  |  |  |  |  |
| Adjustments to feed composition |  |  |  |  |  |
| Changes in the amount and time of light provision |  |  |  |  |  |
| Enhanced control of air movement in chicken houses |  |  |  |  |  |
| Enhanced maintenance of the quality of the bedding |  |  |  |  |  |
| Enhanced hygiene and disease prevention measures |  |  |  |  |  |
| Housing that allows birds greater freedom to move |  |  |  |  |  |
| The preventive use of veterinary drugs, including antibiotics |  |  |  |  |  |
| Use of feed supplements e.g. probiotics |  |  |  |  |  |
| Providing farmers with a price premium that encourages enhanced animal health |  |  |  |  |  |
| Providing materials and an environment where birds can perform natural behaviors |  |  |  |  |  |
| Improvements in housing design |  |  |  |  |  |
| Housing that protects the birds from adverse natural conditions |  |  |  |  |  |
| Reducing the number of chickens in a given area |  |  |  |  |  |
| Using antibiotics and medicines to treat sick birds |  |  |  |  |  |
| The use of vaccination |  |  |  |  |  |
| Doing nothing |  |  |  |  |  |

**14 For each of the proposed interventions in question 13, please indicate which of the following reasons influenced your choice the most.** Please tick one box in each row.

| **Interventions** | **Naturalness** | **Animal experience** | **Food safety** | **Humane animal care** | **Other** |
| --- | --- | --- | --- | --- | --- |
| Adjustments in the quantity of feed available |  |  |  |  |  |
| Adjustments to feed composition |  |  |  |  |  |
| Changes in the amount and time of light provision |  |  |  |  |  |
| Enhanced control of air movement in chicken houses |  |  |  |  |  |
| Enhanced maintenance of the quality of the bedding |  |  |  |  |  |
| Enhanced hygiene to prevent diseases |  |  |  |  |  |
| Housing that allows birds greater freedom to move |  |  |  |  |  |
| The preventive use of veterinary drugs, including antibiotics |  |  |  |  |  |
| Use of feed supplements e.g. probiotics |  |  |  |  |  |
| Providing farmers with a price premium that encourages enhanced animal health |  |  |  |  |  |
| Providing materials and an environment where birds can perform natural behaviors |  |  |  |  |  |
| Improvements in housing design |  |  |  |  |  |
| Housing that protects the birds from adverse natural conditions |  |  |  |  |  |
| Reducing the number of chickens in a given area |  |  |  |  |  |
| Using antibiotics and medicines to treat sick birds |  |  |  |  |  |
| The use of vaccination |  |  |  |  |  |
| Doing nothing |  |  |  |  |  |

**15 How likely do you think it is that these interventions will be used. Please indicate your opinion below** Please tick one box in each row.

| **Interventions** | **Very unlikely** | **Unlikely** | **Neither likely nor unlikely** | **Likely** | **Very likely** |
| --- | --- | --- | --- | --- | --- |
| Adjustments in the quantity of feed available |  |  |  |  |  |
| Adjustments to feed composition |  |  |  |  |  |
| Changes in the amount and time of light provision |  |  |  |  |  |
| Enhanced control of air movement in chicken houses |  |  |  |  |  |
| Enhanced maintenance of the quality of the bedding |  |  |  |  |  |
| Enhanced hygiene and disease prevention measures |  |  |  |  |  |
| Housing that allows birds greater freedom to move |  |  |  |  |  |
| The preventive use of veterinary drugs, including antibiotics |  |  |  |  |  |
| Use of feed supplements e.g. probiotics |  |  |  |  |  |
| Providing farmers with a price premium that encourages enhanced animal health |  |  |  |  |  |
| Providing materials and an environment where birds can perform natural behaviors |  |  |  |  |  |
| Improvements in housing design |  |  |  |  |  |
| Housing that protects the birds from adverse natural conditions |  |  |  |  |  |
| Reducing the number of chickens in a given area |  |  |  |  |  |
| Using antibiotics and medicines to treat sick birds |  |  |  |  |  |
| The use of vaccination |  |  |  |  |  |
| Doing nothing |  |  |  |  |  |

**16 Please indicate extent to which you agree or disagree with the following statements by ticking one box in each row.**

|  | **Strongly disagree** | **Disagree** | **Neither agree nor disagree** | **Agree** | **Strongly agree** |
| --- | --- | --- | --- | --- | --- |
| I purchase foods produced using intensive production systems |  |  |  |  |  |
| I intend to purchase foods produced using intensive production systems |  |  |  |  |  |
| I would consider purchasing foods produced by intensive production systems |  |  |  |  |  |
| I plan to reduce my consumption of foods from intensive production systems |  |  |  |  |  |
| I avoid purchasing foods from intensive production systems |  |  |  |  |  |
| I feel that I have an obligation to ***purchase*** animal products from intensive production systems |  |  |  |  |  |
| I feel that I have an ethical obligation to ***avoid*** animal products from intensive production systems |  |  |  |  |  |
| I think of myself as someone who is concerned about intensive animal production systems |  |  |  |  |  |
| I think of myself as someone who is concerned about farm animal welfare |  |  |  |  |  |

**17 Which actor(s) in the food chain do you think should bear the costs incurred as a result of any interventions introduced to prevent production diseases in intensive production systems?** Please tick one box in each row.

| **Stakeholder** | **Yes** | **No** |
| --- | --- | --- |
| Livestock farmers/ producers through making less profit |  |  |
| Food manufacturers through making less profit |  |  |
| Food retailers through making less profit |  |  |
| Consumers, through higher product prices |  |  |
| The general public, through increased taxes |  |  |
| National government through providing subsidies and funds |  |  |
| European Commission, through providing subsidies |  |  |

**18 Gender**

| Male |  |
| --- | --- |
| Female |  |
| I do not wish to specify |  |

**19 How old are you?**

|  |
| --- |

Years

**20 Which of the following best describe your highest education level attained.**

| Less than primary education |  |
| --- | --- |
| Primary education |  |
| Secondary education |  |
| Vocational education |  |
| University education |  |

**21 What is your yearly household income before tax?**

| < £8,000 |  |
| --- | --- |
| £8,001 - £16,000 |  |
| £16,001 - £32,000 |  |
| £32,001 -£40,000 |  |
| £40,001 -£64,000 |  |
| > £64,001 |  |
| I do not wish to specify |  |

**22 Which of the below describes your dietary choices?** Tick all that apply

| I eat pork |  |
| --- | --- |
| I eat poultry meat |  |
| I eat eggs |  |
| I eat meat and plants |  |
| I am vegetarian |  |
| I am vegan |  |
| I do not wish to specify |  |

**23 How many persons are there in your household?**

|  |
| --- |

**24 How many persons under 18 years old are there in your household?**

|  |
| --- |

**25 Are you the person who does the main food shopping in your household?**

| Yes |  |
| --- | --- |
| No |  |
| Joint responsibility |  |

**26 How would you describe where you live?**

| City centre |  |
| --- | --- |
| Town or suburb |  |
| Rural area |  |

**26 Which of the following best describes your religious beliefs?**

| Buddhist |  |
| --- | --- |
| Christian |  |
| Hindu |  |
| Jewish |  |
| Muslim |  |
| Sikh |  |
| Other |  |
| Atheist/ agnostic |  |
| I do not wish to specify |  |

**27 What is your employment status? *Tick all that apply***

| Employed full-time |  |
| --- | --- |
| Employed part-time |  |
| Retired |  |
| Homemaker |  |
| Student |  |
| Unemployed |  |
| I do not wish to specify |  |

**Thank you for your valuable contribution to our project.** If you would like to find out more about the project and the research taking place, please visit our website, <http://www.fp7-prohealth.eu/>. Please remember that you are free to withdraw from this study at any time, without having to provide any underlying reason for doing so, and can so by contacting [prohealth@newcastle.ac.uk](mailto:prohealth@newcastle.ac.uk) . If you would like to be entered into the prize draw please provide your contact details below

## Pigs

Dear Sir/ Madam,

We write to invite you to take part in a research project which aims to understand what the European public think about pig production systems in Europe. We are asking you in our survey for your views on ways of reducing production diseases in pigs.

Production diseases usually originate from a complex interaction of the viruses and bacteria which are present on farms, animal genetics and the environment in which the animal is reared, including the characteristics of housing, feed and management practices used. They differ from epidemic diseases (such as foot and mouth disease or avian influenza) which are caused by new infections from outside the farm.

Answering our questions will take around 30 minutes. Participation in the study is voluntary and you have the right to decline the invitation or to withdraw from the study at any time. Your answers will be recorded and analyzed. Responses will be treated confidentially and reported so that individual respondents cannot be identified. The results will be used for research purposes only.

After completion of the survey, as a token of our thanks, you will be entered into a prize draw to win a £50 voucher. We thank you in advance for your time and contributions to this research.

Yours faithfully,

Professor Lynn J. Frewer

Newcastle University

[prohealth@newcastle.ac.uk](mailto:prohealth@newcastle.ac.uk)

By ticking this box, I agree to consent to take part in this research.


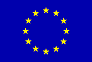
This survey is part of the PROHEALTH project which has received funding from the European Union’s Seventh Framework Programme (FP7/2007-2013) for research, technological development and demonstration under Grant Agreement n°613574. This research is funded by the European Commission project “PROHEALTH” (<http://www.fp7-prohealth.eu/>).

**1 Do you currently have or have had connections to raising farm (production) animals?** Please tick

| Yes, I currently live or have lived on a farm raising production animals |  |
| --- | --- |
| Yes, I currently work or have worked on a farm raising production animals |  |
| Yes, my family or close friends live or have lived on a farm raising production animals |  |
| No |  |

**2 Have you visited a working pig farm in the last 5 years?** Please tick

| Yes |  |
| --- | --- |
| No |  |

**3 Have you seen or heard anything about production diseases in pig production systems from any of the sources listed below in the past 3 months?** Please tick.

| **Source of information** | **Yes** | **No** |
| --- | --- | --- |
| Television |  |  |
| Radio |  |  |
| Newspaper |  |  |
| Magazine |  |  |
| The internet |  |  |
| Social media |  |  |
| Friends or family members |  |  |

**4 To what extent do you agree or disagree that the following organisations or individuals should take action for ensuring the *health* of pigs.** Please tick one box in each row.

|  | **Strongly Disagree** | **Disagree** | **Neither agree nor disagree** | **Agree** | **Strongly Agree** |
| --- | --- | --- | --- | --- | --- |
| European Commission |  |  |  |  |  |
| National government |  |  |  |  |  |
| Animal health authorities |  |  |  |  |  |
| Veterinarians |  |  |  |  |  |
| Animal welfare organisations e.g. RSPCA |  |  |  |  |  |
| Veterinary medicine producers |  |  |  |  |  |
| Animal breeding companies |  |  |  |  |  |
| Animal feed producers |  |  |  |  |  |
| Animal housing manufacturers |  |  |  |  |  |
| Farmers |  |  |  |  |  |
| Animal Transporters |  |  |  |  |  |
| Slaughterhouses |  |  |  |  |  |
| Quality assurance systems e.g. Freedom Food |  |  |  |  |  |
| Food manufacturers |  |  |  |  |  |
| Food retailers |  |  |  |  |  |
| Consumer organisations |  |  |  |  |  |
| The general public |  |  |  |  |  |
| You as a consumer |  |  |  |  |  |

**5 To what extent do you agree or disagree that the following organisations or individuals should take action for ensuring the *welfare* of pigs.** Please tick one box in each row.

|  | **Strongly Disagree** | **Disagree** | **Neither agree nor disagree** | **Agree** | **Strongly Agree** |
| --- | --- | --- | --- | --- | --- |
| European Commission |  |  |  |  |  |
| National government |  |  |  |  |  |
| Animal health authorities |  |  |  |  |  |
| Veterinarians |  |  |  |  |  |
| Animal welfare organisations e.g. RSPCA |  |  |  |  |  |
| Veterinary medicine producers |  |  |  |  |  |
| Animal breeding companies |  |  |  |  |  |
| Animal feed producers |  |  |  |  |  |
| Animal housing manufacturers |  |  |  |  |  |
| Farmers |  |  |  |  |  |
| Animal Transporters |  |  |  |  |  |
| Slaughterhouses |  |  |  |  |  |
| Quality assurance systems e.g. Freedom Food |  |  |  |  |  |
| Food manufacturers |  |  |  |  |  |
| Food retailers |  |  |  |  |  |
| Consumer organisations |  |  |  |  |  |
| The general public |  |  |  |  |  |
| You as a consumer |  |  |  |  |  |

**6 Below is a list of organisations and individuals. We would like you to tell us the extent to which you agree or disagree that they can be trusted to provide the public with accurate information about production diseases in pig production systems?** Please tick one box in each row.

| **Information source** | **Strongly disagree** | **Disagree** | **Neither agree nor disagree** | **Agree** | **Strongly agree** |
| --- | --- | --- | --- | --- | --- |
| European Commission |  |  |  |  |  |
| National government |  |  |  |  |  |
| Animal health authorities |  |  |  |  |  |
| Veterinarians |  |  |  |  |  |
| Animal welfare organisations e.g. RSPCA |  |  |  |  |  |
| Veterinary medicine producers |  |  |  |  |  |
| Animal breeding companies |  |  |  |  |  |
| Animal feed producers |  |  |  |  |  |
| Animal housing manufacturers |  |  |  |  |  |
| Farmers |  |  |  |  |  |
| Animal Transporters |  |  |  |  |  |
| Slaughterhouses |  |  |  |  |  |
| Quality assurance systems e.g. Freedom Food |  |  |  |  |  |
| Food manufacturers |  |  |  |  |  |
| Food retailers |  |  |  |  |  |
| Consumer organisations |  |  |  |  |  |
| Social media, e.g. Twitter |  |  |  |  |  |
| Traditional media, e.g. newspapers |  |  |  |  |  |

**7 Please rate how you feel about intensive pig production systems by putting one tick in each row.**

Intensive production systems are associated with a change towards more confined production systems with fewer production units (farms), and a large increase in the number of animals within these. Animals within these systems are generally raised in large numbers, in specialised indoor environments that offer a higher degree of environmental control.

Intensive pig production systems are …

| Unpleasant | Fairly unpleasant | Neither unpleasant nor pleasant | Fairly pleasant | Pleasant |
| --- | --- | --- | --- | --- |
|  |  |  |  |  |

| Good | Fairly good | Neither good nor bad | Fairly bad | Bad |
| --- | --- | --- | --- | --- |
|  |  |  |  |  |

| Worthless | Fairly worthless | Neither worthless nor valuable | Fairly valuable | Valuable |
| --- | --- | --- | --- | --- |
|  |  |  |  |  |

| Useful | Fairly useful | Neither useful nor useless | Fairly useless | Useless |
| --- | --- | --- | --- | --- |
|  |  |  |  |  |

| Unsafe | Fairly unsafe | Neither safe nor unsafe | Fairly safe | Safe |
| --- | --- | --- | --- | --- |
|  |  |  |  |  |

| Ethical | Fairly ethical | Neither ethical nor unethical | Fairly unethical | Unethical |
| --- | --- | --- | --- | --- |
|  |  |  |  |  |

**8 To what extent do you agree or disagree that intensive pig production systems offer the following benefits, compared to non-intensive pig production systems:** Please tick one box in each row.

| **Intensive pig systems are associated with:** | **Strongly disagree** | **Disagree** | **Neither agree nor disagree** | **Agree** | **Strongly agree** |
| --- | --- | --- | --- | --- | --- |
| Reduced animal stress |  |  |  |  |  |
| Reduced incidence of animal diseases |  |  |  |  |  |
| Faster treatment of animal diseases |  |  |  |  |  |
| Improved animal welfare monitoring |  |  |  |  |  |
| Improved human food safety |  |  |  |  |  |
| Improved human food quality |  |  |  |  |  |
| Improved nutritional quality of human food |  |  |  |  |  |
| Improved consumer health |  |  |  |  |  |
| Cheaper food of animal origin |  |  |  |  |  |
| Increased availability of animal-based food products |  |  |  |  |  |
| Benefits to the environment e.g. reduced CO_2_ footprint |  |  |  |  |  |
| A more sustainable approach to animal production |  |  |  |  |  |
| A more cost-efficient production method |  |  |  |  |  |
| Greater protection from predators |  |  |  |  |  |
| Greater protection from bad weather |  |  |  |  |  |
| More professionally run livestock farms |  |  |  |  |  |
| Benefits to agriculture |  |  |  |  |  |
| Benefits to you personally |  |  |  |  |  |
| Benefits to your family |  |  |  |  |  |
| Benefits to consumers |  |  |  |  |  |
| Increased consumer trust in the food they buy |  |  |  |  |  |
| A natural production method |  |  |  |  |  |

**9 Please indicate the extent to which you agree or disagree that intensive pig production systems are associated with the following risks, compared to non-intensive pig production systems:** Please tick one box in each row.

| **Intensive pig systems are associated with:** | **Strongly disagree** | **Disagree** | **Neither agree nor disagree** | **Agree** | **Strongly agree** |
| --- | --- | --- | --- | --- | --- |
| Increased animal stress |  |  |  |  |  |
| Increased incidence of animal diseases |  |  |  |  |  |
| Slower treatment of animal diseases |  |  |  |  |  |
| Compromised animal welfare monitoring |  |  |  |  |  |
| Reduced human food safety |  |  |  |  |  |
| Reduced human food quality |  |  |  |  |  |
| Reduced nutritional quality of human food |  |  |  |  |  |
| Negative effects on consumer health |  |  |  |  |  |
| More expensive food of animal origin |  |  |  |  |  |
| Decreased availability of animal-based food products |  |  |  |  |  |
| Risks to the environment e.g. increased CO_2_ footprint |  |  |  |  |  |
| An unsustainable approach to animal production |  |  |  |  |  |
| A non-cost-efficient method of production |  |  |  |  |  |
| Less protection from predators |  |  |  |  |  |
| Less protection from bad weather |  |  |  |  |  |
| Less professionally run livestock farms |  |  |  |  |  |
| Risks to agriculture |  |  |  |  |  |
| Risks to you personally |  |  |  |  |  |
| Risks to your family |  |  |  |  |  |
| Risks to consumers |  |  |  |  |  |
| Decreased consumer trust in the food they buy |  |  |  |  |  |
| An unnatural production method |  |  |  |  |  |

**10 Please indicate to what extent you agree or disagree with the following statements.** Please tick one box in each row.

| **I am concerned about:** | **Strongly disagree** | **Disagree** | **Neither agree nor disagree** | **Agree** | **Strongly agree** |
| --- | --- | --- | --- | --- | --- |
| The current minimum animal welfare standards associated with pig production |  |  |  |  |  |
| Whether minimum animal welfare standards are actually achieved in pig production systems |  |  |  |  |  |
| Use of antibiotics in production animals as a growth promoter^1^ |  |  |  |  |  |
| Use of antibiotics in production animals to prevent diseases |  |  |  |  |  |
| Use of antibiotics in production animals to treat diseases |  |  |  |  |  |
| Use of vaccinations to prevent animal diseases |  |  |  |  |  |
| Use of other veterinary medicines to treat animal diseases |  |  |  |  |  |
| Use of probiotics^2^ to prevent animal diseases |  |  |  |  |  |
| Antibiotic residues in foods |  |  |  |  |  |
| Impacts of animal diseases on human health |  |  |  |  |  |
| Impacts of animal diseases on animal welfare |  |  |  |  |  |
| Impact of animal diseases on food quality |  |  |  |  |  |
| Impact on animal diseases on food safety |  |  |  |  |  |
| Impact of animal diseases on the environment |  |  |  |  |  |
| Antibiotic resistance as a result of the use of antibiotics in animals |  |  |  |  |  |
| Animal production diseases in general |  |  |  |  |  |

^1^Antiobiotic use as a growth promoter has been banned within the EU since 2006, but is still allowed in other parts of the world

^2^Probiotics are microorganisms, such as bacteria and yeast, introduced to the body for their potentially beneficial properties.

**11 Please indicate to what extent you agree or disagree with the following statements.** Please tick one box in each row.

| **The following pose a risk to human health** | **Strongly disagree** | **Disagree** | **Neither agree nor disagree** | **Agree** | **Strongly agree** |
| --- | --- | --- | --- | --- | --- |
| The current minimum animal welfare standards associated with pig production |  |  |  |  |  |
| Whether minimum animal welfare standards are actually achieved in pig production systems |  |  |  |  |  |
| Use of antibiotics in production animals as a growth promoter |  |  |  |  |  |
| Use of antibiotics in production animals to prevent diseases |  |  |  |  |  |
| Use of antibiotics in production animals to treat diseases |  |  |  |  |  |
| Use of vaccinations to prevent animal diseases |  |  |  |  |  |
| Use of other veterinary medicines to treat animal diseases |  |  |  |  |  |
| Use of probiotics to prevent animal diseases |  |  |  |  |  |
| Antibiotic residues in food |  |  |  |  |  |
| Impact of animal diseases on food quality |  |  |  |  |  |
| Impact on animal diseases on food safety |  |  |  |  |  |
| Antibiotic resistance as a result of the use of antibiotics in animals |  |  |  |  |  |
| Animal production diseases in general |  |  |  |  |  |

**12 Please indicate to what extent you agree or disagree with the following statements.** Please tick one box in each row.

| **The following pose a risk to animal health:** | **Strongly disagree** | **Disagree** | **Neither agree nor disagree** | **Agree** | **Strongly agree** |
| --- | --- | --- | --- | --- | --- |
| The current minimum animal welfare standards associated with pig production |  |  |  |  |  |
| Whether minimum animal welfare standards are actually achieved in pig production systems |  |  |  |  |  |
| Use of antibiotics in production animals as a growth promoter |  |  |  |  |  |
| Use of antibiotics in production animals to prevent diseases |  |  |  |  |  |
| Use of antibiotics in production animals to treat diseases |  |  |  |  |  |
| Use of vaccinations to prevent animal diseases |  |  |  |  |  |
| Use of other veterinary medicines to treat animal diseases |  |  |  |  |  |
| Use of probiotics to prevent animal diseases |  |  |  |  |  |
| Antibiotic residues in animal feeds |  |  |  |  |  |
| Antibiotic resistance as a result of the use of antibiotics in animals |  |  |  |  |  |
| Animal production diseases in general |  |  |  |  |  |

**13 A number of interventions can be used to prevent production diseases and treat sick animals in pig production systems. To what extent do you agree or disagree that these are acceptable?** Please tick one box in each row

| **Interventions** | **Strongly disagree** | **Disagree** | **Neither agree nor disagree** | **Agree** | **Strongly agree** |
| --- | --- | --- | --- | --- | --- |
| Enhanced hygiene and disease prevention measures |  |  |  |  |  |
| Using medicines and antibiotics to treat sick pigs |  |  |  |  |  |
| The preventive use of veterinary drugs, including antibiotics |  |  |  |  |  |
| Use of feed supplements e.g. probiotics |  |  |  |  |  |
| The use of vaccination |  |  |  |  |  |
| Efficient monitoring of pigs and pig housing conditions |  |  |  |  |  |
| Enhanced control of air movement in pig houses |  |  |  |  |  |
| Improvements in pigs’ diet composition |  |  |  |  |  |
| Adjustments in the quantity of pig feed available |  |  |  |  |  |
| Breeding for genetically tougher or more resilient pigs |  |  |  |  |  |
| Improvements in housing design |  |  |  |  |  |
| Housing that protects the pigs from adverse natural conditions |  |  |  |  |  |
| Reducing the number of pigs in a given area |  |  |  |  |  |
| Providing enrichment materials so pigs can perform natural behaviors |  |  |  |  |  |
| Providing farmers with a price premium that encourages enhanced animal health |  |  |  |  |  |
| Doing nothing |  |  |  |  |  |

**14 For each of these proposed interventions in question 13, please indicate which of the following reasons influenced your choice the most.** Please tick one box in each row.

| **Interventions** | **Naturalness** | **Animal experience** | **Food safety** | **Humane animal care** | **Other** |
| --- | --- | --- | --- | --- | --- |
| Enhanced hygiene and disease prevention measures |  |  |  |  |  |
| Using medicines and antibiotics to treat sick pigs |  |  |  |  |  |
| The preventive use of veterinary drugs, including antibiotics |  |  |  |  |  |
| Use of feed supplements e.g. probiotics |  |  |  |  |  |
| The use of vaccination |  |  |  |  |  |
| Efficient monitoring of pigs and pig house conditions |  |  |  |  |  |
| Enhanced control of air movement in pig houses |  |  |  |  |  |
| Improvements in pigs’ diet composition |  |  |  |  |  |
| Adjustments in the quantity of feed available |  |  |  |  |  |
| Breeding for genetically tougher or more resilient pigs |  |  |  |  |  |
| Improvements in housing design |  |  |  |  |  |
| Housing that protects the pigs from adverse natural conditions |  |  |  |  |  |
| Reducing the number of pigs in a given area |  |  |  |  |  |
| Providing enrichment materials so pigs can perform natural behaviors |  |  |  |  |  |
| Providing farmers with a price premium that encourages enhanced animal health |  |  |  |  |  |
| Doing nothing |  |  |  |  |  |

**15 How likely do you think it is that these interventions will be used. Please indicate your opinion below** Please tick one box in each row.

| **Interventions** | **Very unlikely** | **Unlikely** | **Neither likely nor unlikely** | **Likely** | **Very likely** |
| --- | --- | --- | --- | --- | --- |
| Enhanced hygiene and disease prevention measures |  |  |  |  |  |
| Using medicines and antibiotics to treat sick pigs |  |  |  |  |  |
| The preventive use of veterinary drugs, including antibiotics |  |  |  |  |  |
| Use of feed supplements e.g. probiotics |  |  |  |  |  |
| The use of vaccination |  |  |  |  |  |
| Efficient monitoring of pigs and pig house conditions |  |  |  |  |  |
| Enhanced control of air movement in pig houses |  |  |  |  |  |
| Improvements in pigs’ diet composition |  |  |  |  |  |
| Adjustments in the quantity of feed available |  |  |  |  |  |
| Breeding for genetically tougher or more resilient pigs |  |  |  |  |  |
| Improvements in housing design |  |  |  |  |  |
| Housing that protects the pigs from adverse natural conditions |  |  |  |  |  |
| Reducing the number of pigs in a given area |  |  |  |  |  |
| Providing enrichment materials so pigs can perform natural behaviors |  |  |  |  |  |
| Providing farmers with a price premium that encourages enhanced animal health |  |  |  |  |  |
| Doing nothing |  |  |  |  |  |

**16 Please indicate extent to which you agree or disagree with the following statements by ticking one box in each row.**

|  | **Strongly disagree** | **Disagree** | **Neither agree nor disagree** | **Agree** | **Strongly agree** |
| --- | --- | --- | --- | --- | --- |
| I purchase foods produced using intensive production systems |  |  |  |  |  |
| I intend to purchase foods produced using intensive production systems |  |  |  |  |  |
| I would consider purchasing foods produced by intensive production systems |  |  |  |  |  |
| I plan to reduce my consumption of foods from intensive production systems |  |  |  |  |  |
| I avoid purchasing foods from intensive production systems |  |  |  |  |  |
| I feel that I have an obligation to *purchase* animal products from intensive production systems |  |  |  |  |  |
| I feel that I have an ethical obligation to *avoid* animal products from intensive production systems |  |  |  |  |  |
| I think of myself as someone who is concerned about intensive animal production systems |  |  |  |  |  |
| I think of myself as someone who is concerned about farm animal welfare |  |  |  |  |  |

**17 Which actor(s) in the food chain do you think should bear the costs incurred as a result of any interventions introduced to prevent production diseases in intensive production systems?** Please tick one box in each row.

| **Stakeholder** | **Yes** | **No** |
| --- | --- | --- |
| Livestock farmers/ producers through making less profit |  |  |
| Food manufacturers through making less profit |  |  |
| Food retailers through making less profit |  |  |
| Consumers, through higher product prices |  |  |
| The general public, through increased taxes |  |  |
| National government through providing subsidies and funds |  |  |
| European Commission, through providing subsidies |  |  |

**18 Gender**

| Male |  |
| --- | --- |
| Female |  |
| I do not wish to specify |  |

**19 How old are you?**

|  |
| --- |

Years

**20 Which of the following best describe your highest education level attained.**

| Less than primary education |  |
| --- | --- |
| Primary education |  |
| Secondary education |  |
| Vocational education |  |
| University education |  |

**21 What is your yearly household income before tax?**

| < £8,000 |  |
| --- | --- |
| £8,001 - £16,000 |  |
| £16,001 - £32,000 |  |
| £32,001 -£40,000 |  |
| £40,001 -£64,000 |  |
| > £64,001 |  |
| I do not wish to specify |  |

**22 Which of the below describes your dietary choices?** Tick all that apply

| I eat pork |  |
| --- | --- |
| I eat poultry meat |  |
| I eat eggs |  |
| I eat meat and plants |  |
| I am vegetarian |  |
| I am vegan |  |
| I do not wish to specify |  |

**23 How many persons are there in your household?**

|  |
| --- |

**24 How many persons under 18 years old are there in your household?**

|  |
| --- |

**25 Are you the person who does the main food shopping in your household?**

| Yes |  |
| --- | --- |
| No |  |
| Joint responsibility |  |

**26 How would you describe where you live?**

| City centre |  |
| --- | --- |
| Town or suburb |  |
| Rural area |  |

**26 Which of the following best describes your religious beliefs?**

| Buddhist |  |
| --- | --- |
| Christian |  |
| Hindu |  |
| Jewish |  |
| Muslim |  |
| Sikh |  |
| Other |  |
| Atheist/ agnostic |  |
| I do not wish to specify |  |

**27 What is your employment status?** *Tick all that apply*

| Employed full-time |  |
| --- | --- |
| Employed part-time |  |
| Retired |  |
| Homemaker |  |
| Student |  |
| Unemployed |  |
| I do not wish to specify |  |

**Thank you for your valuable contribution to our project.** If you would like to find out more about the project and the research taking place, please visit our website, <http://www.fp7-prohealth.eu/>. Please remember that you are free to withdraw from this study at any time, without having to provide any underlying reason for doing so, and can so by contacting [prohealth@newcastle.ac.uk](mailto:prohealth@newcastle.ac.uk). If you would like to be entered into the prize draw please provide your contact details below.

## C: Additional data tables

***Table B Population characteristics for the broilers survey***

|  |  | Overall | Finland | | | Germany | | Poland | | Spain | | UK | |
| --- | --- | --- | --- | --- | --- | --- | --- | --- | --- | --- | --- | --- | --- |
|  | N | 789 | | 158 | 158 | | 157 | | 158 | | 158 | |  |
| Gender | Male | 49.2* | | 49.4 | 48.1 | | 50.3 | | 49.4 | | 48.7 | |  |
|  | Female | 50.4* | | 50.0 | 51.9 | | 49.7 | | 49.4 | | 51.3 | |  |
| Age | 18-24 years | 13.7 | | 11.4 | 13.3 | | 13.4 | | 16.5 | | 13.9 | |  |
|  | 25-34 years | 24.2 | | 25.9 | 20.3 | | 24.2 | | 29.7 | | 20.9 | |  |
|  | 35-44 years | 22.7 | | 25.9 | 19.6 | | 21.7 | | 24.7 | | 21.5 | |  |
|  | 45-54 years | 21.4 | | 19.0 | 26.6 | | 19.7 | | 18.4 | | 23.4 | |  |
|  | 55-64 years | 13.7 | | 12.7 | 17.7 | | 18.5 | | 9.5 | | 10.1 | |  |
|  | 65+ years | 4.4 | | 5.0 | 2.5 | | 2.5 | | 1.2 | | 10.1 | |  |
| Education | Less than primary | 0.4 | | 0.6 | 0.0 | | 0.6 | | 0.0 | | 0.0 | |  |
|  | Primary education | 1.0 | | 1.9 | 1.3 | | 0.6 | | 0.6 | | 0.6 | |  |
|  | Secondary education | 28.5 | | 23.4 | 27.2 | | 34.4 | | 20.3 | | 37.3 | |  |
|  | University education | 47.0 | | 43.0 | 24.1 | | 61.1 | | 65.2 | | 41.8 | |  |
|  | Vocational education | 23.1 | | 31.0 | 47.5 | | 3.2 | | 13.3 | | 20.3 | |  |
| Income | Less than 5,000 € | 2.5 | | 1.9 | 2.5 | | 2.5 | | 3.2 | | 2.5 | |  |
|  | 5 001 - 10 000 € | 8.6 | | 9.5 | 7.0 | | 17.2 | | 7.0 | | 2.5 | |  |
|  | 10 001 – 20 000 € | 19.8 | | 12.7 | 12.0 | | 32.5 | | 26.6 | | 15.2 | |  |
|  | 20 001 – 40 000 € | 30.4 | | 25.3 | 32.9 | | 31.8 | | 36.1 | | 25.9 | |  |
|  | 40 001 – 60 000 € | 14.6 | | 16.5 | 17.1 | | 2.5 | | 13.9 | | 22.8 | |  |
|  | 60 001 – 80 000 € | 7.5 | | 12.0 | 8.2 | | 0.6 | | 5.7 | | 10.8 | |  |
|  | More than 80 001 € | 5.2 | | 10.1 | 4.4 | | 0.0 | | 1.9 | | 9.5 | |  |
|  | I do not wish to specify | 11.4 | | 12.0 | 15.8 | | 12.7 | | 5.7 | | 10.8 | |  |
| Diet | Vegan | 1.5 | | 3.8 | 3.2 | | 0.0 | | 0.0 | | 0.6 | |  |
|  | Vegetarian | 6.5 | | 10.8 | 7.0 | | 5.1 | | 3.8 | | 5.7 | |  |
|  | Omnivore | 90.6 | | 85.4 | 88.0 | | 94.3 | | 95.6 | | 89.9 | |  |
|  | I do not wish to specify | 1.4 | | 0.0 | 1.9 | | 0.6 | | 0.6 | | 3.8 | |  |
| Place of residence | City centre | 35.6 | | 22.2 | 40.6 | | 40.4 | | 52.5 | | 22.2 | |  |
|  | Town or suburb | 48.7 | | 63.3 | 43.1 | | 44.9 | | 32.3 | | 60.1 | |  |
|  | Rural area | 15.7 | | 14.6 | 16.3 | | 14.7 | | 15.2 | | 17.7 | |  |

*values for gender to not add up to 100% due to 2 persons not wishing to specify their gender

***Table C Layer survey participant characteristics***

|  |  | Overall | Finland | Germany | Poland | Spain | UK |
| --- | --- | --- | --- | --- | --- | --- | --- |
|  | N | 790 | 158 | 160 | 156 | 158 | 158 |
| Gender | Male | 49.7 | 49.4 | 50.6 | 49.4 | 50.0 | 49.4 |
|  | Female | 49.9 | 50.0 | 48.8 | 50.6 | 49.4 | 50.6 |
| Age | 18-24 years | 13.3 | 11.4 | 13.8 | 13.5 | 15.2 | 12.7 |
|  | 25-24 years | 24.2 | 26.6 | 18.1 | 24.4 | 30.4 | 21.5 |
|  | 35-44 years | 22.3 | 21.5 | 21.3 | 21.8 | 25.3 | 21.5 |
|  | 45-54 years | 22.8 | 22.2 | 27.5 | 19.9 | 20.9 | 23.4 |
|  | 55-64 years | 12.4 | 11.4 | 18.8 | 16.7 | 6.3 | 8.9 |
|  | 65+ years | 5.1 | 6.9 | 0.6 | 3.8 | 1.9 | 12.0 |
| Education | Less than primary education | 0.3 | 0.0 | 0.6 | 0.0 | 0.0 | 0.6 |
|  | Primary education | 1.5 | 4.4 | 1.3 | 0.0 | 1.9 | 0.0 |
|  | Secondary education | 30.1 | 25.3 | 30.6 | 35.3 | 22.2 | 37.3 |
|  | University education | 46.6 | 39.2 | 36.9 | 59.6 | 54.4 | 43.0 |
|  | Vocational education | 21.5 | 31.0 | 30.6 | 5.1 | 21.5 | 19.0 |
| Income | Less than 5,000 € | 8.6 | 10.8 | 6.3 | 16.7 | 7.0 | 2.5 |
|  | 5 001 - 10 000 € | 3.5 | 2.5 | 0.6 | 5.8 | 1.9 | 7.0 |
|  | 10 001 – 20 000 € | 19.5 | 19.0 | 10.6 | 34.0 | 19.0 | 15.2 |
|  | 20 001 – 40 000 € | 30.4 | 27.8 | 33.1 | 24.4 | 41.8 | 24.7 |
|  | 40 001 – 60 000 € | 15.1 | 13.3 | 19.4 | 2.6 | 15.8 | 24.1 |
|  | 60 001 – 80 000 € | 7.5 | 8.2 | 13.1 | 1.9 | 7.0 | 7.0 |
|  | More than 80 001 € | 5.6 | 5.7 | 5.6 | 0.6 | 0.6 | 15.2 |
|  | I do not wish to specify | 9.9 | 12.7 | 11.3 | 14.1 | 7.0 | 4.4 |
| Diet | Vegan | 0.9 | 0.0 | 1.9 | 1.3 | 0.0 | 1.3 |
|  | Vegetarian | 6.1 | 6.3 | 8.8 | 4.5 | 3.2 | 7.6 |
|  | Omnivore | 92.2 | 93.0 | 88.1 | 94.2 | 95.6 | 89.9 |
|  | I do not wish to specify | 0.9 | 0.6 | 1.3 | 0.0 | 1.3 | 1.3 |
| Place of residence | City centre | 35.9 | 24.1 | 31.6 | 38.2 | 62.0 | 23.4 |
|  | Town or suburb | 46.6 | 60.1 | 36.1 | 49.7 | 29.1 | 58.2 |
|  | Rural area | 17.5 | 15.8 | 32.2 | 12.1 | 8.9 | 18.4 |

*values for gender to not add up to 100% due to 3 persons not wishing to specify their gender

***Table D Pigs survey participant characteristics***

|  |  | Overall | Finland | Germany | Poland | Spain | UK |
| --- | --- | --- | --- | --- | --- | --- | --- |
|  | N | 751 | 150 | 150 | 151 | 152 | 148 |
| Gender | Male | 48* | 49.3* | 51 | 48.7 | 50 | 49.4* |
|  | Female | 50.3* | 50.7* | 50.7 | 49 | 51.3 | 50 |
| Age | 18-24 years | 13.6 | 10.7 | 12.7 | 15.2 | 16.4 | 12.8 |
|  | 25-34 years | 23.6 | 24.7 | 18.7 | 23.8 | 30.3 | 20.9 |
|  | 35-44 years | 22.9 | 26.7 | 19.3 | 22.5 | 23.7 | 22.3 |
|  | 45-54 years | 22.5 | 19.3 | 29.3 | 19.9 | 20.4 | 23.6 |
|  | 55-64 years | 13.6 | 12.7 | 20 | 13.9 | 7.9 | 13.5 |
|  | 65+ years | 3.8 | 6 | 0.7 | 4.7 | 1.4 | 6.8 |
| Education | Less than primary education | 0.1 | 0 | 0.7 | 0 | 0 | 0 |
|  | Primary education | 1.7 | 3.3 | 1.3 | 0.7 | 0.7 | 2.7 |
|  | Secondary education | 26 | 26.7 | 24 | 29.1 | 20.4 | 29.7 |
|  | University education | 49.7 | 33.3 | 39.3 | 64.9 | 63.8 | 46.6 |
|  | Vocational education | 22.5 | 36.7 | 34.7 | 5.3 | 15.1 | 20.9 |
| Income | Less than 5,000 € | 3.6 | 6 | 1.3 | 6.6 | 2 | 2 |
|  | 5 001 - 10 000 € | 8.3 | 7.3 | 6 | 17.9 | 7.9 | 2 |
|  | 10 001 – 20 000 € | 19.7 | 12 | 12.9 | 29.9 | 25.3 | 18.4 |
|  | 20 001 – 40 000 € | 29.6 | 28 | 22 | 30.5 | 38.8 | 28.4 |
|  | 40 001 – 60 000 € | 14.9 | 12.7 | 24 | 3.3 | 15.1 | 19.6 |
|  | 60 001 – 80 000 € | 7.3 | 10.7 | 14.7 | 0.7 | 2.6 | 8.1 |
|  | More than 80 001 € | 6.7 | 9.3 | 10 | 0.7 | 1.3 | 12.2 |
|  | I do not wish to specify | 9.6 | 14 | 8.7 | 9.9 | 6.6 | 8.8 |
| Diet | Vegan | 0.9 | 0 | 0.7 | 1.3 | 0 | 2.7 |
|  | Vegetarian | 5.9 | 6.7 | 10.7 | 2.6 | 2.6 | 6.8 |
|  | Omnivore | 92.8 | 91.3 | 88.7 | 96 | 97.4 | 90.5 |
|  | I do not wish to specify | 0.4 | 2 | 0 | 0 | 0 | 0 |
| Place of residence | City centre | 37.3 | 24.1 | 35.3 | 37.7 | 61.8 | 26.4 |
|  | Town or suburb | 45.4 | 58 | 38.7 | 46.4 | 30.3 | 54.1 |
|  | Rural area | 17.3 | 17.3 | 26 | 15.9 | 7.9 | 19.6 |

*values for gender to not add up to 100% due to 3 persons not wishing to specify their gender

***Table E Proportion (%) of respondents who reported not being connected to raising farm animals, nor having visited a farm and nor having heard about production diseases from various sources if information.***

| Unfamiliarity | Layers  (n=790) | Broilers  (n=789) | Pigs  (n=751) |
| --- | --- | --- | --- |
| Q1: Connections to raising farm animals | 74.1% | 78.6% | 77.1% |
| Q2: Have you visited a working farm? | 78.4% | 83.3% | 77.5% |
| Have you heard about production diseases from; | | | |
| Q3: Television | 57.3% | 51.6% | 58.1% |
| Q3: Radio | 81.0% | 79.6% | 82.8% |
| Q3: Newspaper | 73.2% | 71.7% | 74.7% |
| Q3: Magazine | 87.5% | 85.7% | 88.5% |
| Q3: The internet | 64.2% | 62.1% | 66.7% |
| Q3: Social media | 82.4% | 75.7% | 80.7% |
| Q3: Friends and family | 75.6% | 74.7% | 79.2% |

***Table*** ***F Attitude and purchase intentions towards intensive broiler chicken production systems***

| Intensive production systems are … | Overall | Finland | Germany | Poland | Spain | UK | Chi square |
| --- | --- | --- | --- | --- | --- | --- | --- |
| n | 789 | 158 | 158 | 157 | 158 | 158 |  |
| Unpleasant (1)/pleasant (5) | 2.09 ± 1.03 | 2.33 ± 1.04 | 1.83 ± 1.00 | 2.05 ± 0.97 | 2.21 ± 1.11 | 2.11 ± 1.11 | ** |
| Bad (1)/good (5) | 2.32 ± 1.05 | 2.44 ± 1.07 | 1.84 ± 0.94 | 2.34 ± 0.96 | 2.59 ± 1.04 | 2.38 ± 1.10 | *** |
| Worthless (1)/valuable (5) | 2.72 ± 1.05 | 2.78 ± 1.02 | 2.35 ± 1.09 | 2.75 ± 1.02 | 2.67 ± 1.00 | 3.03 ± 0.99 | *** |
| Useless (1)/useful (5) | 3.06 ± 1.11 | 3.22 ± 1.10 | 2.55 ± 1.16 | 3.04 ± 1.10 | 3.40 ± 1.02 | 3.10 ± 1.00 | *** |
| Unsafe (1)/safe (5) | 2.63 ± 1.08 | 2.66 ± 1.14 | 2.34 ± 1.05 | 2.53 ± 0.98 | 2.85 ± 1.00 | 2.78 ± 1.13 | *** |
| Unethical (1)/ethical (5) | 2.12 ± 1.06 | 2.21 ± 1.12 | 1.88 ± 1.06 | 2.00 ± 0.90 | 2.30 ± 1.05 | 2.23 ± 1.12 | *** |

All responses are reported as the mean ± the standard deviation (SD). Responses are based on a 1 to 5 Likert scale, with some scale items revered from their wording in the survey to provide consistency in analysis. Significance values for Chi-square test to indicate between country differences: *p< 0.05, ** p< 0.01 and *** p<0.001

***Table G Attitude and purchase intentions towards intensive layer hen production systems***

| Intensive production systems are … | Overall | Finland | Germany | Poland | Spain | UK | Chi-square |
| --- | --- | --- | --- | --- | --- | --- | --- |
| n | 790 | 158 | 160 | 156 | 158 | 158 |  |
| Unpleasant (1)/pleasant (5) | 2.22 ± 1.04 | 2.41 ± 0.93 | 1.93 ± 1.03 | 2.35 ± 1.02 | 2.28 ± 0.97 | 2.15 ± 1.17 | *** |
| Bad (1)/good (5) | 2.44 ± 1.05 | 2.72 ± 0.95 | 1.92 ± 0.97 | 2.63 ± 1.04 | 2.59 ± 1.00 | 2.34 ± 1.12 | *** |
| Worthless (1)/valuable (5) | 3.06 ± 1.08 | 3.03 ± 0.92 | 2.67 ± 1.14 | 3.11 ± 1.02 | 3.13 ± 1.01 | 3.15 ± .13 | *** |
| Useless (1)/useful (5) | 3.06 ± 1.08 | 3.23 ± 0.97 | 2.67 ± 1.14 | 3.11 ± 1.03 | 3.13 ± 1.01 | 3.15 ± 1.13 | *** |
| Unsafe (1)/safe (5) | 2.64 ± 1.07 | 2.72 ± 1.01 | 2.33 ± 1.10 | 2.62 ± 1.00 | 2.80 ± 1.04 | 2.70 ± 1.13 | ** |
| Unethical (1)/ethical (5) | 2.17 ± 1.04 | 2.35 ± 0.96 | 1.81 ± 0.99 | 2.33 ± 0.97 | 2.18 ± 0.99 | 2.18 ± 1.17 | *** |

All responses are reported as the mean ± the standard deviation (SD). Responses are based on a 1 to 5 Likert scale, with some scale items revered from their wording in the survey to provide consistency in analysis... Significance values for Chi-square test to indicate between country differences: *p< 0.05, ** p< 0.01 and *** p<0.001

***Table H Attitude and purchase intentions towards intensive pig production systems***

| Intensive production systems are … | Overall | Finland | Germany | Poland | Spain | UK | Chi-square |
| --- | --- | --- | --- | --- | --- | --- | --- |
| n | 751 | 150 | 150 | 151 | 152 | 148 |  |
| Unpleasant (1)/pleasant (5) | 2.18 ± 1.06 | 2.16 ± 0.95 | 1.93 ± 1.16 | 2.28 ± 1.00 | 2.26 ± 0.99 | 2.24 ± 1.17 | ** |
| Bad (1)/good (5) | 2.43 ± 1.04 | 2.52 ± 1.03 | 1.95 ± 1.05 | 2.54 ± 0.94 | 2.66 ± 0.91 | 2.48 ± 1.11 | *** |
| Worthless (1)/valuable (5) | 2.76 ± 1.03 | 2.81 ± 0.93 | 2.41 ± 1.12 | 2.77 ± 0.93 | 2.60 ± 0.98 | 3.21 ± 1.02 | *** |
| Useless (1)/useful (5) | 3.09 ± 1.06 | 3.14 ± 1.02 | 2.63 ± 1.16 | 3.25 ± 0.98 | 3.28 ± 0.99 | 3.16 ± 1.00 | *** |
| Unsafe (1)/safe (5) | 2.65 ± 1.08 | 2.59 ± 1.11 | 2.27 ± 1.09 | 2.70 ± 0.96 | 2.86 ± 1.05 | 2.84 ± 1.10 | *** |
| Unethical (1)/ethical (5) | 2.23 ± 1.10 | 2.19 ± 1.07 | 2.87 ± 1.05 | 2.34 ± 1.03 | 2.45 ± 1.06 | 2.32 ± 1.20 | *** |

All responses are reported as the mean ± the standard deviation (SD). Responses are based on a 1 to 5 Likert scale, with some scale items revered from their wording in the survey to provide consistency in analysis.. Significance values for Chi-square test to indicate between country differences: *p< 0.05, ** p< 0.01 and *** p<0.001

| Behavioural intention | Layers  (n=790)) | Broilers  (n=789) | Pigs  (n=751) |
| --- | --- | --- | --- |
| I purchase foods produced using intensive production systems | 3.00 ± 1.06* | 3.04 ± 1.06** | 3.01 ± 1.02* |
| I intend to purchase foods produced using intensive production systems | 2.84 ± 1.12** | 2.79 ± 1.08** | 2.79 ± 1.06 |
| I would consider purchasing foods produced by intensive production systems | 2.91 ± 1.11 | 2.90 ± 1.07** | 2.94 ± 1.06* |
| I plan to reduce my consumption of foods from intensive production systems | 3.43 ± 1.02* | 3.53 ± 1.02 | 3.42 ± 1.07* |
| I avoid purchasing foods from intensive production systems | 3.38 ± 1.05* | 3.36 ± 1.09 | 3.34 ± 1.07 |
| I feel that I have an obligation to purchase animal products from intensive production systems | 2.62 ± 1.16*** | 2.56 ± 1.15** | 2.59 ± 1.13*** |
| I feel that I have an ethical obligation to avoid animal products from intensive production systems | 3.53 ± 1.11*** | 3.57 ± 1.05 | 3.53 ± 1.10** |
| I think of myself as someone who is concerned about intensive animal production systems | 3.53 ± 1.00*** | 3.57 ± 1.01* | 3.53 ± 1.03** |
| I think of myself as someone who is concerned about farm animal welfare | 3.70 ± 0.97* | 3.67 ± 1.01 | 3.67 ± 0.99* |

***Table I Overall behavioural and purchase intentions of respondents in five countries towards food originating from intensive animal production systems involving laying hens, broiler chickens and pigs (mean response on a linear scale 1 (Strongly disagree) to 5 (Strongly agree))***

Significance values for Chi-square test to indicate between country differences: *p< 0.05, ** p< 0.01 and *** p<0.001

***Table J The respondents’ views in five countries regarding overall trust in information provision and responsibility for information provision and animal health and welfare of laying hens, broiler chickens and pigs (mean response on a linear scale 1 to 5 ± SD)***

|  | Layer (n=790) | | | | | | | Broilers (n=789) | | | | | | | Pigs (n=751) | | | | | | |
| --- | --- | --- | --- | --- | --- | --- | --- | --- | --- | --- | --- | --- | --- | --- | --- | --- | --- | --- | --- | --- | --- |
| Stakeholder | **Responsible health** | | **Responsible welfare** | | **Trusted information** | | **Responsible health** | | | **Responsible welfare** | | | **Trusted information** | | **Responsible health** | | **Responsible welfare** | | | **Trusted information** | |
| Animal welfare organisations | 3.91 ± 1.08 | | 4.11 ± 0.87 | | 3.93 ± 0.97 | | 3.89 ± 1.03 | | | 4.09 ± 0.95 | | | 3.81 ± 1.03 | | 3.93 ± 1.00 | | 4.06 ± 1.02 | | | 3.78 ± 1.04 | |
| European Commission | 3.91 ± 1.02 | | 3.91 ± 1.02 | | 3.39 ± 1.03 | | 3.96 ± 0.96 | | | 3.97 ± 0.99 | | | 3.36 ± 1.06 | | 3.87 ± 1.06 | | 3.90 ± 1.05 | | | 3.34 ± 1.06 | |
| National government | 3.99 ± 1.04 | | 4.02 ± 0.98 | | 3.27 ± 1.10 | | 4.06 ± 0.94 | | | 4.07 ± 0.93 | | | 3.26 ± 1.10 | | 4.01 ± 0.99 | | 4.00 ± 1.01 | | | 3.25 ± 1.09 | |
| Veterinarians | 4.12 ± 0.98 | | 4.09 ± 0.96 | | 3.79 ± 0.96 | | 4.11 ± 0.94 | | | 4.16 ± 0.89 | | | 3.71 ± 0.97 | | 4.23 ± 0.90 | | 4.23 ± 0.90 | | | 3.79 ± 0.92 | |
| Animal health authorities | 4.33 ± 0.85 | | 4.28 ± 0.86 | | 3.76 ± 0.96 | | 4.34 ± 0.86 | | | 4.31 ± 0.84 | | | 3.74 ± 1.01 | | 4.33 ± 0.90 | | 4.28 ± 0.89 | | | 3.80 ± 0.95 | |
| Veterinary medicine producers | 3.96 ± 0.89 | | 3.94 ± 1.00 | | 3.23 ± 1.10 | | 3.99 ± 0.78 | | | 3.93 ± 1.00 | | | 3.20 ± 1.09 | | 4.05 ± 0.97 | | 3.97 ± 1.01 | | | 3.22 ± 1.06 | |
| Animal breeding companies | 4.28 ± 0.92 | | 4.27 ± 0.89 | | 3.12 ± 1.16 | | 4.35 ± 0.89 | | | 4.31 ± 0.87 | | | 3.01 ± 1.18 | | 4.31 ± 0.91 | | 4.29 ± 0.89 | | | 3.14 ± 1.14 | |
| Animal feed producers | 4.04 ± 1.01 | | 3.92 ± 1.01 | | 3.09 ± 1.10 | | 4.02 ± 0.99 | | | 3.95 ± 1.00 | | | 2.96 ± 1.09 | | 4.11 ± 0.97 | | 4.00 ± 1.00 | | | 3.07 ± 1.06 | |
| Animal housing manufacturers | 3.96 ± 0.98 | | 4.00 ± 0.97 | | 3.05 ± 1.06 | | 3.81 ± 1.05 | | | 3.96 ± 1.01 | | | 2.98 ± 1.05 | | 3.81 ± 1.05 | | 3.96 ± 1.01 | | | 3.07 ± 1.06 | |
| Farmers | 4.39 ± 0.86 | | 4.39 ± 0.85 | | 3.27 ± 1.13 | | 4.43 ± 0.85 | | | 4.44 ± 0.82 | | | 3.19 ± 1.12 | | 4.46 ± 0.82 | | 4.42 ± 0.84 | | | 3.33 ± 1.11 | |
| Animal transporters | 3.96 ± 0.98 | | 3.96 ± 1.02 | | 2.96 ± 1.09 | | 4.08 ± 0.97 | | | 4.04 ± 1.00 | | | 2.89 ± 1.07 | | 3.98 ± 1.00 | | 4.10 ± 1.00 | | | 2.91 ± 1.05 | |
| Slaughterhouses | 3.95 ± 1.08 | | 3.96 ± 1.02 | | 2.98 ± 1.11 | | 4.05 ± 1.02 | | | 4.04 ± 1.03 | | | 2.95 ± 1.13 | | 4.09 ± 1.04 | | 4.05 ± 1.06 | | | 3.01 ± 1.12 | |
| Quality assurance systems | 4.16 ± 0.92 | 4.10 ± 0.93 | | 3.61 ± 0.99 | | 4.17 ± 0.92 | | | 4.14 ± 0.92 | | 3.59 ± 1.02 | | | 4.15 ± 0.93 | | 4.09 ± 0.96 | | | 3.63 ± 0.95 | |  |
| Food manufacturers | 4.07 ± 0.94 | 3.92 ± 1.00 | | 3.09 ± 1.11 | | 4.13 ± 0.94 | | | 3.97 ± 0.99 | | 3.02 ± 1.10 | | | 4.04 ± 1.00 | | 3.91 ± 1.04 | | | 3.05 ± 1.07 | |  |
| Food retailers | 3.71 ± 1.00 | 3.67 ± 1.01 | | 3.08 ± 1.05 | | 3.76 ± 0.99 | | | 3.77 ± 1.01 | | 3.02 ± 1.05 | | | 3.72 ± 1.03 | | 3.68 ± 1.07 | | | 3.08 ± 1.01 | |  |
| Consumer organisations | 3.77 ± 0.97 | 3.77 ± 1.00 | | 3.69 ± 0.93 | | 3.76 ± 1.02 | | | 3.81 ± 1.00 | | 3.66 ± 0.95 | | | 3.75 ± 1.01 | | 3.74 ± 1.03 | | | 3.63 ± 0.92 | |  |
| The general public | 3.39 ± 1.09 | 2.46 ± 1.11 | |  | | 3.41 ± 1.09 | | | 3.50 ± 1.08 | |  | | | 3.39 ± 1.10 | | 3.50 ± 1.11 | | |  | |  |
| You as a consumer | 3.38 ± 1.14 | 3.48 ± 1.11 | |  | | 3.37 ± 1.16 | | | 3.45 ± 1.11 | |  | | | 3.34 ± 1.19 | | 3.42 ± 1.13 | | |  | |  |
| Social media |  |  | | 3.01 ± 1.12 | |  | | |  | | 2.98 ± 1.10 | | |  | |  | | | 2.94 ± 1.13 | |  |
| Traditional media |  |  | | 3.40 ± 0.96 | | | |  |  | | | 3.36 ± 0.97 | |  | | | |  | 3.33 ± 1.02 | |  |

|  | Layers (n=790) | | | Broilers (n-890) | | | Pigs (n=751) | | |
| --- | --- | --- | --- | --- | --- | --- | --- | --- | --- |
|  | **General concern** | **Human concern** | **Animal concern** | **General concern** | **Human concern** | **Animal concern** | **General concern** | **Human concern** | **Animal concern** |
| The current minimum animal welfare standards associated with X production | 3.78 ± 0.97** | 3.50 ± 1.05*** | 3.91 ± 0.91*** | 3.83 ± 0.98** | 3.56 ± 0.99*** | 3.77 ± 0.99** | 3.77 ± 0.97*** | 3.54 ± 1.01*** | 3.75 ± 1.00*** |
| Whether animal welfare standards are actually achieved in X production | 3.83 ± 0.97** | 3.66 ± 0.98 | 3.82 ± 0.96*** | 3.88 ± 1.01 | 3.70 ± 0.99** | 3.84 ± 0.98* | 3.85 ± 0.99*** | 3.68 ± 0.97*** | 3.83 ± 0.96*** |
| Use of antibiotics in production animals as a growth promoter | 3.95 ± 1.09 | 4.03 ± 0.99 | 3.92 ± 0.99*** | 4.04 ± 1.07 | 4.05 ± 0.99** | 3.95 ± 0.98*** | 3.98 ± 1.07** | 4.03 ± 0.97*** | 3.93 ± 1.01*** |
| Use of antibiotics in production animals to prevent diseases | 3.82 ± 1.06** | 3.89 ± 1.01 | 3.75 ± 1.04 | 3.89 ± 1.06** | 3.96 ± 0.96*** | 3.72 ± 1.05*** | 3.86 ± 1.05*** | 3.84 ± 1.01*** | 3.69 ± 1.05*** |
| Use of antibiotics in production animals to treat diseases | 3.67 ± 1.10*** | 3.77 ± 1.02*** | 3.56 ± 1.13* | 3.71 ± 1.11*** | 3.78 ± 1.08*** | 3.54 ± 1.12*** | 3.63 ± 1.12*** | 3.70 ± 1.04*** | 3.49 ± 1.13*** |
| Use of vaccinations to prevent animal diseases | 3.43 ± 1.14*** | 3.50 ± 1.08* | 3.36 ± 1.13* | 3.43 ± 1.13*** | 3.49 ± 1.10* | 3.31 ± 1.16 | 3.43 ± 1.13*** | 3.47 ± 1.08*** | 3.31 ± 1.17*** |
| Use of other veterinary medicines to treat animal diseases | 3.43 ± 1.14** | 3.54 ± 1.02*** | 3.40 ± 1.10* | 3.43 ± 1.10*** | 3.53 ± 1.04*** | 3.38 ± 1.12* | 3.42 ± 1.12*** | 2.51 ± 1.04*** | 3.33 ± 1.10*** |
| Use of probiotics to prevent animal diseases | 3.40 ± 1.11*** | 3.45 ± 1.08*** | 3.36 ± 1.10*** | 3.43 ± 1.15*** | 3.47 ± 1.14*** | 3.40 ± 1.14*** | 3.38 ± 1.12*** | 3.46 ± 1.07*** | 3.37 ± 1.14*** |
| Antibiotic residues in food* | 4.00 ± 1.07* | 4.04 ± 0.97* | 3.93 ± 0.95* | 4.05 ± 1.06** | 4.08 ± 0.98*** | 3.89 ± 1.01*** | 3.98 ± 1.02*** | 4.02 ± 1.00*** | 3.85 ± 1.01*** |
| Antibiotic resistance as a result of the use of antibiotics in animals | 4.10 ± 0.97 | 4.06 ± 0.96* | 3.99 ± 0.97* | 4.12 ± 0.96** | 4.09 ± 0.97*** | 3.98 ± 0.95*** | 4.08 ± 0.97*** | 4.04 ± 0.93*** | 3.93 ± 0.98*** |
| Animal production diseases in general | 3.96 ± 0.92 | 3.91 ± 0.91 | 3.97 ± 0.92** | 3.98 ± 0.93 | 3.94 ± 0.94* | 3.95 ± 0.94*** | 3.97 ± 0.93 | 3.94 ± 0.90*** | 3.92 ± 0.93*** |
| Impacts of animal diseases on food quality | 4.03 ± 0.92* | 3.93 ± 0.91** |  | 4.20 ± 0.95** | 3.99 ± 0.94** |  | 3.99 ± 0.96** | 3.95 ± 0.95*** |  |
| Impacts of animal diseases on food safety | 4.03 ± 0.92 | 3.98 ± 0.93 |  | 4.05 ± 0.96 | 4.02 ± 0.93*** |  | 4.02 ± 0.95* | 3.96 ± 0.94*** |  |
| Impacts of animal diseases on human health | 4.01 ± 0.94 |  |  | 4.03 ± 1.00*** |  |  | 4.01 ± 0.97*** |  |  |
| Impacts of animal diseases on animal welfare | 3.96 ± 0.81** |  |  | 3.95 ± 0.78* |  |  | 3.97 ± 0.93*** |  |  |
| Impact of animal diseases on the environment | 3.83 ± 0.96 |  |  | 3.86 ± 0.97*** |  |  | 3.83 ± 0.98*** |  |  |

***Table K The respondents’ concern regarding intensive animal production of laying hens, broiler chickens and pigs in general and in relation to human and animal health (mean response on a linear scale 1 to 5 ± SD)***

Significance values for Chi-square test to indicate between country differences: *p< 0.05, ** p< 0.01 and *** p<0.001

***Table L Intervention acceptability for broilers in intensive production systems among respondents in five countries (mean response on a linear scale 1 (Strongly disagree) to 5 (Strongly agree) ± SD)***

| To what extent do you agree or disagree that these are acceptable? | Overall | Finland | Germany | Poland | Spain | UK | Chi square |
| --- | --- | --- | --- | --- | --- | --- | --- |
| n | 789 | 158 | 158 | 157 | 158 | 158 |  |
| Adjustments in the quantity of feed available | 3.45 ± 1.01 | 3.36 ± 0.96 | 3.42 ± 1.05 | 3.31 ± 1.06 | 3.62 ± 0.98 | 3.53 ± 0.98 |  |
| Adjustments to feed composition | 3.63 ± 0.97 | 3.72 ± 0.90 | 3.68 ± 1.02 | 3.34 ± 1.07 | 3.83 ± 0.91 | 3.58 ± 0.89 | *** |
| Changes in the amount and time of light provision | 3.56 ± 1.01 | 3.67 ± 0.91 | 3.46 ± 1.05 | 3.48 ± 1.01 | 3.70 ± 1.04 | 3.49 ± 1.00 |  |
| Enhanced control air movement in chicken houses | 3.94 ± 0.94 | 4.13 ± 0.87 | 3.91 ± 0.97 | 3.94 ± 0.90 | 4.01 ± 0.93 | 3.72 ± 0.98 | ** |
| Enhanced maintenance of the quality of the bedding | 4.04 ± 0.88 | 4.20 ± 0.86 | 4.03 ± 0.94 | 4.04 ± 0.81 | 3.92 ± 0.87 | 3.72 ± 0.98 |  |
| Enhanced hygiene and disease prevention measures | 4.19 ± 0.91 | 4.34 ± 0.80 | 4.23 ± 0.60 | 4.07 ± 0.98 | 4.28 ± 0.82 | 4.00 ± 0.95 | ** |
| Housing that allows birds greater freedom to move | 4.18 ± 0.98 | 4.29 ± 0.88 | 4.16 ± 1.11 | 4.03 ± 1.01 | 4.16 ± 0.95 | 4.23 ± 0.90 |  |
| The preventative use of veterinary drugs | 2.88 ± 1.17 | 2.50 ± 1.05 | 2.50 ± 1.19 | 2.83 ± 1.12 | 3.35 ± 1.08 | 3.20 ± 1.13 | *** |
| Use of feed supplements e.g. probiotics | 3.12 ± 1.09 | 3.19 ± 1.07 | 2.74 ± 1.19 | 3.25 ± 0.96 | 3.13 ± 1.13 | 3.30 ± 1.02 | *** |
| Providing farmers with a price premium that encourages enhanced bird health | 3.94 ± 0.93 | 3.99 ± 0.98 | 4.03 ± 0.99 | 3.83 ± 0.94 | 3.94 ± 0.92 | 3.91 ± 0.83 |  |
| Providing materials and an environment where birds can perform natural behaviours | 4.15 ± 0.92 | 4.29 ± 0.87 | 4.24 ± 0.93 | 3.97 ± 0.98 | 4.11 ± 0.90 | 4.11 ± 0.91 | * |
| Improvements in housing design | 4.10 ± 0.89 | 4.27 ± 0.83 | 4.14 ± 0.92 | 3.91 ± 0.94 | 4.01 ± 0.87 | 4.18 ± 0.86 | ** |
| Housing that protects the birds from adverse natural conditions | 4.00 ± 0.90 | 4.04 ± 0.88 | 3.91 ± 0.94 | 3.96 ± 0.88 | 4.06 ± 0.91 | 4.05 ± 0.89 |  |
| Reducing the number of birds in a given area | 4.12 ± 0.90 | 4.18 ± 0.86 | 4.20 ± 0.95 | 4.05 ± 0.92 | 3.96 ± 0.90 | 4.20 ± 0.86 | * |
| Using antibiotics and medicines to treat sick birds | 3.33 ± 1.05 | 3.40 ± 0.98 | 3.13 ± 1.18 | 3.45 ± 1.02 | 3.46 ± 1.07 | 3.54 ± 0.94 | ** |
| The use of vaccination | 3.41 ± 0.99 | 3.37 ± 0.95 | 3.47 ± 1.12 | 3.14 ± 0.95 | 3.59 ± 0.99 | 3.49 ± 0.89 | *** |
| Doing nothing | 2.12 ± 1.19 | 2.10 ± 1.08 | 1.92 ± 1.18 | 2.25 ± 1.25 | 2.10 ± 1.30 | 2.21 ± 1.15 |  |

Significance values for Chi-square test to indicate between country differences: **p< 0.05, ** p< 0.01 and *** p<0.001*

| To what extent do you agree or disagree that these are acceptable? | Overall | Finland | Germany | Poland | Spain | UK | Chi square |
| --- | --- | --- | --- | --- | --- | --- | --- |
| n | 790 | 158 | 160 | 156 | 158 | 158 |  |
| Adjustments to the quantity of feed available | 3.46 ± 0.96 | 3.40 ± 0.92 | 3.44 ± 0.99 | 3.32 ± 0.96 | 3.64 ± 0.93 | 3.48 ± 0.96 |  |
| Adjustments to feed composition | 3.65 ± 0.95 | 3.61 ± 1.02 | 3.69 ± 0.92 | 3.33 ± 1.01 | 3.93 ± 0.91 | 3.72 ± 0.81 | *** |
| Changes in the amount and time of light provision | 3.59 ± 1.02 | 3.65 ± 0.96 | 3.52 ± 1.11 | 3.53 ± 0.98 | 3.61 ± 1.05 | 3.66 ± 0.98 |  |
| Enhanced control of air movement in chicken houses | 3.92 ± 0.92 | 4.13 ± 0.85 | 3.87 ± 0.98 | 3.91 ± 0.88 | 3.90 ± 0.93 | 3.79 ± 0.93 | ** |
| Enhanced maintenance of the quality of the bedding | 4.06 ± 0.88 | 4.23 ± 0.83 | 3.95 ± 0.92 | 4.07 ± 0.84 | 3.99 ± 0.94 | 4.09 ± 0.85 |  |
| Enhanced hygiene and disease prevention measures | 4.18 ± 0.86 | 4.43 ± 0.76 | 4.10 ± 0.95 | 4.10 ± 0.83 | 4.13 ± 0.92 | 4.14 ± 0.82 | *** |
| Housing that allows birds greater freedom to move | 4.11 ± 0.99 | 4.38 ± 0.85 | 4.04 ± 1.25 | 3.87 ± 0.86 | 4.06 ± 0.96 | 4.20 ± 0.90 | *** |
| The preventative use of veterinary drugs including antibiotics | 3.17 ± 1.21 | 2.56 ± 1.12 | 2.51 ± 1.14 | 4.07 ± 0.94 | 3.47 ± 1.03 | 3.28 ± 1.08 | *** |
| Use of feed supplements e.g. probiotics | 3.19 ± 1.07 | 3.11 ± 1.00 | 2.81 ± 1.12 | 3.31 ± 1.06 | 3.34 ± 1.11 | 3.40 ± 0.99 | *** |
| Providing farmers with a price premium that encourages enhanced bird health | 3.91 ± 0.96 | 3.90 ± 1.04 | 3.92 ± 1.07 | 3.91 ± 0.88 | 3.87 ± 0.95 | 3.92 ± 0.87 |  |
| Providing materials and an environment where birds can perform natural behaviours | 4.16 ± 0.92 | 4.34 ± 0.87 | 4.23 ± 1.00 | 4.02 ± 0.93 | 4.04 ± 0.97 | 4.18 ± 0.80 | ** |
| Improvements in housing design | 4.07 ± 0.92 | 4.27 ± 0.80 | 3.96 ± 1.04 | 4.02 ± 0.93 | 3.97 ± 0.96 | 4.11 ± 0.79 | * |
| Housing that protects the birds from adverse natural conditions | 3.99± 0.90 | 4.07 ± 0.85 | 3.86 ± 0.99 | 3.93 ± 0.97 | 4.13 ± 0.91 | 3.99 ± 0.86 |  |
| Reducing the number of birds in a given area | 4.10 ± 0.94 | 4.10 ± 0.87 | 4.23 ± 0.97 | 4.06 ± 0.97 | 3.91 ± 0.98 | 4.19 ± 0.86 | ** |
| Using antibiotics to treat sick birds | 3.34 ± 1.06 | 3.28 ± 1.00 | 3.22 ± 1.15 | 3.21 ± 0.99 | 3.49 ± 1.10 | 3.47 ± 1.05 | * |
| The use of vaccination | 3.45 ± 0.98 | 3.31 ± 0.96 | 3.49 ± 0.98 | 3.37 ± 0.94 | 3.49 ± 1.03 | 3.58 ± 0.96 |  |
| Doing nothing | 2.12 ± 1.21 | 1.94 ± 1.13 | 2.00 ± 1.19 | 2.28 ± 1.22 | 2.18 ± 1.28 | 2.18 ± 1.20 | * |

***Table M Intervention acceptability for layers in intensive production systems among respondents in five countries (mean response on a linear scale 1 (Strongly disagree) to 5 (Strongly agree) ± SD)***

Significance values for Chi-square test to indicate between country differences: **p< 0.05, ** p< 0.01 and *** p<0.001*

| To what extent do you agree or disagree that these are acceptable? | Overall | Finland | Germany | Poland | Spain | UK | Chi square |
| --- | --- | --- | --- | --- | --- | --- | --- |
| n | 751 | 150 | 150 | 151 | 152 | 148 |  |
| Enhanced hygiene and disease prevention measures | 4.16 ± 0.87 | 4.33 ± 0.77 | 4.29 ± 0.84 | 3.96 ± 0.94 | 4.16 ± 0.89 | 4.09 ± 0.85 | *** |
| Using medicines and antibiotics to treat sick picks | 3.37 ± 1.02 | 3.41 ± 1.06 | 3.05 ± 1.11 | 3.03 ± 0.90 | 3.61 ± 1.00 | 3.76 ± 0.77 | *** |
| The preventive use of veterinary drugs including antibiotics | 2.75 ± 1.18 | 2.22 ± 1.08 | 2.25 ± 1.12 | 2.59 ± 1.04 | 3.34 ± 1.13 | 3.32 ± 1.01 | *** |
| Use of feed supplements e.g. probiotics | 3.18 ± 1.10 | 3.02 ± 1.08 | 2.64 ± 1.19 | 3.26 ± 0.96 | 3.48 ± 1.02 | 3.44 ± 1.05 | *** |
| The use of vaccination | 3.52 ± 0.97 | 3.42 ± 1.02 | 3.36 ± 1.06 | 3.40 ± 0.95 | 3.66 ± 0.97 | 3.76 ± 0.81 | *** |
| Efficient monitoring of pigs and pig housing conditions | 4.17 ± 0.87 | 4.36 ± 0.73 | 4.25 ± 0.93 | 3.97 ± 0.88 | 4.11 ± 0.95 | 4.19 ± 0.80 | *** |
| Enhanced control of air movement in pig houses | 4.16 ± 0.87 | 4.35 ± 0.73 | 4.20 ± 0.88 | 4.06 ± 0.92 | 4.16 ± 0.91 | 4.05 ± 0.89 | * |
| Improvements in pigs' diet composition | 4.18 ± 0.88 | 4.33 ± 0.73 | 4.21 ± 0.95 | 4.05 ± 0.92 | 4.15 ± 0.93 | 4.20 ± 0.85 |  |
| Adjustments in the quantity of pig feed available | 3.85 ± 0.91 | 3.66 ± 0.85 | 3.87 ± 0.94 | 3.70 ± 0.94 | 4.13 ± 0.84 | 3.96 ± 0.89 | *** |
| Breeding for genetically tougher or more resilient pigs | 3.26 ± 1.14 | 3.39 ± 1.11 | 2.61 ± 1.20 | 3.31 ± 1.10 | 3.59 ± 1.04 | 3.40 ± 1.06 | *** |
| Improvements in housing design | 4.13 ± 0.91 | 4.29 ± 0.80 | 4.09 ± 0.99 | 4.01 ± 0.89 | 4.16 ± 0.90 | 4.09 ± 0.87 |  |
| Housing that protects pigs from adverse natural conditions | 3.96 ± 0.94 | 4.01 ± 0.93 | 3.65 ± 1.05 | 3.94 ± 0.88 | 4.16 ± 0.86 | 4.03 ± 0.89 | *** |
| Reducing the number of pigs in a given area | 4.13 ± 0.91 | 4.22 ± 0.90 | 4.30 ± 0.97 | 3.95 ± 0.89 | 4.04 ± 0.95 | 4.18 ± 0.80 | *** |
| Providing enrichment materials so pigs can perform natural behaviours | 4.19 ± 0.88 | 4.35 ± 0.79 | 4.42 ± 0.88 | 3.97 ± 0.88 | 4.14 ± 0.92 | 4.08 ± 0.85 | *** |
| Providing farmers with a price premium that encourages enhanced animal health | 4.09 ± 0.92 | 3.97 ± 1.00 | 4.27 ± 0.93 | 4.03 ± 0.84 | 4.23 ± 0.86 | 3.99 ± 0.92 | ** |
| Doing nothing | 2.36 ± 1.30 | 1.97 ± 1.12 | 1.59 ± 0.95 | 2.25 ± 1.11 | 3.67 ± 0.96 | 2.26 ± 1.27 | *** |

***Table N Intervention preferences for pigs in intensive production systems among respondents in five countries (mean response on a linear scale 1 (Strongly disagree) to 5 (Strongly agree) ± SD.***

Significance values for Chi-square test to indicate between country differences: **p< 0.05, ** p< 0.01 and *** p<0.001*)

## D: Faceted bar charts of the rationale behind acceptance of interventions

All responses are reported as count of responses for each scale response. Responses are based on a 1 (strongly disagree) to 5(strongly agree) Likert scale. Categories (x axis) are based on Q14, reason for the acceptability rating given.

## Layers (n=790)

| 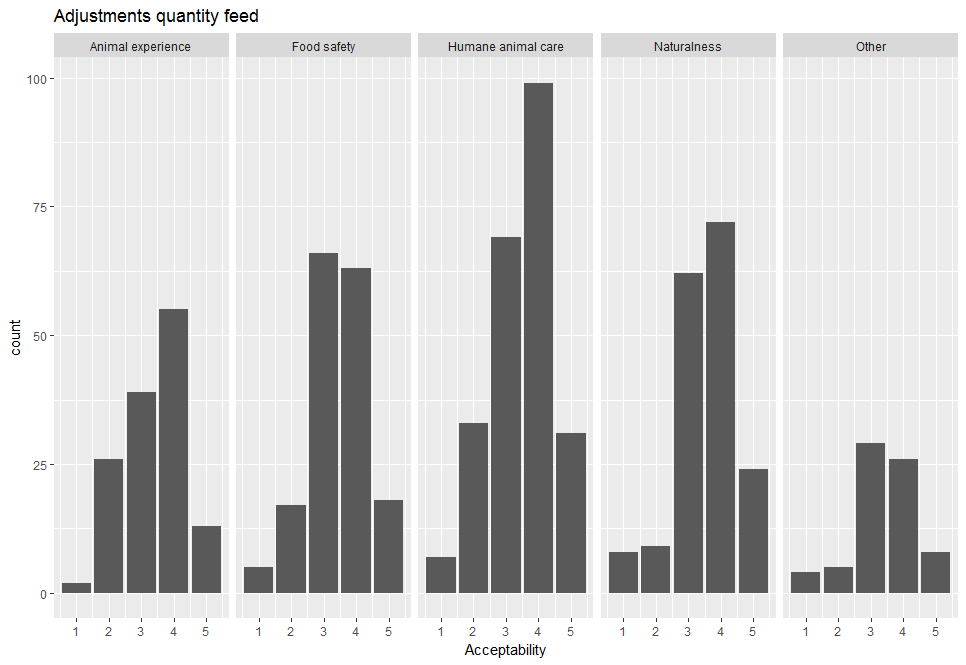 | 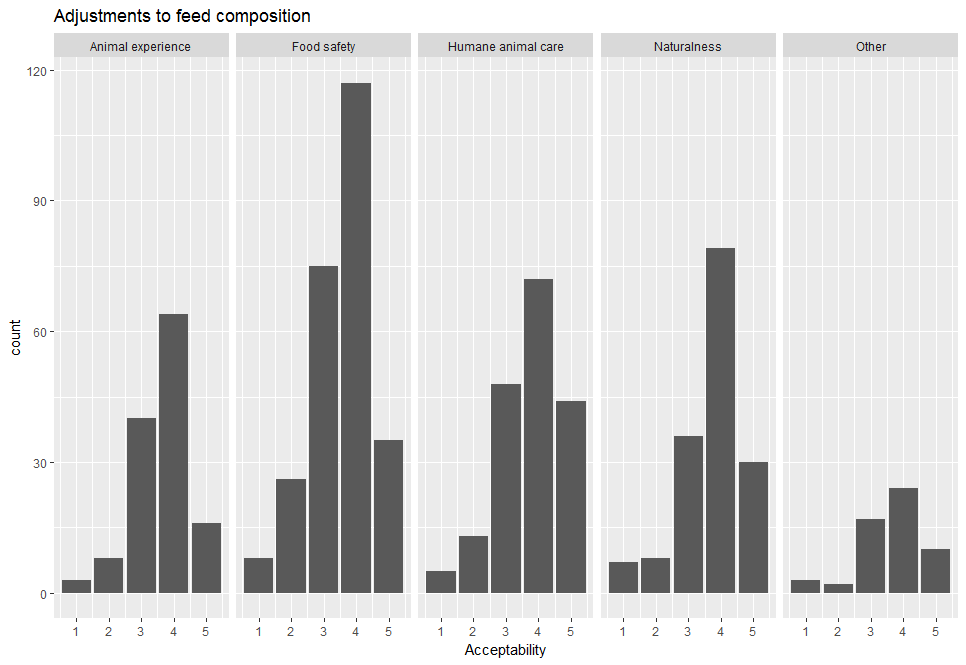 |
| --- | --- |
| 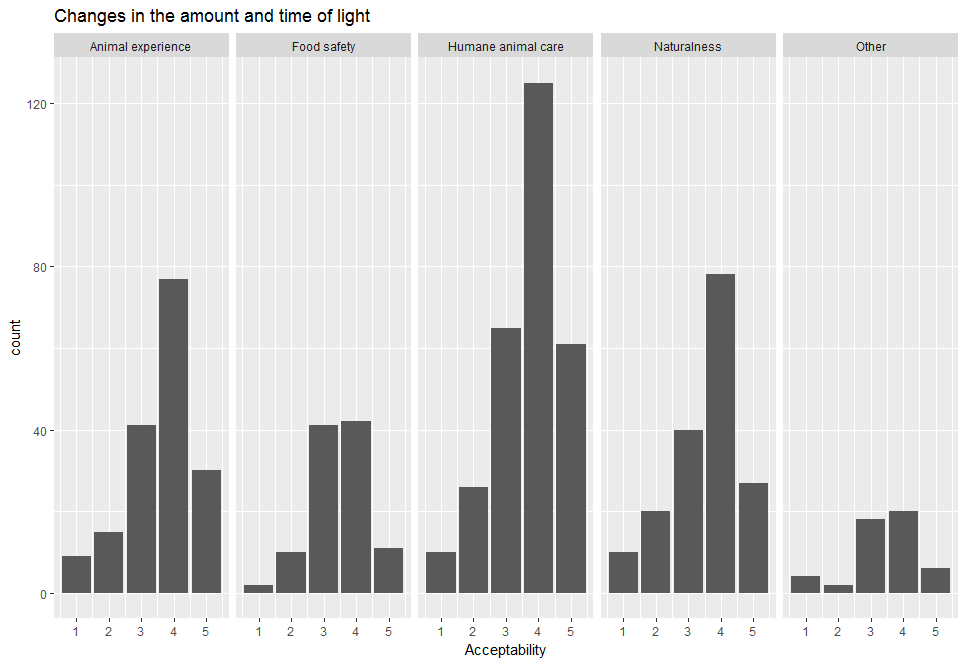 | 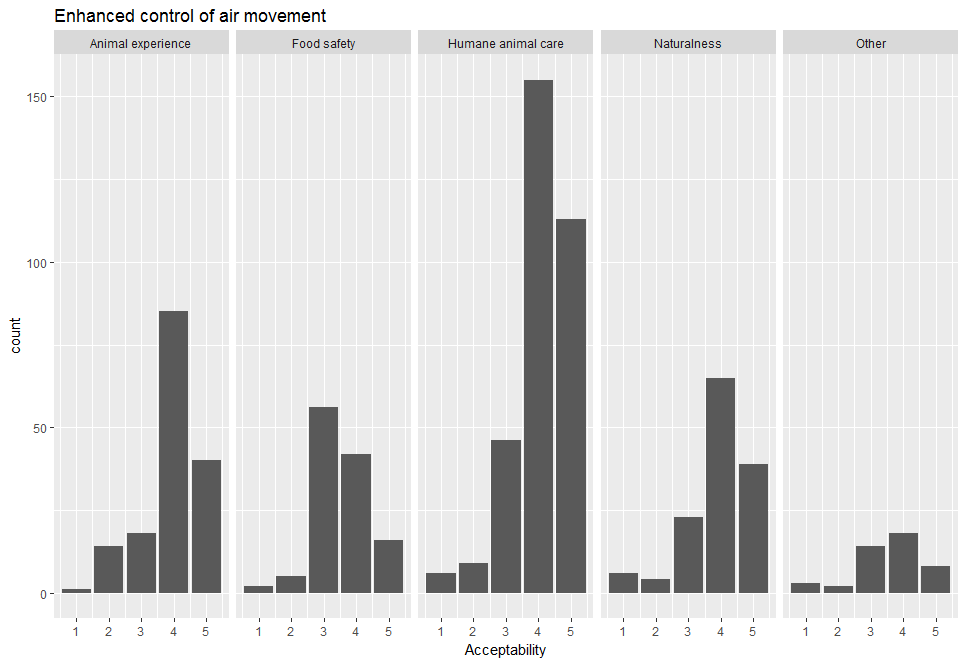 |
| 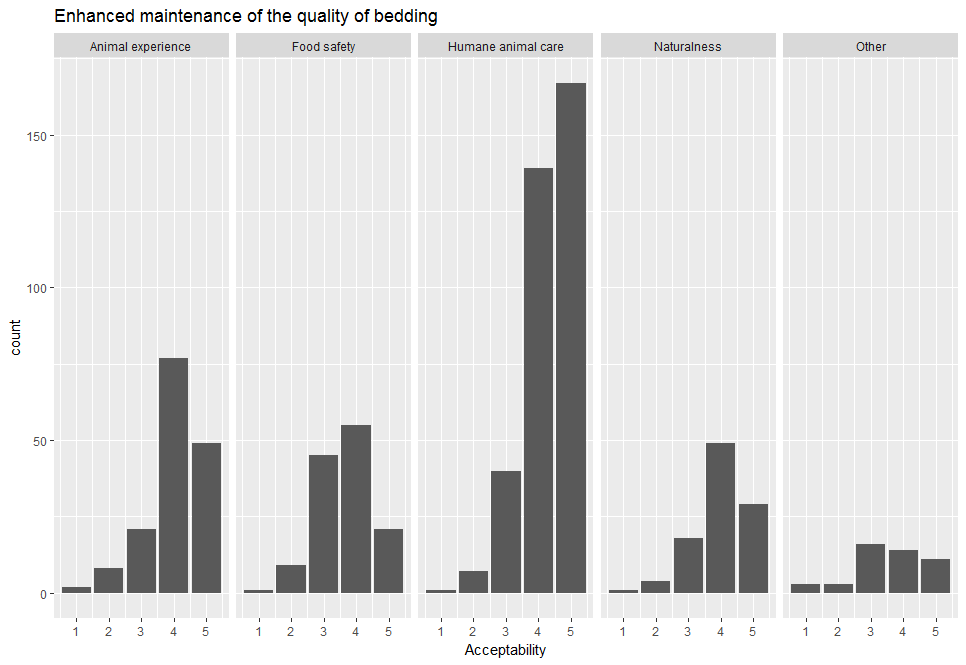 | 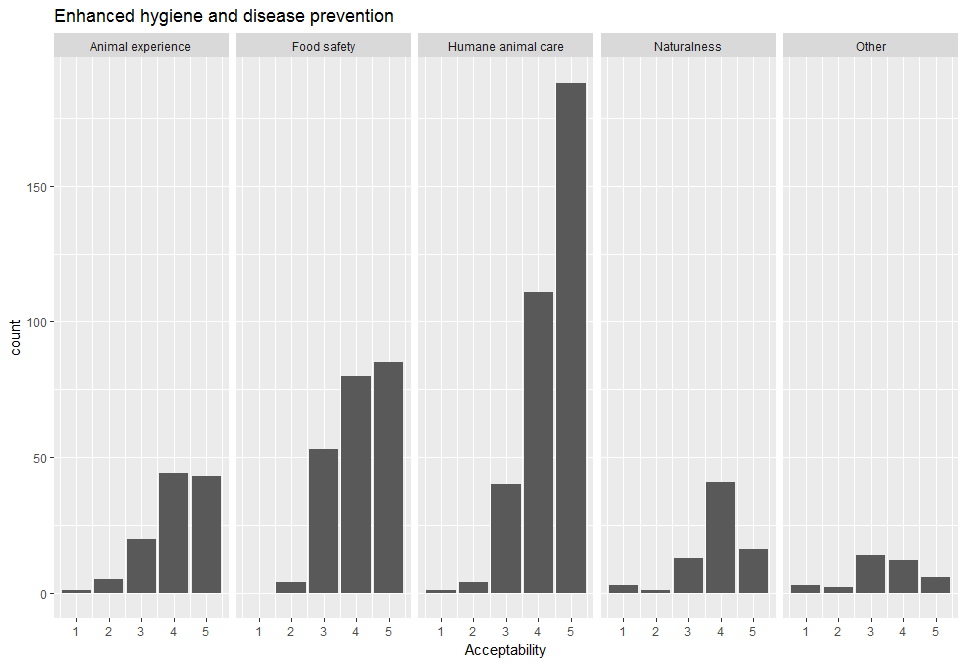 |
| 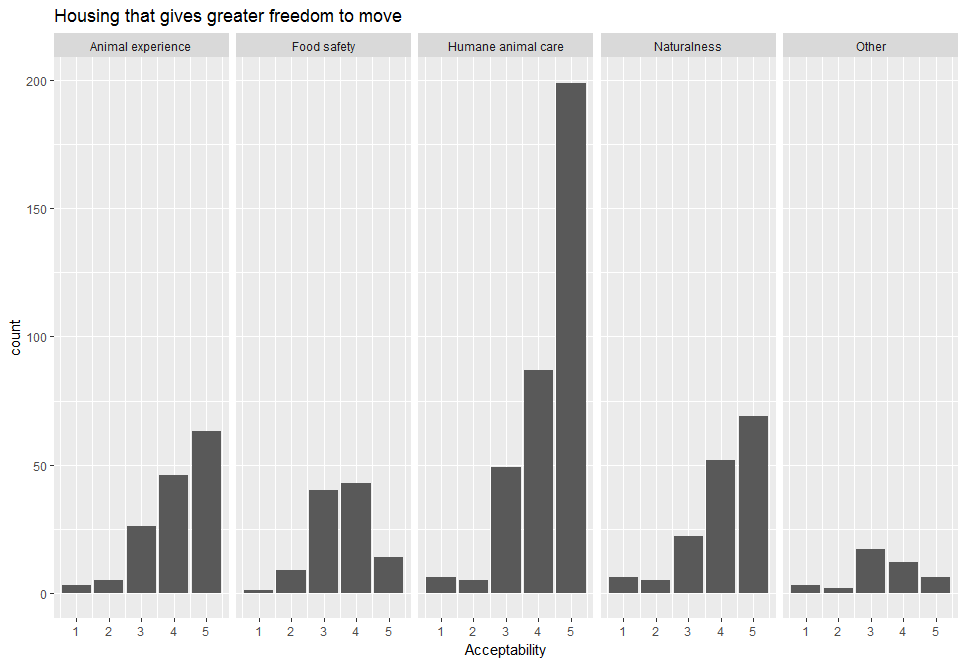 | 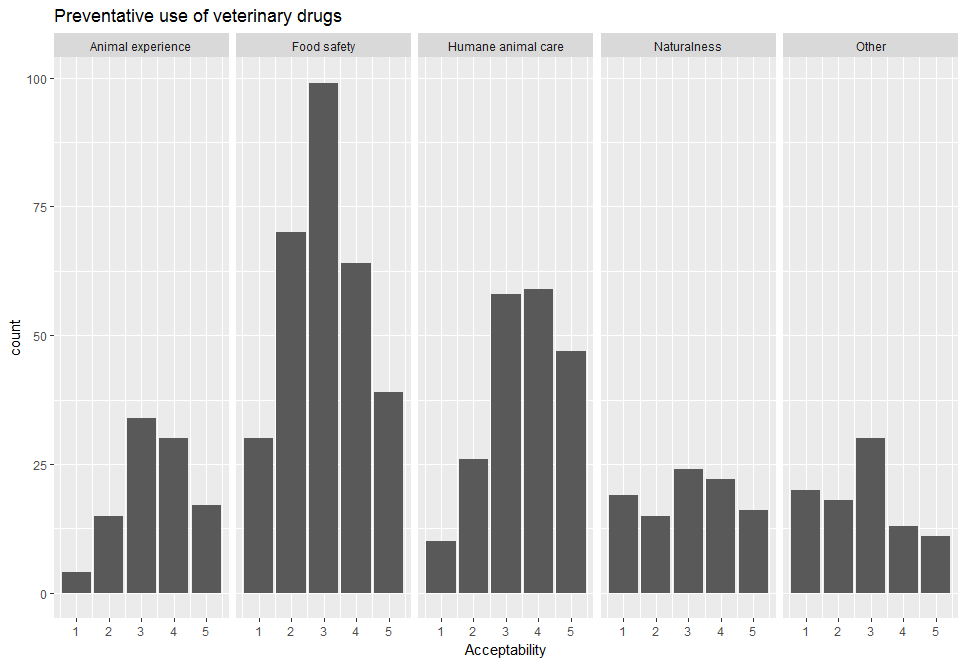 |
| 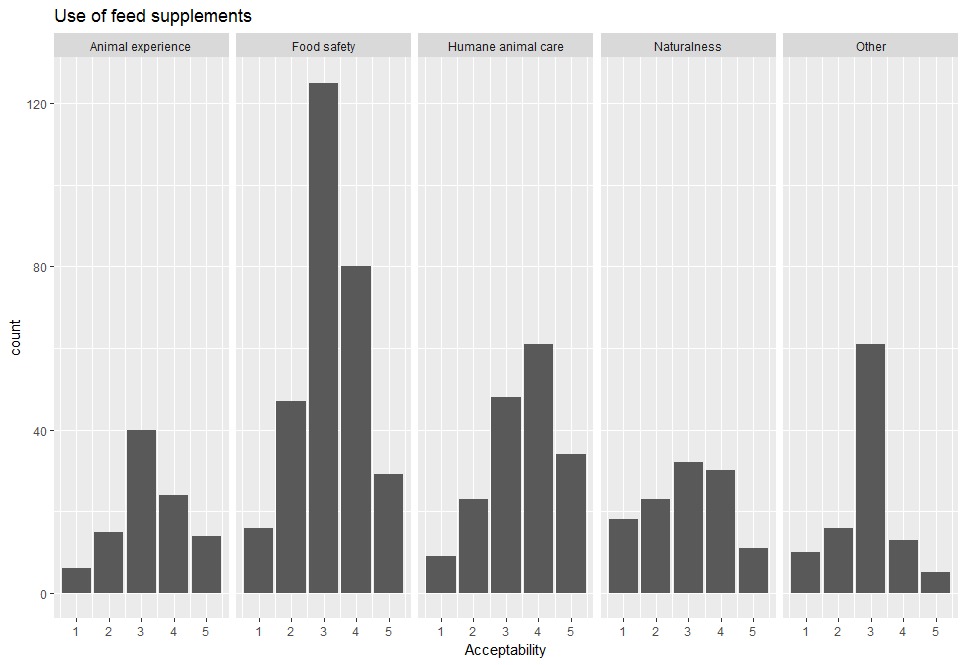 | 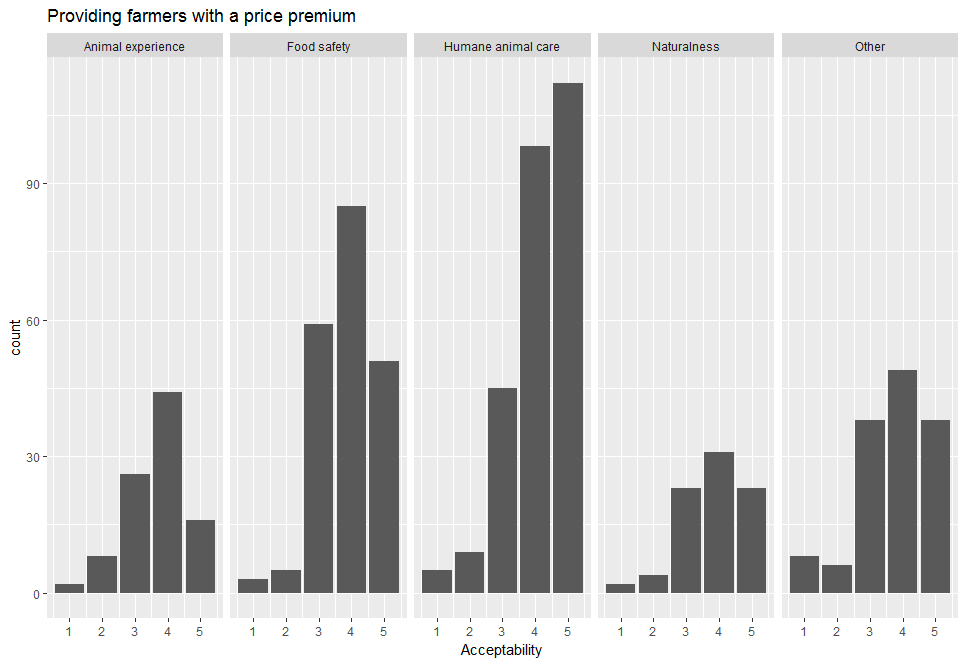 |
| 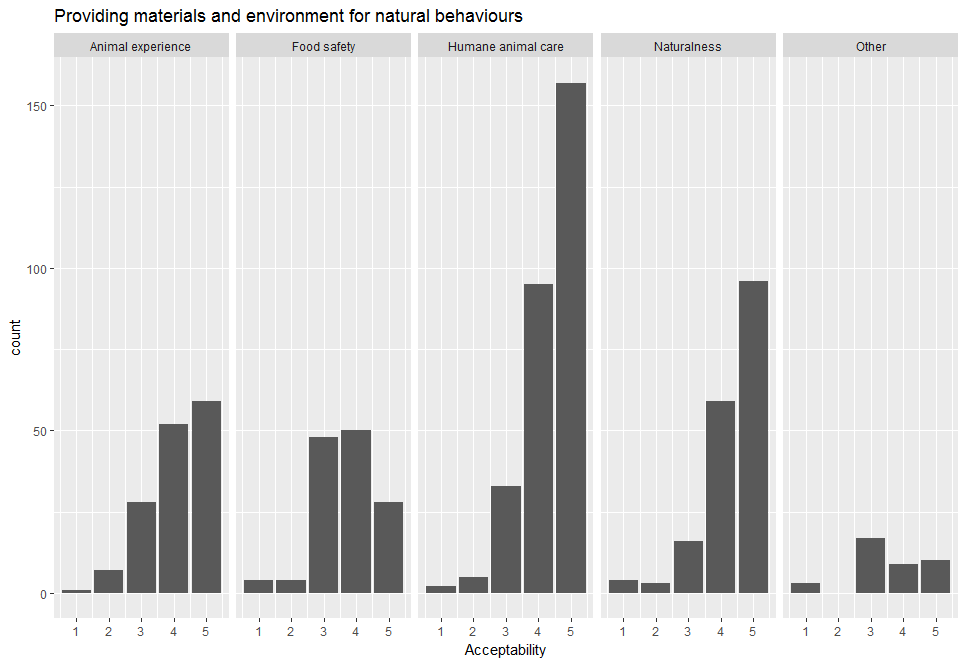 | 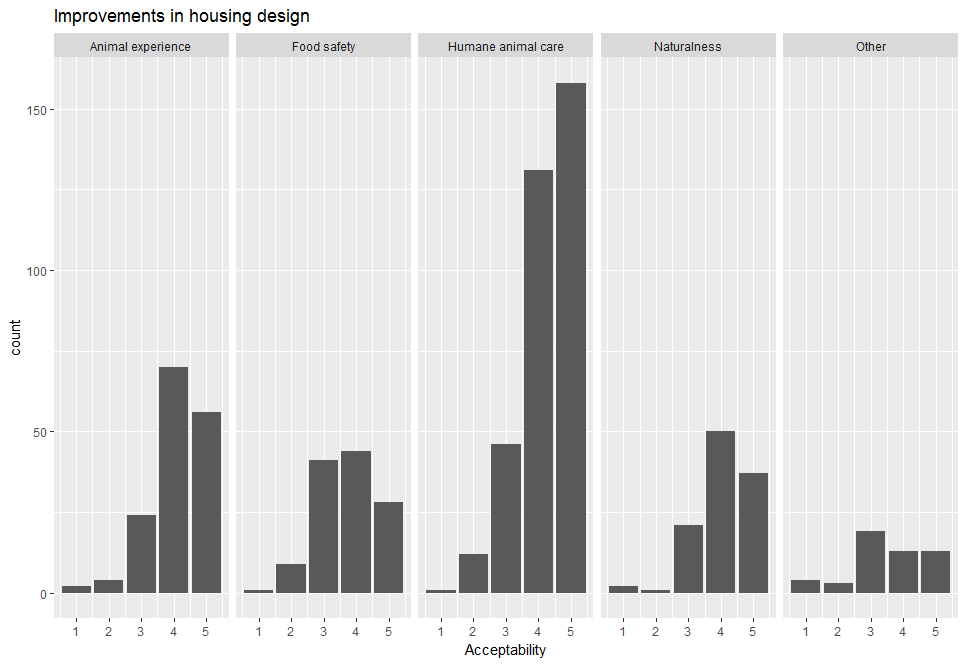 |
| 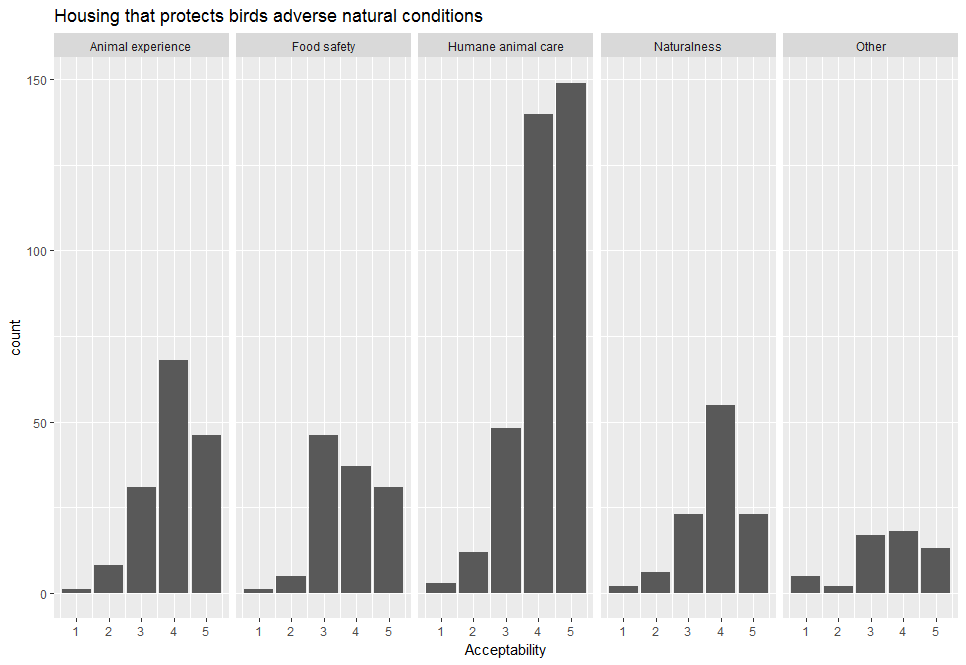 | 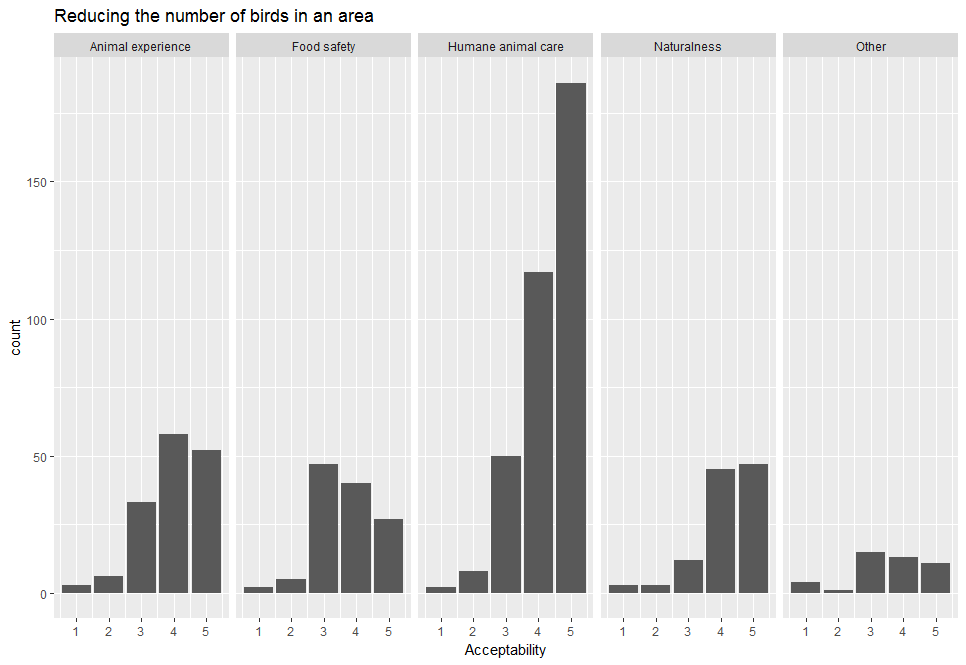 |
| 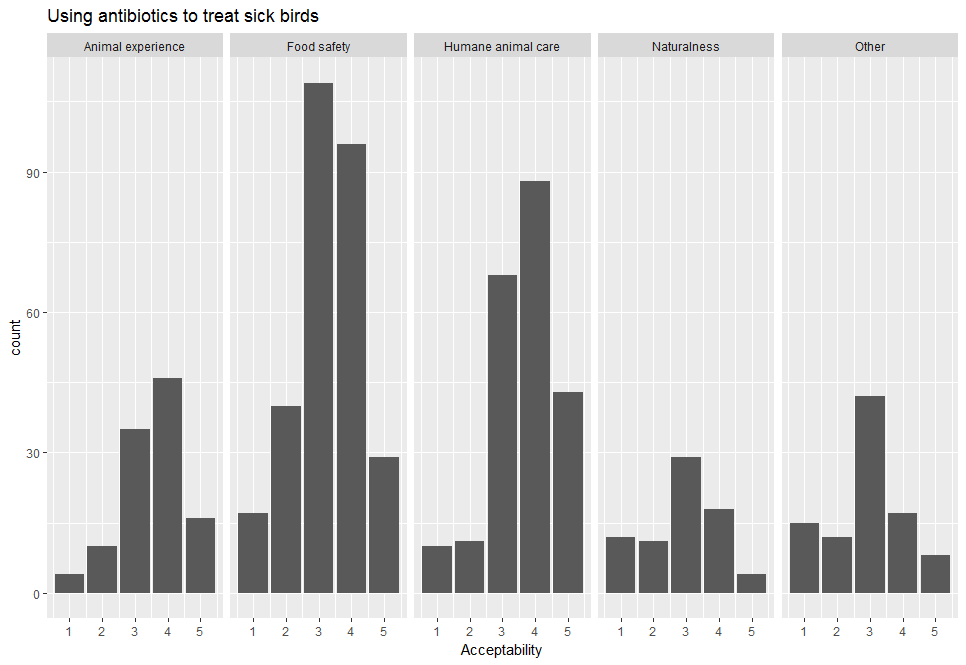 | 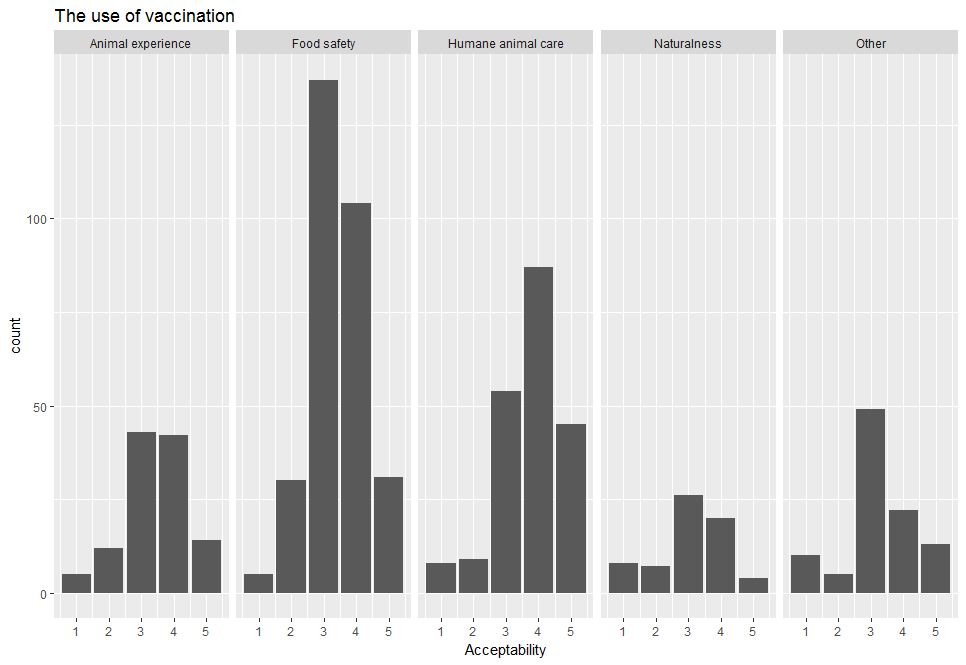 |
| 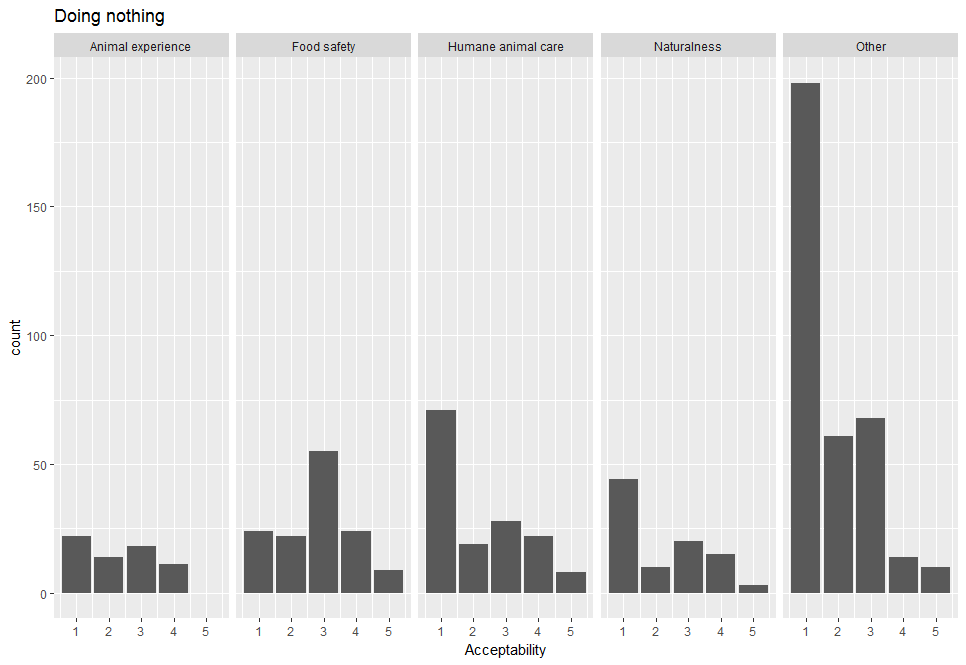 |  |

## Broilers (n=789)

| 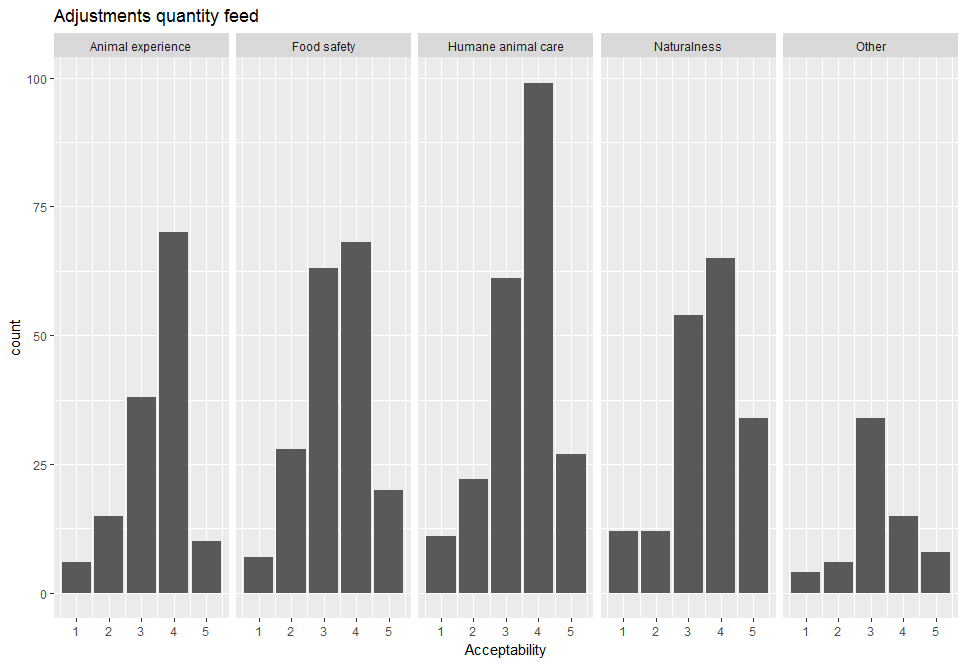 | 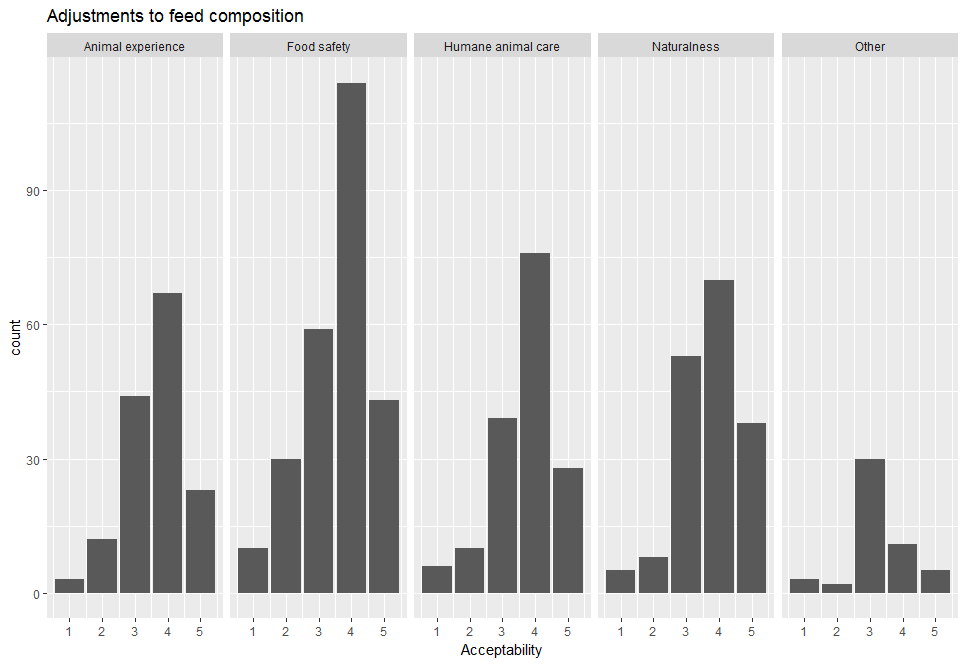 |
| --- | --- |
| 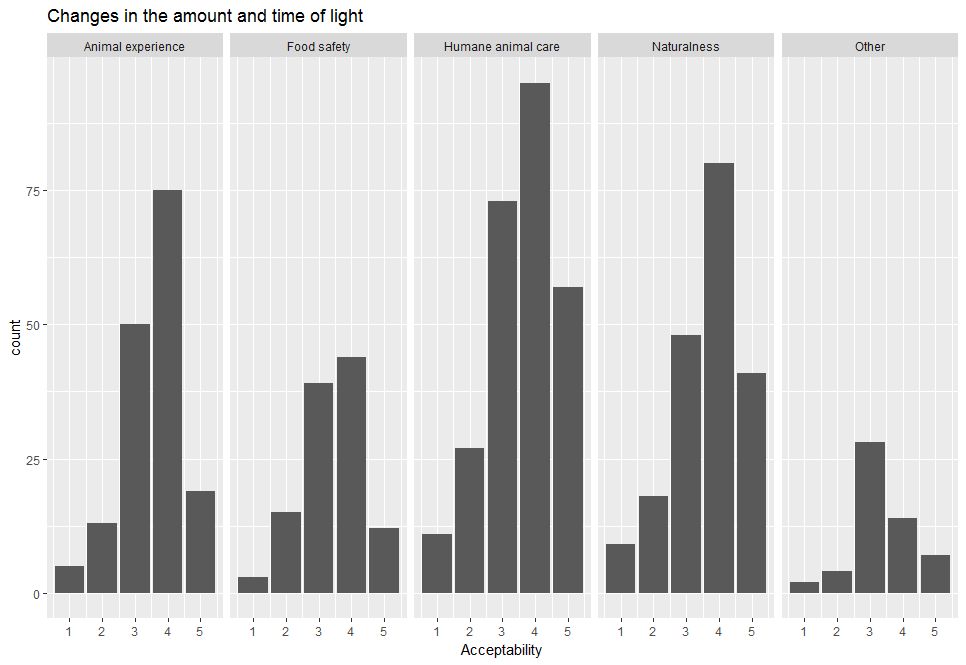 | 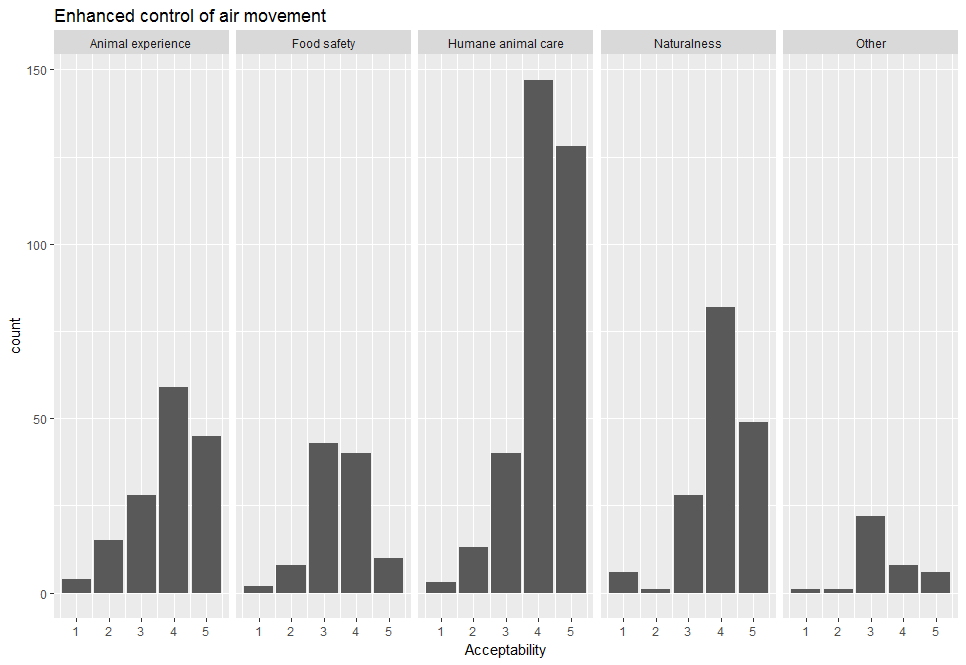 |
| 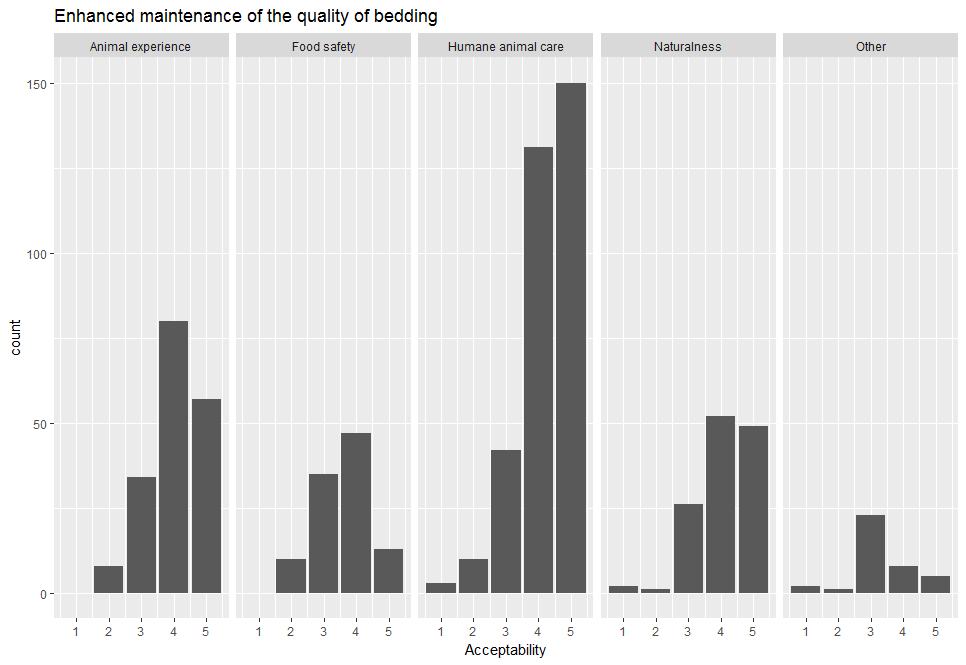 | 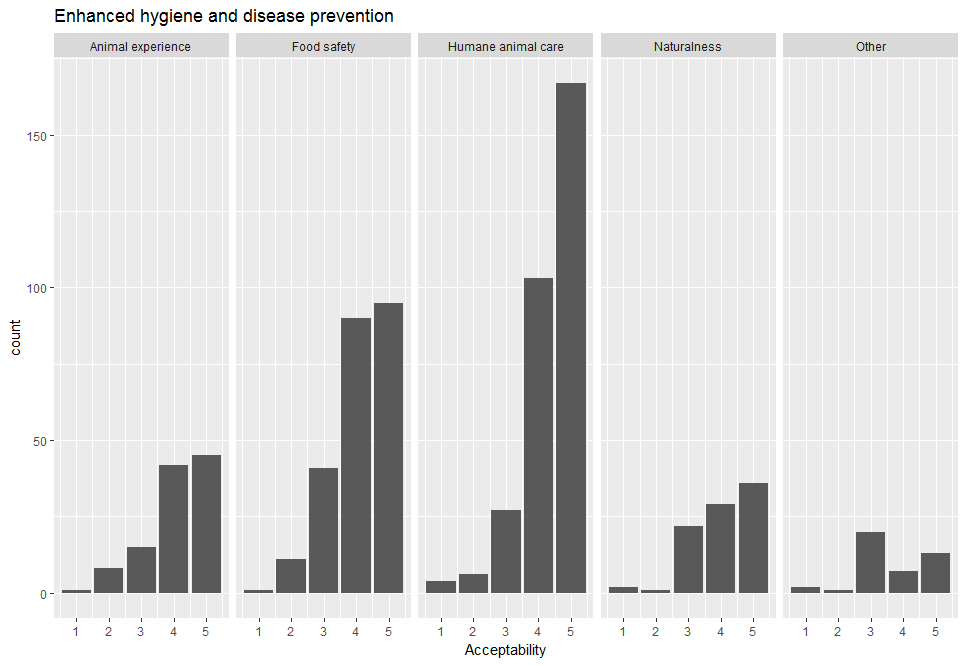 |
| 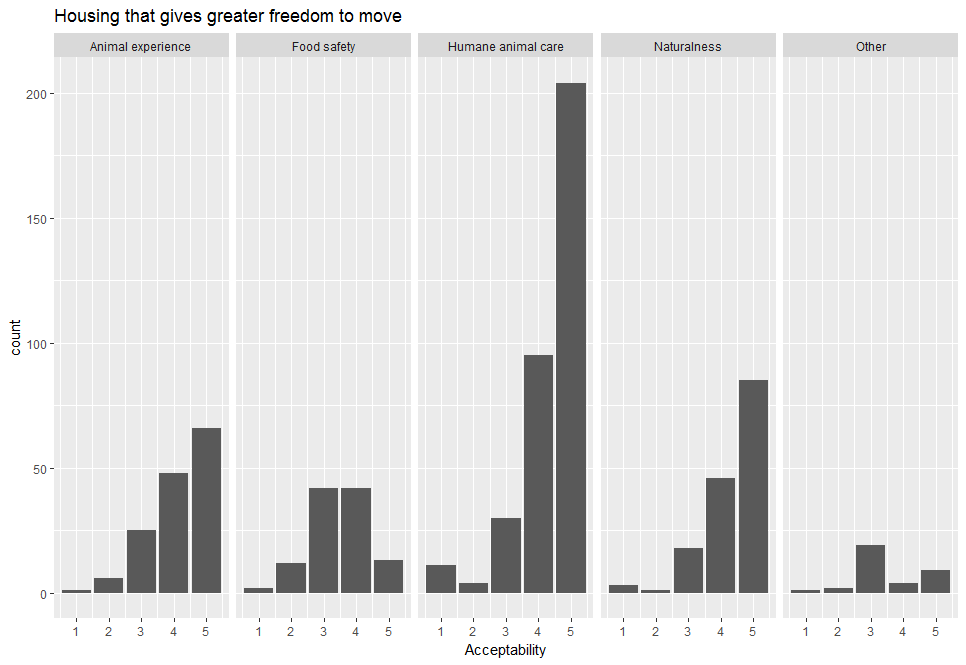 | 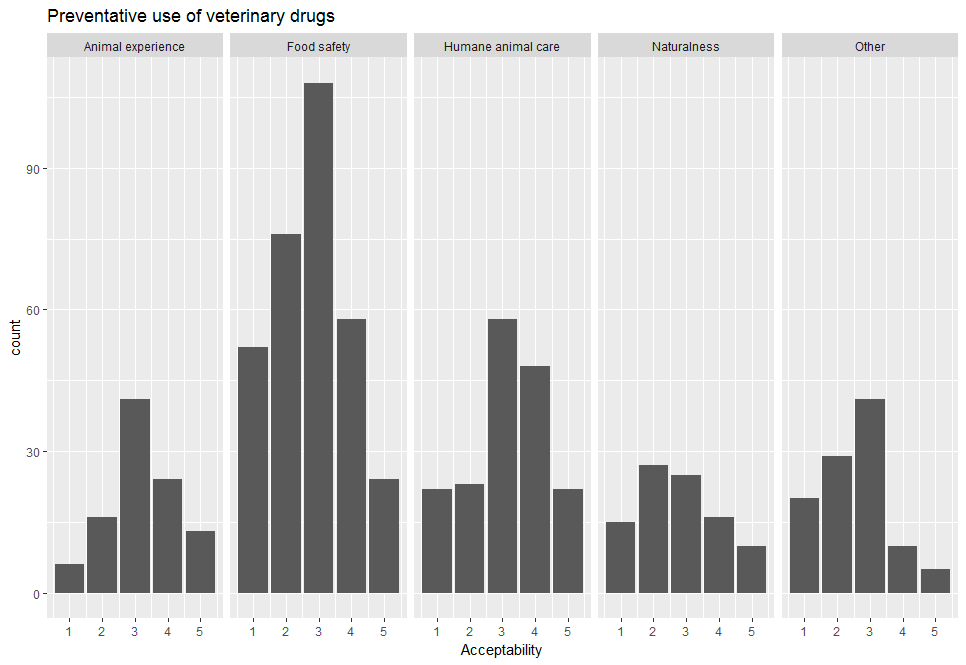 |
| 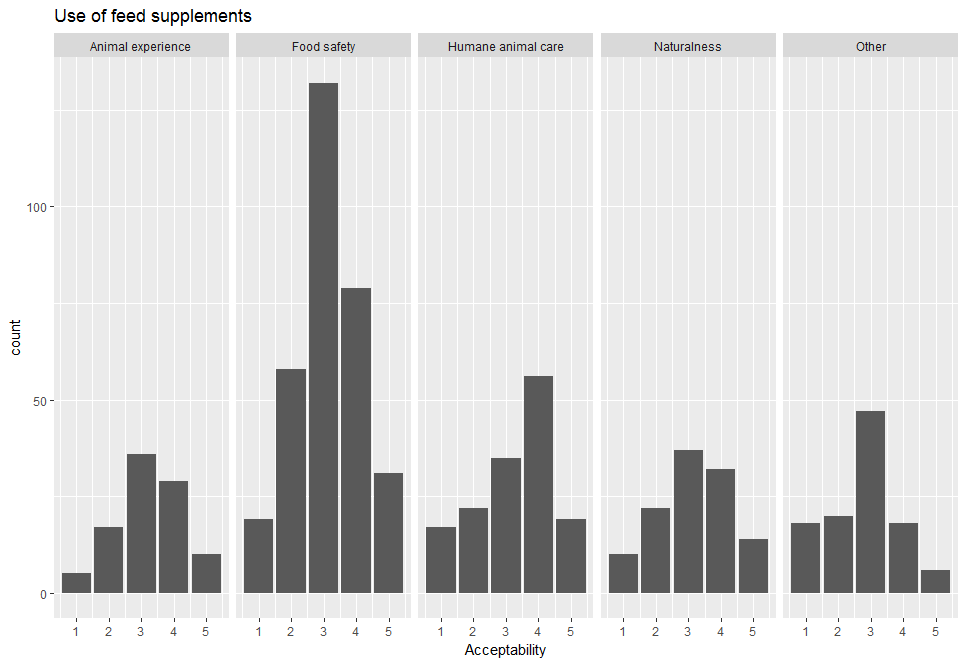 | 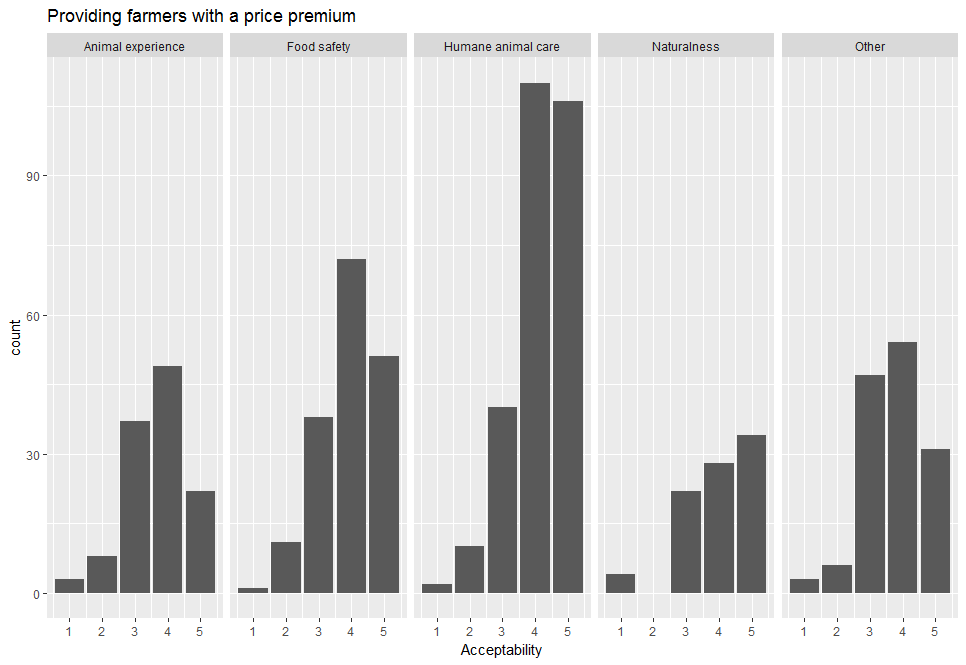 |
| 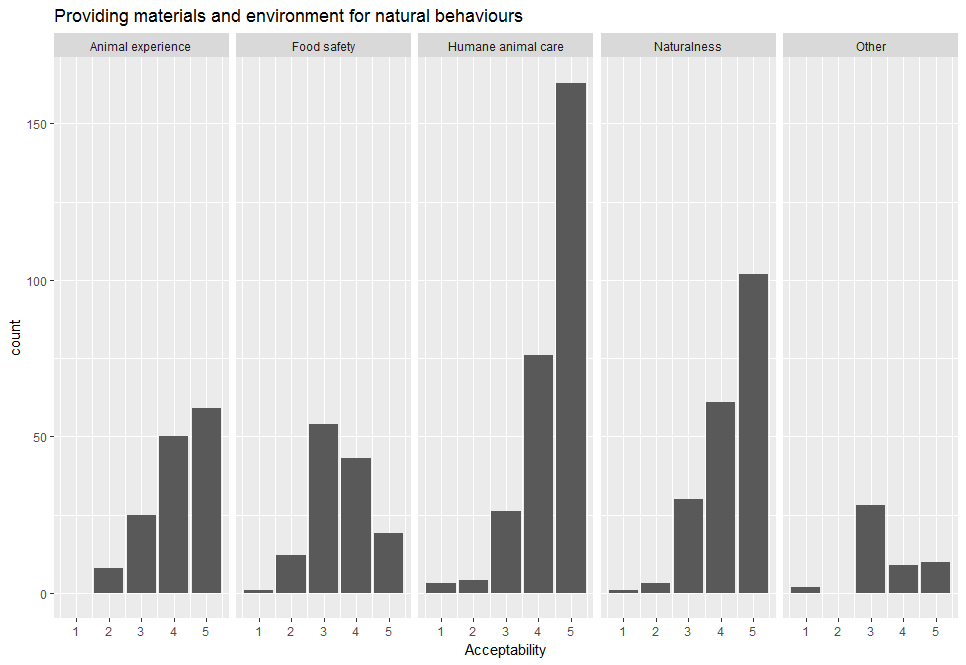 | 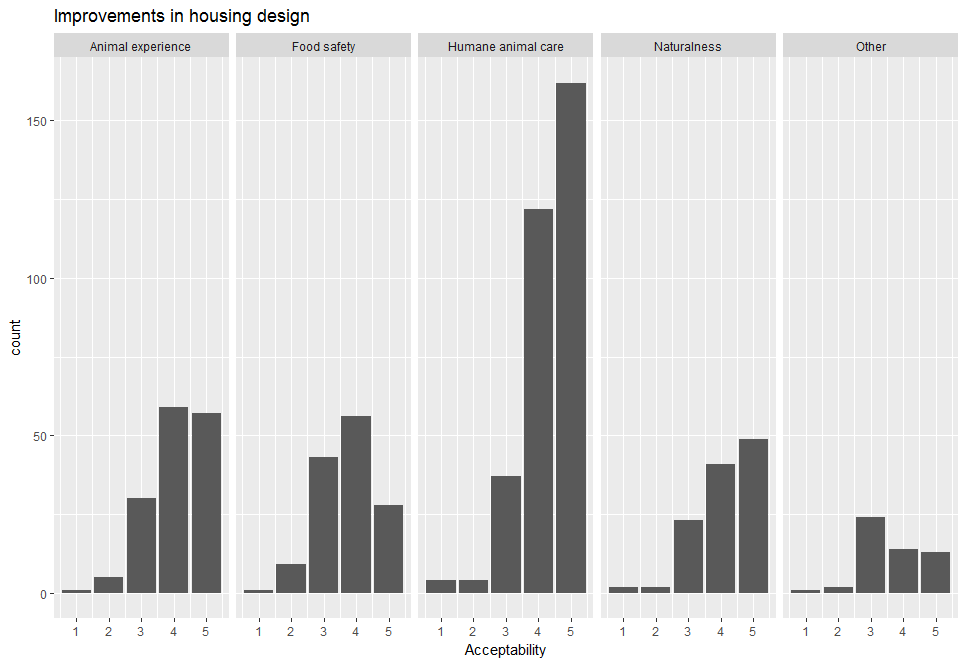 |
| 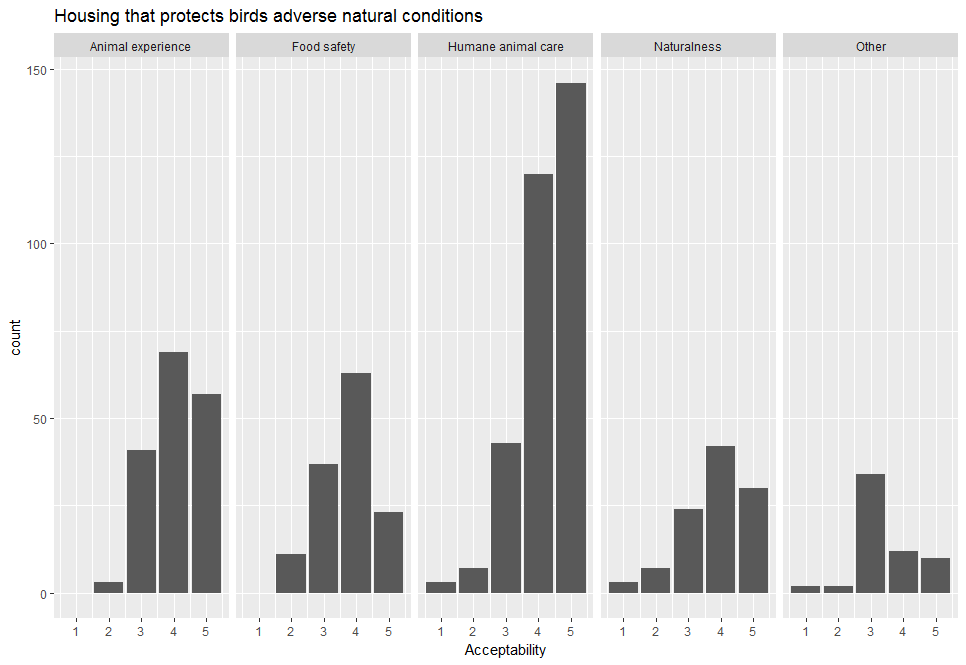 | 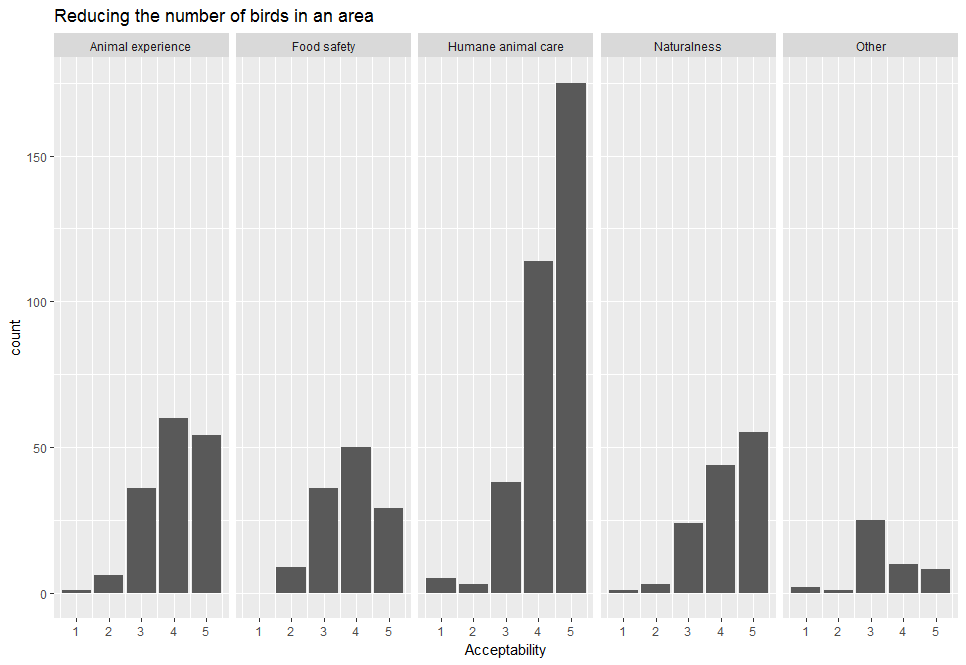 |
| 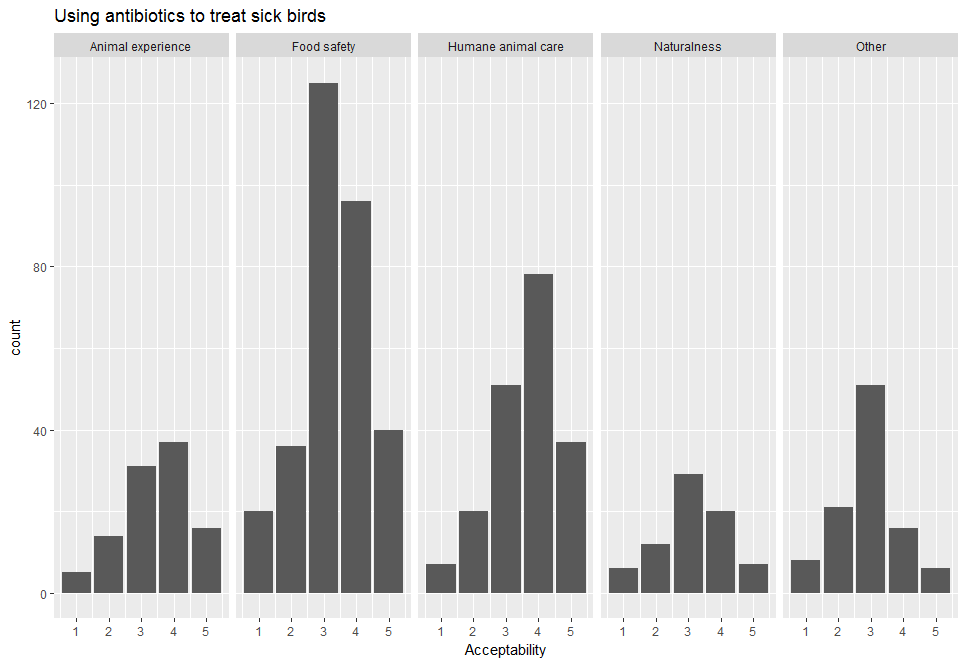 | 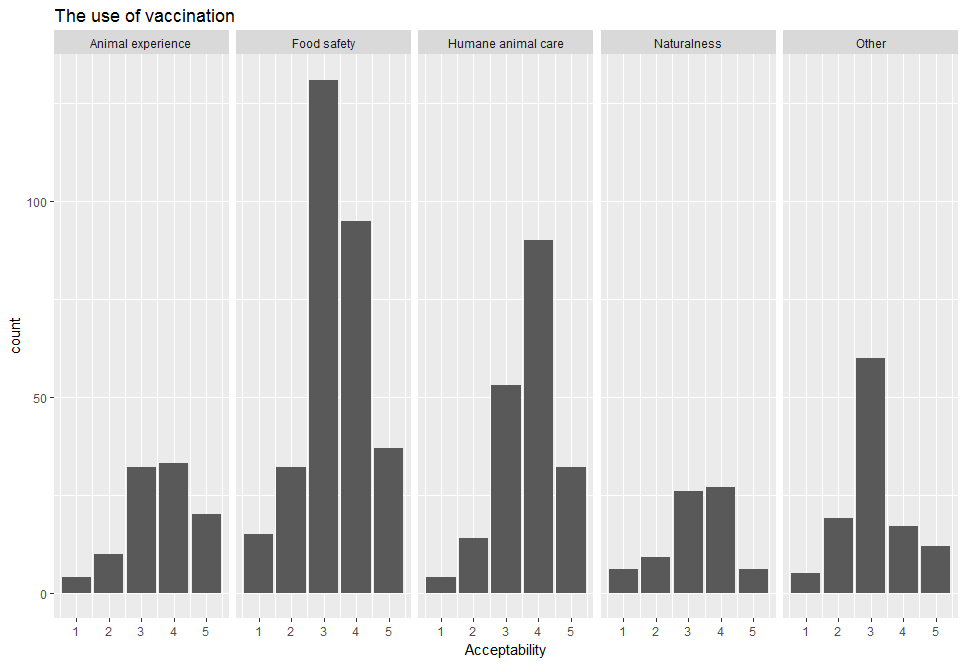 |
| 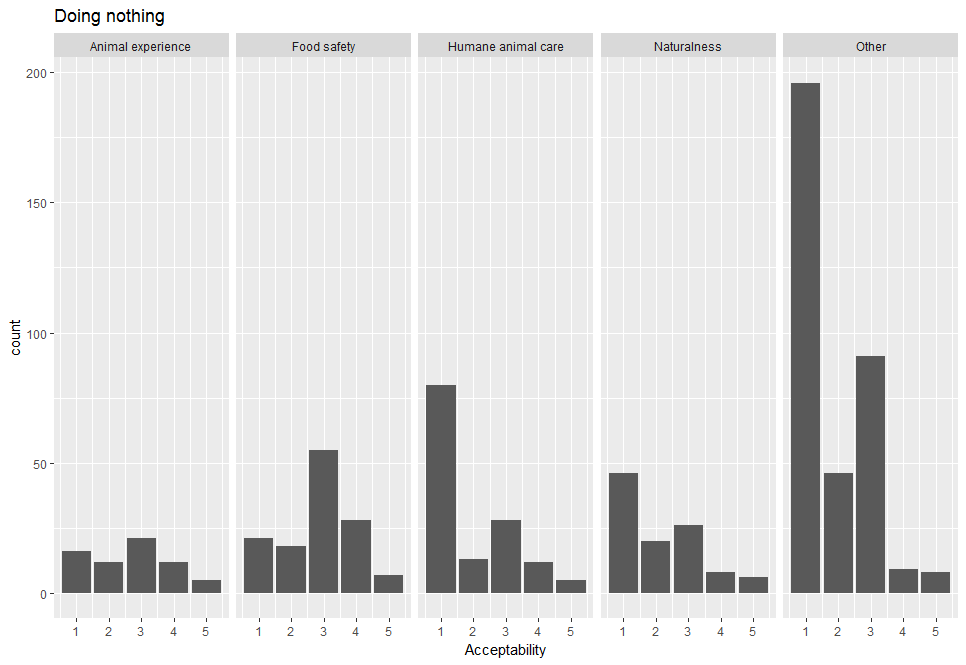 |  |

## Pigs (n=751)

| 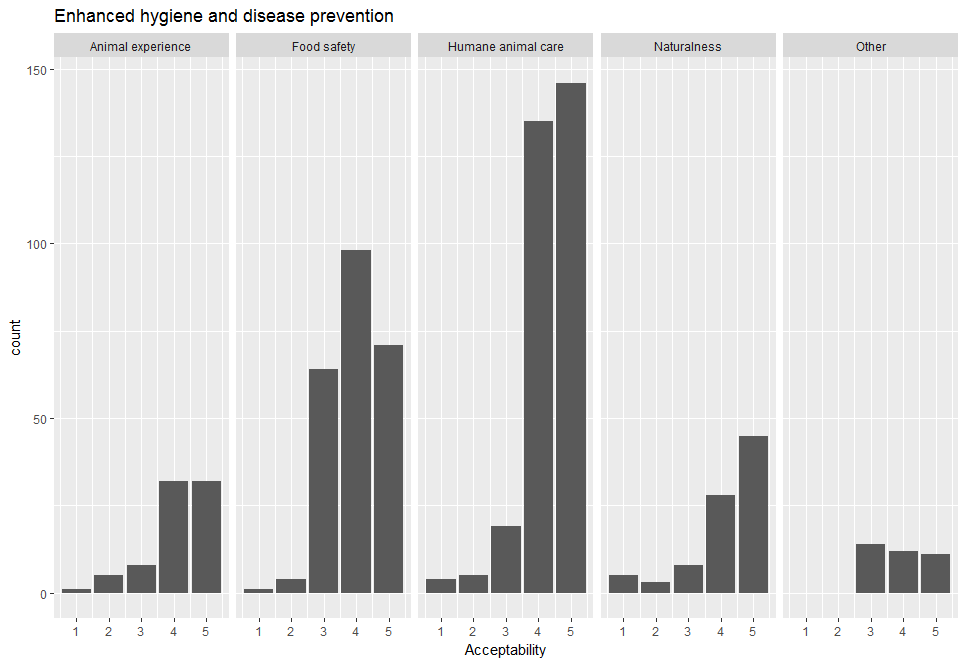 | 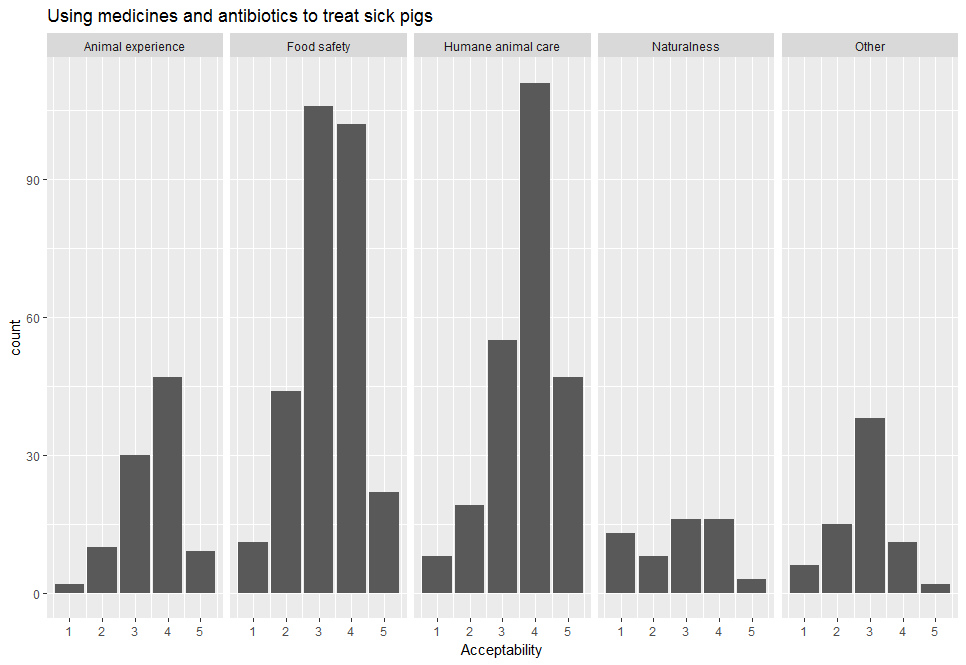 |
| --- | --- |
| 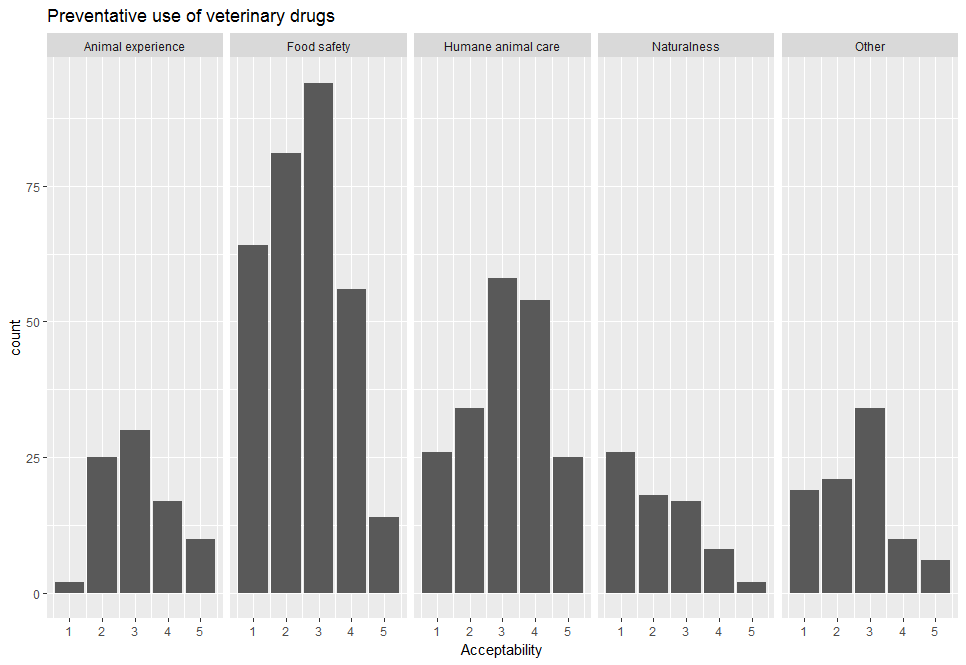 | 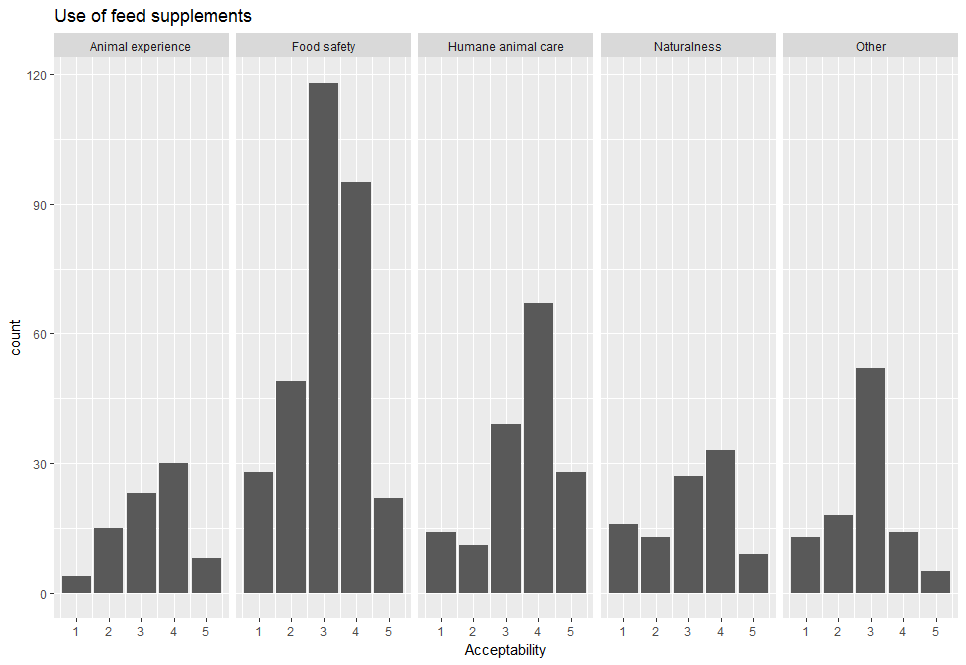 |
| 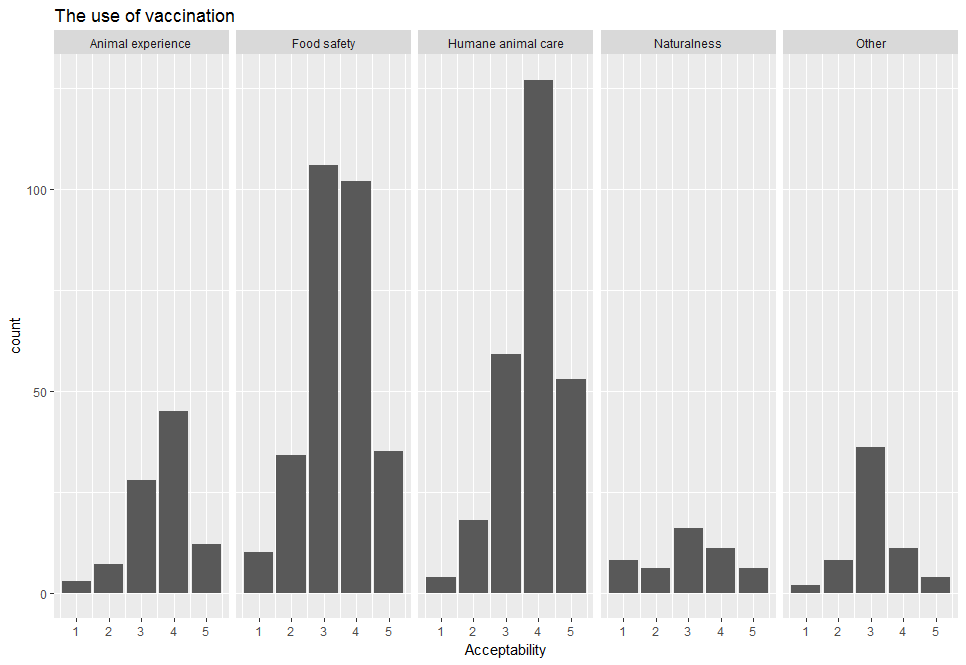 | 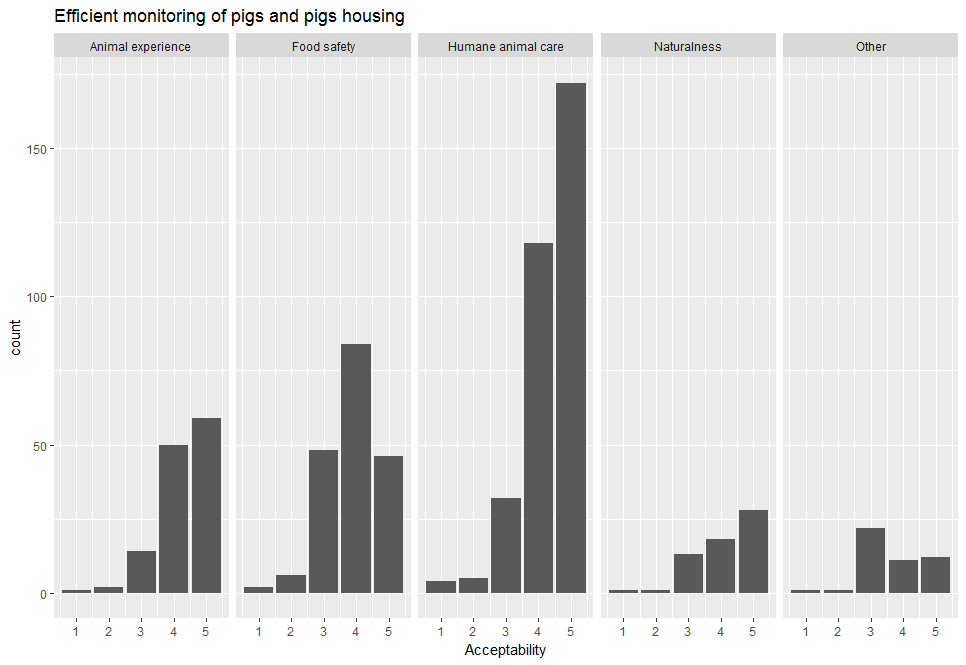 |
| 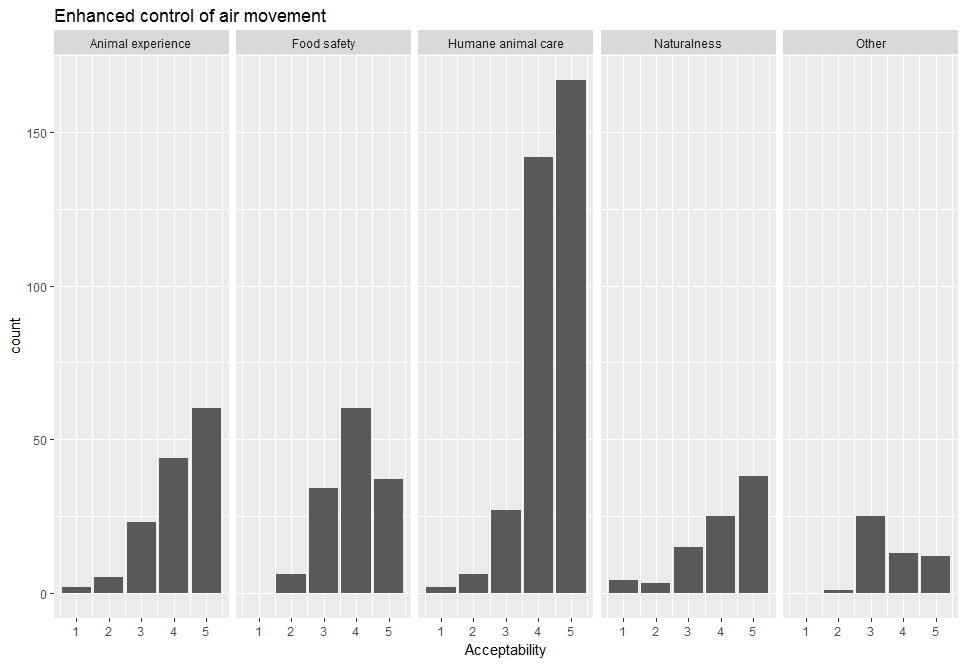 | 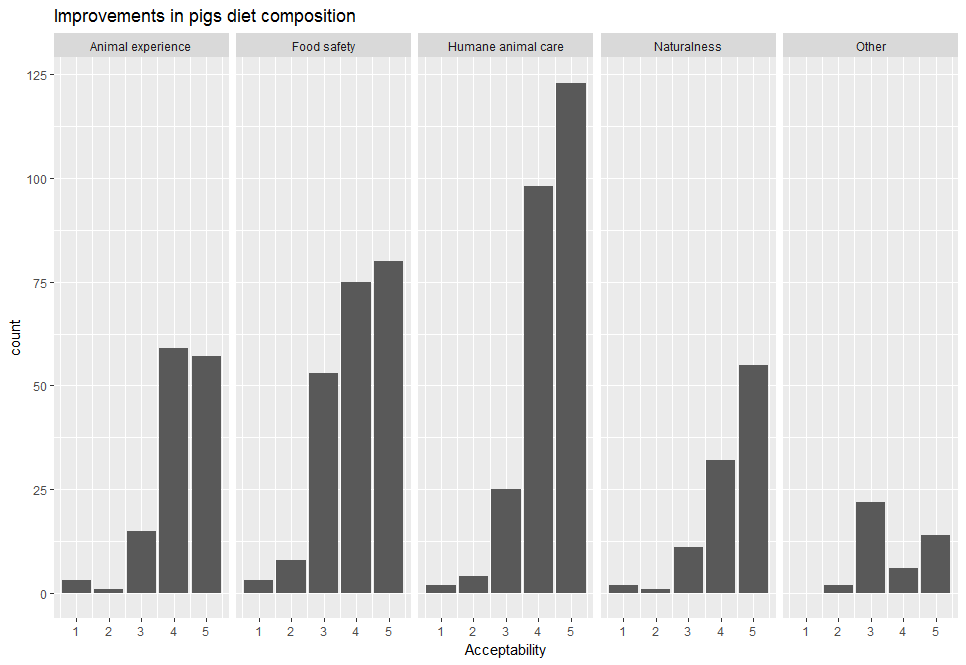 |
| 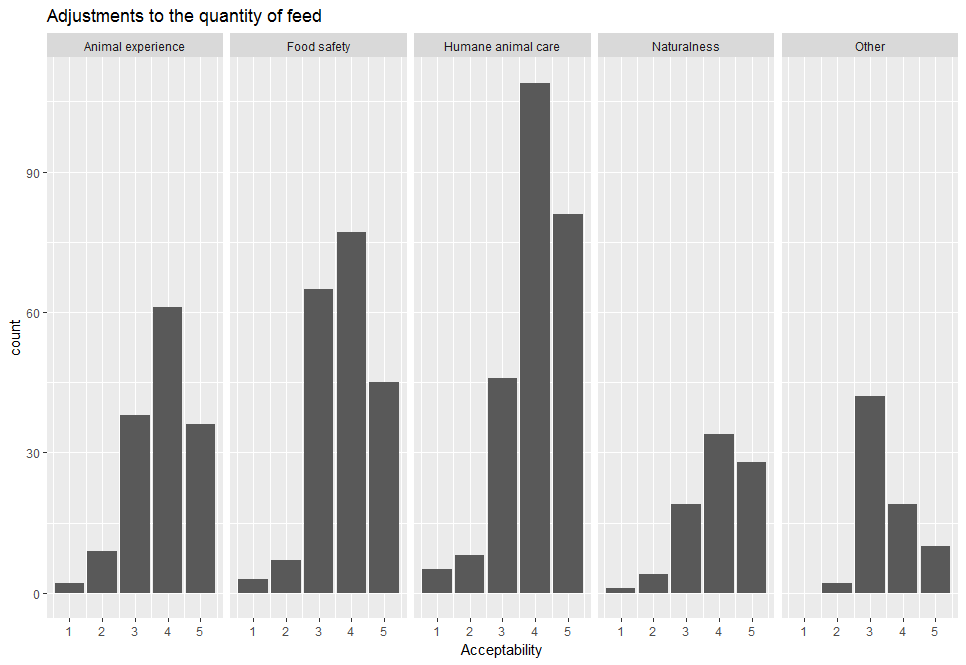 | 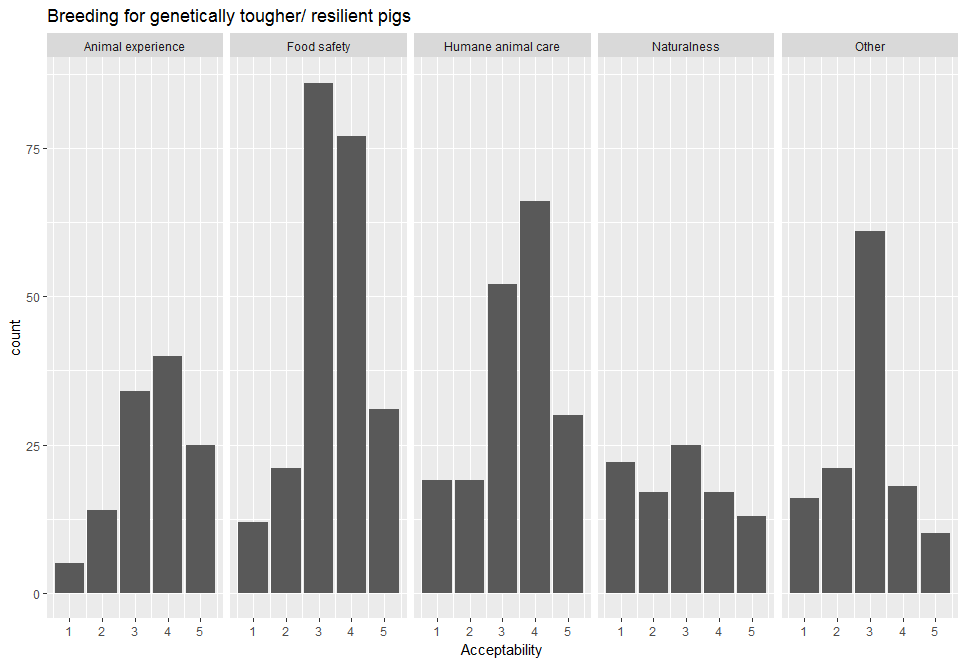 |
| 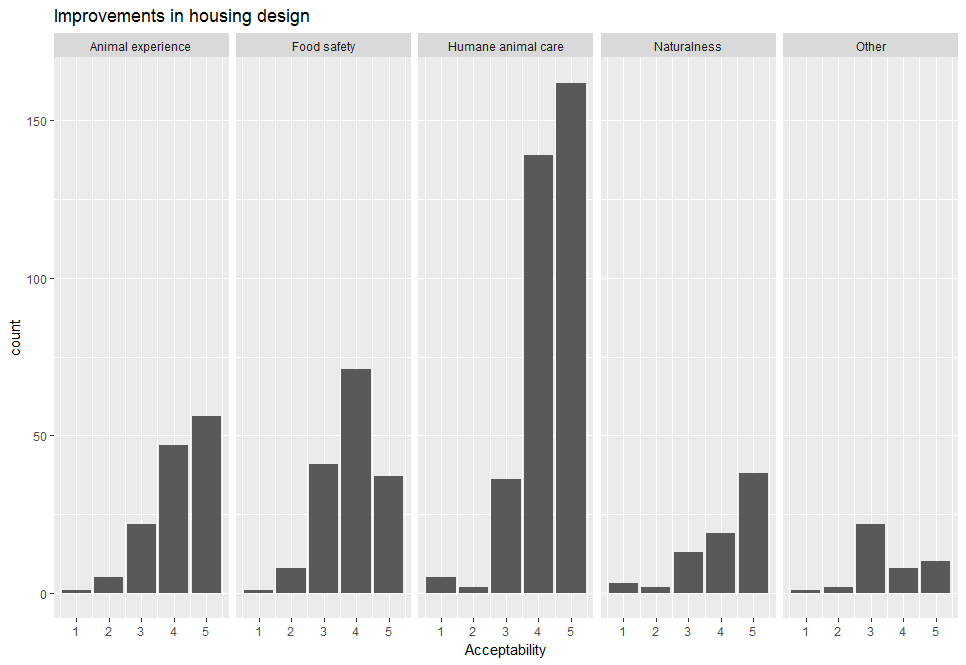 | 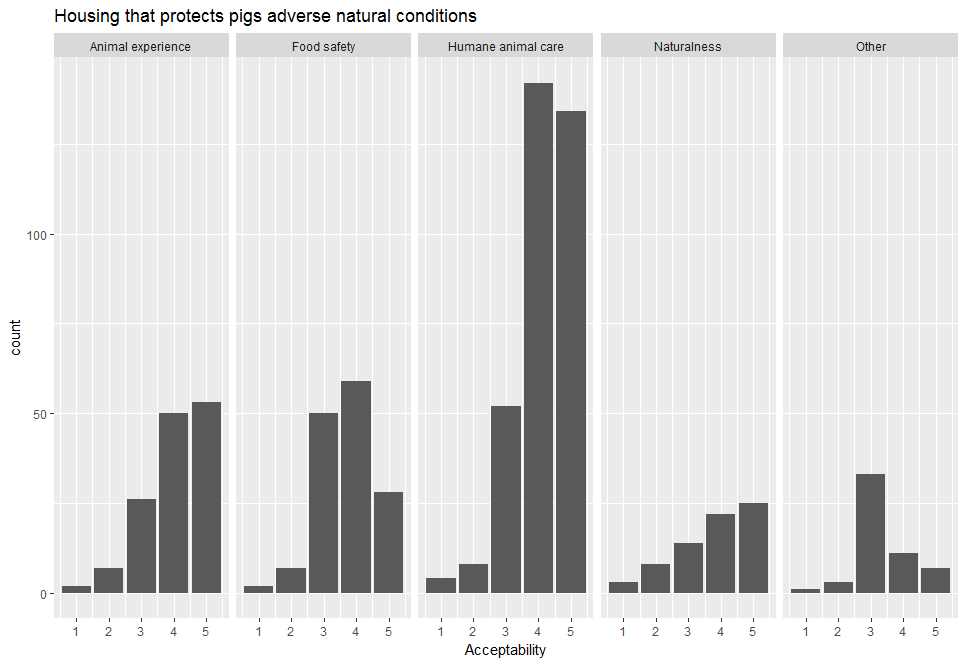 |
| 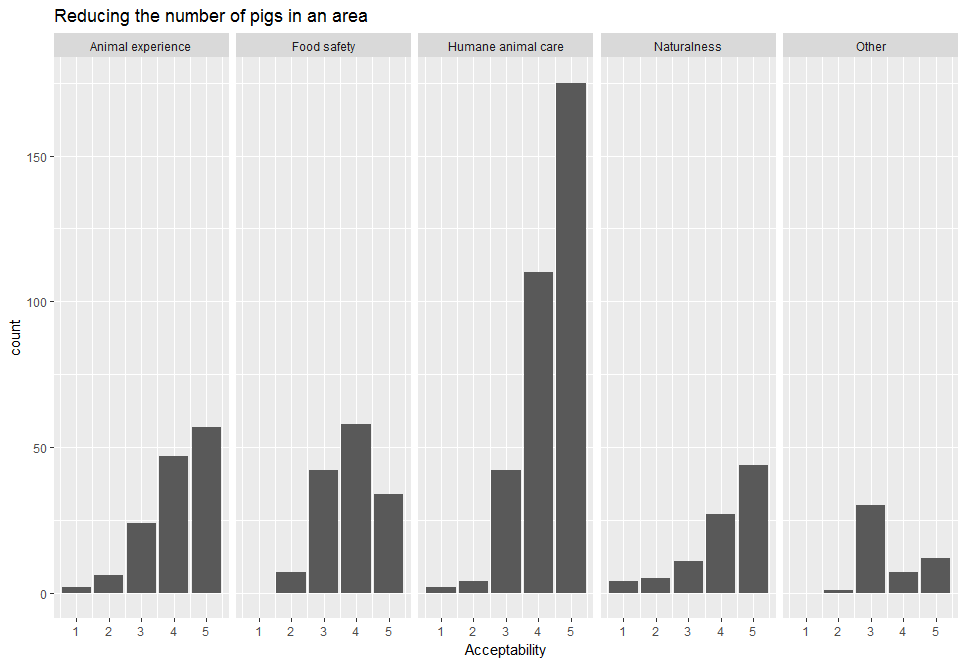 | 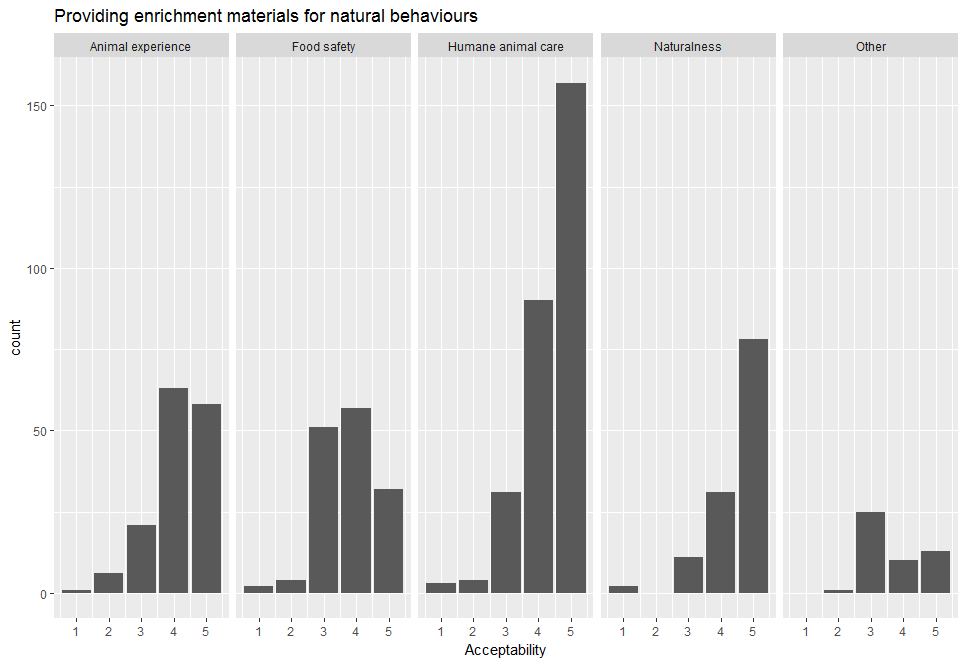 |
| 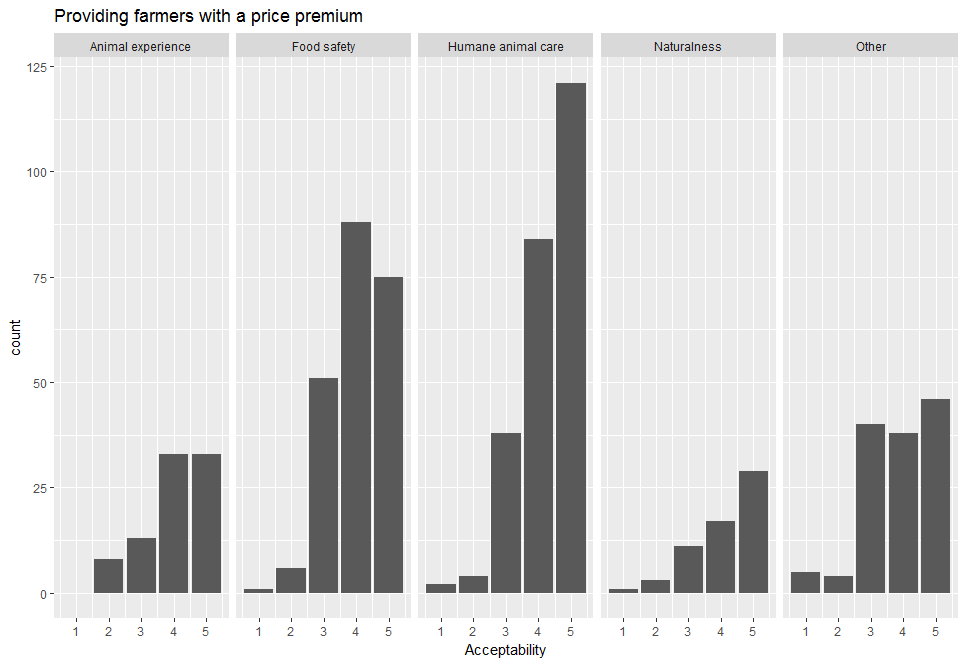 | 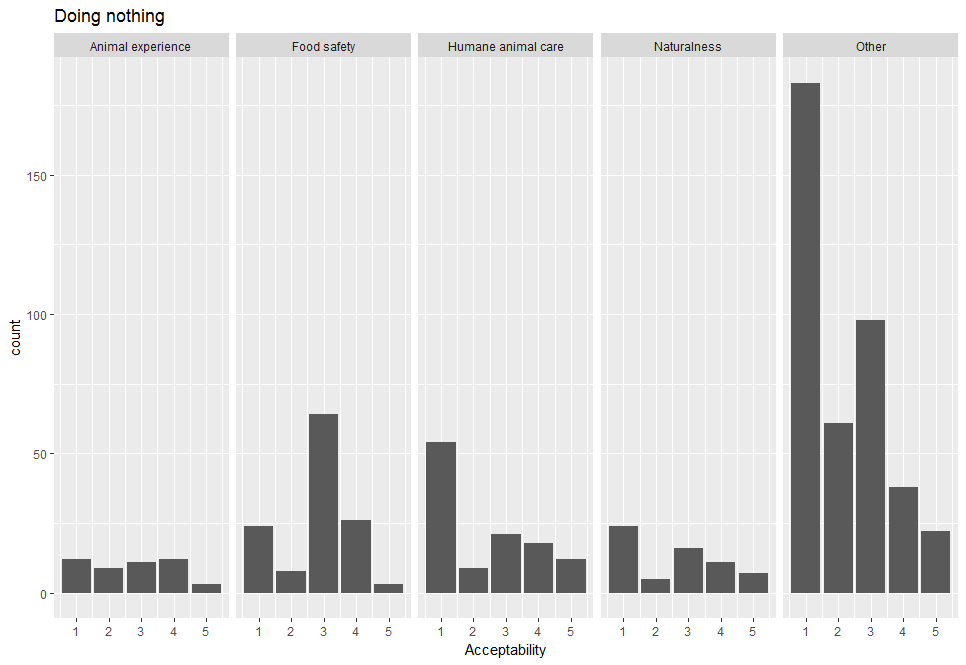 |

## E: Exploratory factor analysis data tables

## Broilers

***Table O. Summary of factor scores for the broiler survey per country, created through an explanatory factor analysis. The numbers indicate the mean response on a linear scale 1 (Strongly agree) to 5 (Strongly disagree) ± SD).***

|  | Finland n=158 | | German n=158 | | Poland n=157 | | Spain n=158 | | UK n=158 | | Total n=789 | |
| --- | --- | --- | --- | --- | --- | --- | --- | --- | --- | --- | --- | --- |
|  | Mean | SD | Mean | SD | Mean | SD | Mean | SD | Mean | SD | Mean | SD |
| Non-benefits | 2.72 | 0.84 | 2.62 | 1.12 | 2.70 | 0.91 | 3.46 | 1.15 | 2.67 | 0.93 | 2.84 | 1.04 |
| Concern | 3.90 | 0.86 | 4.13 | 0.78 | 3.94 | 0.73 | 4.01 | 0.83 | 3.95 | 0.78 | 3.99 | 0.80 |
| Proactive | 4.19 | 0.72 | 4.09 | 0.76 | 3.98 | 0.79 | 4.06 | 0.67 | 4.04 | 0.71 | 4.07 | 0.73 |
| Distrust | 2.83 | 0.82 | 3.01 | 0.97 | 2.88 | 0.88 | 3.30 | 0.86 | 3.23 | 0.84 | 3.05 | 0.89 |
| Personal risk | 3.06 | 0.90 | 3.50 | 1.00 | 3.37 | 0.90 | 3.36 | 1.03 | 3.36 | 0.92 | 3.33 | 0.96 |
| Perceived benefit | 3.06 | 1.00 | 2.75 | 1.10 | 2.81 | 0.81 | 3.14 | 0.87 | 2.79 | 1.01 | 2.91 | 0.97 |
| Pro-consumption | 2.59 | 0.92 | 2.13 | 0.87 | 2.45 | 0.74 | 2.67 | 0.73 | 2.61 | 0.83 | 2.49 | 0.84 |
| Attitude | 3.09 | 0.73 | 3.03 | 0.92 | 3.04 | 0.82 | 3.47 | 0.90 | 3.41 | 0.78 | 3.21 | 0.85 |
| Treatment | 3.09 | 0.97 | 3.79 | 0.86 | 3.38 | 0.80 | 3.71 | 0.96 | 3.55 | 0.92 | 3.50 | 0.94 |
| Medicine consumption | 3.58 | 0.91 | 3.43 | 0.90 | 3.45 | 0.93 | 3.54 | 0.93 | 3.61 | 0.78 | 3.52 | 0.89 |
| Governance | 3.75 | 0.98 | 3.71 | 1.07 | 3.39 | 0.99 | 3.98 | 0.97 | 3.87 | 0.93 | 3.74 | 1.01 |
| Anti-consumption | 3.41 | 0.95 | 3.62 | 0.87 | 3.55 | 0.77 | 3.52 | 0.77 | 3.60 | 0.80 | 3.54 | 0.84 |
| Perceived risks | 3.42 | 0.85 | 3.84 | 0.89 | 3.68 | 0.75 | 3.56 | 1.00 | 3.69 | 0.79 | 3.64 | 0.87 |
| Non-risks | 2.56 | 0.78 | 2.68 | 0.88 | 2.87 | 0.93 | 2.97 | 1.05 | 2.76 | 0.86 | 2.77 | 0.91 |
| Feed and light | 3.58 | 0.76 | 3.52 | 0.88 | 3.38 | 0.90 | 3.72 | 0.84 | 3.53 | 0.86 | 3.55 | 0.85 |
| Media | 3.04 | 0.77 | 3.32 | 0.93 | 3.12 | 0.90 | 3.30 | 0.93 | 3.07 | 0.85 | 3.17 | 0.88 |

***Table P Explanatory factor analysis results for the broiler survey.***

|  | 1 | 2 | 3 |
| --- | --- | --- | --- |
| **Non-risks** |  |  |  |
| Q8_6: Improved human food quality | 0.847 |  |  |
| Q8_2: Reduced incidence of animal diseases | 0.837 |  |  |
| Q8_8: Improved consumer health | 0.836 |  |  |
| Q8_7: Improved nutritional quality of human food | 0.828 |  |  |
| Q8_5: Improved human food safety | 0.823 |  |  |
| Q8_4: Improved animal welfare monitoring | 0.806 |  |  |
| Q8_1: Reduced animal stress | 0.800 |  |  |
| Q8_21: Increased consumer trust in the food they buy | 0.797 |  |  |
| Q8_22: A natural production method | 0.766 |  |  |
| Q8_3: Faster treatment of animal diseases | 0.757 |  |  |
| Q8_12: A more sustainable approach to animal production | 0.708 |  |  |
| Q8_19: Benefits to your family | 0.685 |  |  |
| Q8_11: Benefits to the environment e.g. reduced CO2 footprint | 0.683 |  |  |
| Q8_18: Benefits to you personally | 0.681 |  |  |
| Q8_20: Benefits to consumers | 0.669 |  |  |
| Q8_16: More professionally run livestock farms | 0.645 |  |  |
| Q8_17: Benefits to agriculture | 0.528 |  |  |
| **Concern** |  |  |  |
| Q10_12: Impacts of animal diseases on food quality |  | 0.852 |  |
| Q10_13: Impacts of animal diseases on food safety |  | 0.815 |  |
| Q10_10: Impacts of animal diseases on human health |  | 0.796 |  |
| Q10_16: Animal production diseases in general |  | 0.715 |  |
| Q10_15: Antibiotic resistance as a result of the use of antibiotics in animals |  | 0.711 |  |
| Q10_14: Impact of animal diseases on the environment |  | 0.696 |  |
| Q10_11: Impacts of animal diseases on animal welfare |  | 0.680 |  |
| Q10_9: Antibiotic residues in food |  | 0.674 |  |
| Q10_3: Use of antibiotics in production animals as a growth promoter |  | 0.670 |  |
| Q10_2: Whether animal welfare standards are actually achieved in broiler chicken production |  | 0.495 |  |
| Q10_4: Use of antibiotics in production animals to prevent diseases |  | 0.452 |  |
| **Proactive** |  |  |  |
| Q13_12: Improvements in housing design |  |  | 0.808 |
| Q13_11: Providing materials and an environment where birds can perform natural behaviours |  |  | 0.780 |
| Q13_14: Reducing the number of birds in a given area |  |  | 0.748 |
| Q13_13: Housing that protects the birds from adverse natural conditions |  |  | 0.720 |
| Q13_6: Enhanced hygiene and disease prevention measures |  |  | 0.719 |
| Q13_5: Enhanced maintenance of the quality of the bedding |  |  | 0.714 |
| Q13_10: Providing farmers with a price premium that encourages enhanced bird health |  |  | 0.703 |
| Q13_7: Housing that allows birds greater freedom to move |  |  | 0.696 |
| Q13_4: Enhanced control of air movement in chicken houses |  |  | 0.565 |
|  |  |  |  |
| **Cronbach's alpha** | 0.975 | 0.944 | 0.930 |

|  | 4 | 5 | 6 | 7 | 8 | 9 |
| --- | --- | --- | --- | --- | --- | --- |
| **Distrust** |  |  |  |  |  |  |
| Q6_12: Slaughterhouses | -0.865 |  |  |  |  |  |
| Q6_7: Animal breeding companies | -0.854 |  |  |  |  |  |
| Q6_8: Animal feed producers | -0.815 |  |  |  |  |  |
| Q6_14: Food manufacturers | -0.804 |  |  |  |  |  |
| Q6_11: Animal transporters | -0.796 |  |  |  |  |  |
| Q6_10: Farmers | -0.790 |  |  |  |  |  |
| Q6_15: Food retailers | -0.717 |  |  |  |  |  |
| Q6_9: Animal housing manufacturers | -0.683 |  |  |  |  |  |
| Q6_6: Veterinary medicine producers | -0.591 |  |  |  |  |  |
| Q6_3: National government | -0.534 |  |  |  |  |  |
| **Personal risks** |  |  |  |  |  |  |
| Q9_19: Risks to your family |  | 0.757 |  |  |  |  |
| Q9_18: Risks to you personally |  | 0.735 |  |  |  |  |
| Q9_20: Risks to consumers |  | 0.732 |  |  |  |  |
| Q9_17: Risks to agriculture |  | 0.405 |  |  |  |  |
| **Perceived benefit** |  |  |  |  |  |  |
| Q8_13: A more cost efficient production method |  |  | 0.727 |  |  |  |
| Q8_9: Cheaper food of animal origin |  |  | 0.723 |  |  |  |
| Q8_10: Increased availability of animal based products |  |  | 0.700 |  |  |  |
| Q8_14: Greater protection from predators |  |  | 0.636 |  |  |  |
| Q8_15: Greater protection from bad weather |  |  | 0.633 |  |  |  |
| **Pro-consumption** |  |  |  |  |  |  |
| Q16_2: I intend to purchase foods produced using intensive production systems |  |  |  | 0.871 |  |  |
| Q16_1: I purchase foods produced using intensive production systems |  |  |  | 0.831 |  |  |
| Q16_3: I would consider purchasing foods produced by intensive production systems |  |  |  | 0.769 |  |  |
| **Attitude** |  |  |  |  |  |  |
| Q7_1: unpleasant/ pleasant |  |  |  |  | 0.750 |  |
| Q7_2: bad/ good |  |  |  |  | 0.710 |  |
| Q7_6: unethical/ ethical |  |  |  |  | 0.670 |  |
| Q7_5: unsafe/ safe |  |  |  |  | 0.603 |  |
| Q7_3: worthless/ valuable |  |  |  |  | 0.599 |  |
| Q7_4: useless/ useful |  |  |  |  | 0.520 |  |
| **Treatment** |  |  |  |  |  |  |
| Q13_15: Using antibiotics and medicines to treat sick birds |  |  |  |  |  | -0.653 |
| Q13_8: The preventative use of veterinary drugs including antibiotics |  |  |  |  |  | -0.582 |
| Q13_16: The use of vaccination |  |  |  |  |  | -0.554 |
|  |  |  |  |  |  |  |
| **Cronbach's alpha** | 0.925 | 0.927 | 0.865 | 0.897 | 0.880 | 0.708 |

|  | 10 | 11 | 12 | 13 | 14 | 15 | 16 |
| --- | --- | --- | --- | --- | --- | --- | --- |
| **Medicine concerns** |  |  |  |  |  |  |  |
| Q10_7: Use of other veterinary medicines to treat animal diseases | 0.798 |  |  |  |  |  |  |
| Q10_6: Use of vaccinations to prevent animal diseases | 0.773 |  |  |  |  |  |  |
| Q10_8: Use of probiotics to prevent animal diseases | 0.639 |  |  |  |  |  |  |
| Q10_5: Use of antibiotics in production animals to treat diseases | 0.624 |  |  |  |  |  |  |
| **Governance** |  |  |  |  |  |  |  |
| Q6_5: Animal health authorities |  | -0.435 |  |  |  |  |  |
| **Anti-consumption** |  |  |  |  |  |  |  |
| Q16_8: I think of myself as someone who is concerned about intensive systems |  |  | 0.736 |  |  |  |  |
| Q16_9: I think of myself as someone who is concerned about FAW |  |  | 0.729 |  |  |  |  |
| Q16_7: I feel that I have an ethical obligation to avoid animal products systems |  |  | 0.703 |  |  |  |  |
| Q16_5: I avoid purchasing foods from intensive production systems |  |  | 0.566 |  |  |  |  |
| Q16_4: I plan to reduce my consumption of foods from intensive systems |  |  | 0.534 |  |  |  |  |
| **Perceived risk** |  |  |  |  |  |  |  |
| Q9_2: Increased incidence of animal diseases |  |  |  | 0.770 |  |  |  |
| Q9_5: Reduced human food safety |  |  |  | 0.666 |  |  |  |
| Q9_7: Reduced nutritional quality of human food |  |  |  | 0.657 |  |  |  |
| Q9_3: Slower treatment of animal diseases |  |  |  | 0.652 |  |  |  |
| Q9_6: Reduced human food quality |  |  |  | 0.649 |  |  |  |
| Q9_4: Compromised animal welfare monitoring |  |  |  | 0.637 |  |  |  |
| Q9_8: Negative effects on consumer health |  |  |  | 0.625 |  |  |  |
| Q9_1: Increased animal stress |  |  |  | 0.594 |  |  |  |
| **Non-benefits** |  |  |  |  |  |  |  |
| Q9_15: Less protection from bad weather |  |  |  |  | 0.602 |  |  |
| Q9_14: Less protection from predators |  |  |  |  | 0.599 |  |  |
| Q9_13: A non-cost efficient production method |  |  |  |  | 0.559 |  |  |
| Q9_10: Decreased availability of animal based foods |  |  |  |  | 0.497 |  |  |
| Q9_9: More expensive food of animal origin |  |  |  |  | 0.441 |  |  |
| **Feed and light** |  |  |  |  |  |  |  |
| Q13_2: Adjustments to feed composition |  |  |  |  |  | -0.760 |  |
| Q13_1: Adjustments in the quantity of feed available |  |  |  |  |  | -0.708 |  |
| Q13_3: Changes in the amount and time of light provision |  |  |  |  |  | -0.682 |  |
| **Media** |  |  |  |  |  |  |  |
| Q6_19: Social media |  |  |  |  |  |  | 0.533 |
| Q6_20: Traditional media |  |  |  |  |  |  | 0.516 |
|  |  |  |  |  |  |  |  |
| **Cronbach's alpha** | 0.854 | * | 0.864 | 0.937 | 0.883 | 0.820 | 0.622 |

Sample size n=789 EFA was conducted using principle factor extraction and a direct-oblimin rotation. A KMO of 0.945 and Bartlett’s test of sphericity led to the rejection of the null hypothesis (χ^2^(5995)=75035.232, *p=0.000*, indicating that there was sufficient correlation in the data to proceed with factor analysis.

*Cronbach’s alpha was not calculated for scales with only 1 item

## Layers

***Table Q Summary of factor scores for the layers survey per country, created through an explanatory factor analysis. The numbers indicate the mean response on a linear scale 1 (Strongly agree) to 5 (Strongly disagree) ± SD).***

|  | Finland n=158 | | Germany n=160 | | Poland n=156 | | Spain n=158 | | UK n=158 | | Total n=790 | |
| --- | --- | --- | --- | --- | --- | --- | --- | --- | --- | --- | --- | --- |
|  | Mean | SD | Mean | SD | Mean | SD | Mean | SD | Mean | SD | Mean | SD |
| Non-benefits | 2.77 | 0.88 | 2.48 | 1.03 | 2.84 | 0.88 | 3.41 | 1.04 | 2.70 | 1.01 | 2.84 | 1.02 |
| Concern | 3.91 | 0.79 | 4.12 | 0.78 | 3.98 | 0.84 | 3.92 | 0.84 | 3.94 | 0.70 | 3.97 | 0.79 |
| Non-risks | 2.64 | 0.82 | 2.69 | 0.96 | 2.84 | 0.90 | 3.10 | 0.98 | 2.87 | 0.93 | 2.83 | 0.93 |
| Distrust | 3.70 | 0.69 | 3.90 | 0.71 | 3.76 | 0.68 | 3.84 | 0.76 | 3.79 | 0.64 | 3.80 | 0.70 |
| Medicine concerns | 3.26 | 0.86 | 3.91 | 0.86 | 3.62 | 0.89 | 3.70 | 0.94 | 3.65 | 0.89 | 3.63 | 0.91 |
| Perceived benefit | 3.58 | 0.86 | 3.41 | 0.95 | 3.41 | 0.80 | 3.58 | 0.85 | 3.55 | 0.87 | 3.50 | 0.87 |
| Anti-consumption | 3.33 | 0.88 | 3.65 | 0.93 | 3.51 | 0.76 | 3.45 | 0.69 | 3.62 | 0.79 | 3.51 | 0.82 |
| Personal risk | 3.15 | 0.87 | 3.65 | 0.92 | 3.41 | 0.84 | 3.53 | 0.92 | 3.41 | 0.91 | 3.43 | 0.90 |
| Attitude | 2.74 | 0.76 | 2.17 | 0.82 | 2.65 | 0.74 | 2.60 | 0.74 | 2.61 | 0.90 | 2.55 | 0.82 |
| Pro-consumption | 3.00 | 0.95 | 2.77 | 1.07 | 2.91 | 0.87 | 3.11 | 0.95 | 2.78 | 1.13 | 2.91 | 1.00 |
| Perceived risk | 3.41 | 0.85 | 3.90 | 0.85 | 3.54 | 0.84 | 3.70 | 0.83 | 3.70 | 0.88 | 3.65 | 0.86 |
| Feed and light | 3.55 | 0.82 | 3.55 | 0.84 | 3.39 | 0.79 | 3.73 | 0.77 | 3.62 | 0.79 | 3.57 | 0.81 |
| Treatment | 3.07 | 0.72 | 3.01 | 0.84 | 3.49 | 0.66 | 3.45 | 0.88 | 3.43 | 0.79 | 3.29 | 0.81 |
| Media | 3.22 | 0.71 | 3.54 | 0.86 | 3.37 | 0.74 | 3.45 | 0.77 | 3.26 | 0.78 | 3.37 | 0.78 |
| Proactive | 4.21 | 0.69 | 4.02 | 0.81 | 3.99 | 0.74 | 4.00 | 0.74 | 4.07 | 0.68 | 4.06 | 0.74 |

Two less factors are presented than obtained during the exploratory factor analysis, as two factors contained variables that did not load sufficiently (i.e. less than the cut-off of 0.4), therefore could not have mean values calculated.

***Table R Explanatory factor analysis results for the layers survey.***

|  | 1 | 2 | 3 | 4 |
| --- | --- | --- | --- | --- |
| **Non-benefits** |  |  |  |  |
| Q8_6: Improved human food quality | 0.835 |  |  |  |
| Q8_1: Reduced animal stress | 0.832 |  |  |  |
| Q8_7: Improved nutritional quality of human food | 0.814 |  |  |  |
| Q8_8: Improved consumer health | 0.810 |  |  |  |
| Q8_2: Reduced incidence of animal diseases | 0.795 |  |  |  |
| Q8_21: Increased consumer trust in the food they buy | 0.791 |  |  |  |
| Q8_5: Improved human food safety | 0.788 |  |  |  |
| Q8_22: A natural production method | 0.774 |  |  |  |
| Q8_4: Improved animal welfare monitoring | 0.771 |  |  |  |
| Q8_3: Faster treatment of animal diseases | 0.696 |  |  |  |
| Q8_19: Benefits to your family | 0.688 |  |  |  |
| Q8_12: A more sustainable approach to animal production | 0.680 |  |  |  |
| Q8_18: Benefits to you personally | 0.669 |  |  |  |
| Q8_20: Benefits to consumers | 0.618 |  |  |  |
| Q8_11: Benefits to the environment | 0.615 |  |  |  |
| Q8_16: More professionally run livestock farms | 0.491 |  |  |  |
| Q8_17: Benefits to agriculture | 0.415 |  |  |  |
| **Concern** |  |  |  |  |
| Q10_13: Impacts of animal diseases on food safety |  | 0.855 |  |  |
| Q10_12: Impacts of animal diseases on food quality |  | 0.835 |  |  |
| Q10_10: Impacts of animal diseases on human health |  | 0.815 |  |  |
| Q10_11: Impacts of animal diseases on animal welfare |  | 0.756 |  |  |
| Q10_16: Animal production diseases in general |  | 0.640 |  |  |
| Q10_14: Impact of animal diseases on the environment |  | 0.601 |  |  |
| Q10_9: Antibiotic residues in food |  | 0.564 |  |  |
| Q10_15: AMR as a result of the use of antibiotics in animals |  | 0.537 |  |  |
| Q10_3: Use of antibiotics in animals as a growth promoter |  | 0.491 |  |  |
| **Non-risks** |  |  |  |  |
| Q9_9: More expensive food of animal origin |  |  | -0.669 |  |
| Q9_15: Less protection from bad weather |  |  | -0.654 |  |
| Q9_10: Decreased availability of animal based foods |  |  | -0.647 |  |
| Q9_14: Less protection from predators |  |  | -0.583 |  |
| Q9_13: A non-cost efficient production method |  |  | -0.534 |  |
| **Distrust** |  |  |  |  |
| Q6_8: Animal feed manufacturers |  |  |  | -0.841 |
| Q6_10: Farmers |  |  |  | -0.817 |
| Q6_11: Animal transporters |  |  |  | -0.808 |
| Q6_14: Food manufacturers |  |  |  | -0.805 |
| Q6_15: Food retailers |  |  |  | -0.796 |
| Q6_7: Animal breeding companies |  |  |  | -0.757 |
| Q6_12: Slaughterhouses |  |  |  | -0.736 |
| Q6_9: Animal housing manufacturers |  |  |  | -0.698 |
| Q6_6: Veterinary medicine producers |  |  |  | -0.588 |
| Q6_13: Quality assurance schemes |  |  |  | -0.470 |
| Q6_3: National government |  |  |  | -0.410 |
|  |  |  |  |  |
| **Cronbach's alpha** | 0.931 | 0.936 | 0.889 | 0.944 |

|  | 5 | 6 | 7 | 8 | 9 | 10 | 11 |  |
| --- | --- | --- | --- | --- | --- | --- | --- | --- |
| **Medicine concerns** |  |  |  |  |  |  |  |  |
| Q10_6: Use of vaccinations to prevent animal diseases | -0.862 |  |  |  |  |  |  |  |
| Q10_7: Use of other veterinary medicines to treat animal diseases | -0.815 |  |  |  |  |  |  |  |
| Q10_8: Use of probiotics to prevent animal diseases | -0.692 |  |  |  |  |  |  |  |
| Q10_5: Use of antibiotics in production animals to treat diseases | -0.684 |  |  |  |  |  |  |  |
| Q10_4: Use of antibiotics in production animals to prevent diseases | -0.485 |  |  |  |  |  |  |  |
| **Perceived benefit** |  |  |  |  |  |  |  |  |
| Q8_13: A more cost efficient production method |  | 0.723 |  |  |  |  |  |  |
| Q8_10: Increased availability of animal based products |  | 0.650 |  |  |  |  |  |  |
| Q8_14: Greater protection from predators |  | 0.650 |  |  |  |  |  |  |
| Q8_9: Cheaper food of animal origin |  | 0.646 |  |  |  |  |  |  |
| Q8_15: Greater protection from bad weather |  | 0.628 |  |  |  |  |  |  |
| **Anti-consumption** |  |  |  |  |  |  |  |  |
| Q16_8: I think of myself as someone who is concerned about intensive production systems |  |  | -0.732 |  |  |  |  |  |
| Q16_4: I plan to reduce my consumption of foods from intensive production systems |  |  | -0.653 |  |  |  |  |  |
| Q16_5: I avoid purchasing foods from intensive production systems |  |  | -0.648 |  |  |  |  |  |
| Q16_9: I think of myself as someone who is concerned about farm animal welfare |  |  | -0.639 |  |  |  |  |  |
| Q16_7: I feel that I have an ethical obligation to avoid products from intensive systems |  |  | -0.576 |  |  |  |  |  |
| **Personal risk** |  |  |  |  |  |  |  |  |
| Q9_20: Risks to consumers |  |  |  |  | -0.886 |  |  |  |
| Q9_19: Risks to your family |  |  |  |  | -0.876 |  |  |  |
| Q9_18: Risks to you personally |  |  |  |  | -0.843 |  |  |  |
| Q9_21: Decreased consumer trust in the food they buy |  |  |  |  | -0.704 |  |  |  |
| Q9_17: Risks to agriculture |  |  |  |  | -0.541 |  |  |  |
| Q9_8: Negative effects on consumer health |  |  |  |  | -0.461 |  |  |  |
| Q9_22: An unnatural production method |  |  |  |  | -0.447 |  |  |  |
| **Attitude** |  |  |  |  |  |  |  |  |
| Q7_3: worthless/ valuable |  |  |  |  |  | 0.686 |  |  |
| Q7_2: bad/ good |  |  |  |  |  | 0.647 |  |  |
| Q7_1: unpleasant/ pleasant |  |  |  |  |  | 0.640 |  |  |
| Q7_6: unethical/ ethical |  |  |  |  |  | 0.582 |  |  |
| Q7_5: unsafe/ safe |  |  |  |  |  | 0.534 |  |  |
| Q7_4: useless/ useful |  |  |  |  |  | 0.532 |  |  |
| **Pro-consumption** |  |  |  |  |  |  |  |  |
| Q16_2: I intend to purchase foods produced using IAP |  |  |  |  |  |  | -0.780 |  |
| Q16_1: I purchase foods produced using IAP |  |  |  |  |  |  | -0.755 |  |
| Q16_3: I would consider purchasing foods produced by IAP |  |  |  |  |  |  | -0.754 |  |
|  |  |  |  |  |  |  |  |  |
| **Cronbach's alpha** | 0.882 | 0.864 | 0.854 | * | 0.933 | 0.867 | 0.903 | |

|  | 12 | 13 | 14 | 15 | 16 | 17 |
| --- | --- | --- | --- | --- | --- | --- |
| **Perceived risk** |  |  |  |  |  |  |
| Q9_4: Compromised animal welfare monitoring | 0.606 |  |  |  |  |  |
| Q9_2: Increased incidence of animal diseases | 0.602 |  |  |  |  |  |
| Q9_3: Slower treatment of animal diseases | 0.572 |  |  |  |  |  |
| Q9_5: Reduced human food safety | 0.499 |  |  |  |  |  |
| Q9_1: Increased animal stress | 0.452 |  |  |  |  |  |
| Q9_6: Reduced human food quality | 0.421 |  |  |  |  |  |
| **Feed and light** |  |  |  |  |  |  |
| Q13_1: Adjustments to the quantity of feed available |  | 0.775 |  |  |  |  |
| Q13_2: Adjustments to feed composition |  | 0.703 |  |  |  |  |
| Q13_3: Changes in the amount and time of light |  | 0.557 |  |  |  |  |
| **Treatment** |  |  |  |  |  |  |
| Q13_16: The use of vaccination |  |  | 0.621 |  |  |  |
| Q13_15: Using antibiotics to treat sick birds |  |  | 0.589 |  |  |  |
| Q13_8: The preventative use of veterinary drugs |  |  | 0.526 |  |  |  |
| Q13_9: Use of feed supplements e.g. probiotics |  |  | 0.525 |  |  |  |
| **Media** |  |  |  |  |  |  |
| Q6_20: Traditional media |  |  |  | 0.577 |  |  |
| Q6_19: Social media |  |  |  | 0.546 |  |  |
| Q6_16: Consumer organisations |  |  |  | 0.484 |  |  |
| **Proactive** |  |  |  |  |  |  |
| Q13_11: Providing materials and an environment where birds can perform natural behaviours |  |  |  |  | -0.838 |  |
| Q13_12: Improvements in housing design |  |  |  |  | -0.783 |  |
| Q13_6: Enhanced hygiene and disease prevention |  |  |  |  | -0.773 |  |
| Q13_14: Reducing the number of birds in a given area |  |  |  |  | -0.761 |  |
| Q13_5: Enhanced maintenance of the bedding |  |  |  |  | -0.699 |  |
| Q13_7: Housing that allows birds greater freedom to move |  |  |  |  | -0.686 |  |
| Q13_13: Housing that protects the birds from adverse natural conditions |  |  |  |  | -0.650 |  |
| Q13_10: Providing farmers with a price premium that encourages enhanced bird health |  |  |  |  | -0.542 |  |
| Q13_4: Enhanced control of air movement |  |  |  |  | -0.521 |  |
|  |  |  |  |  |  |  |
| **Cronbach's alpha** | 0.906 | 0.775 | 0.733 | 0.668 | 0.929 | * |

Sample size is n=790. EFA was conducted using principle factor extraction and a direct-oblimin rotation. A KMO of 0.947 and Bartlett’s test of sphericity led to the rejection of the null hypothesis (χ^2^(5995)=74906.485, *p=0.000*, indicating that there was sufficient correlation in the data to proceed with factor analysis.

*These factors had no items with factor loadings greater than 0.4 therefore Cronbach’s alpha values were not calculated

IPA stands for intensive animal production

## Pigs

***Table S Summary of factor scores for the pigs survey per country, created through an explanatory factor analysis. The numbers indicate the mean response on a linear scale 1 (Strongly agree) to 5 (Strongly disagree) ± SD).***

|  | Finland n=150 | | Germany n=150 | | Poland n=151 | | Spain n=152 | | UK n=148 | | Total n=751 | |
| --- | --- | --- | --- | --- | --- | --- | --- | --- | --- | --- | --- | --- |
|  | Mean | SD | Mean | SD | Mean | SD | Mean | SD | Mean | SD | Mean | SD |
| Non-benefits | 2.79 | 0.84 | 2.49 | 1.13 | 2.78 | 0.88 | 3.59 | 1.10 | 2.84 | 0.93 | 2.90 | 1.05 |
| Concern | 3.88 | 0.83 | 4.26 | 0.76 | 3.95 | 0.79 | 3.95 | 0.80 | 3.86 | 0.83 | 3.98 | 0.81 |
| Proactive | 4.18 | 0.60 | 4.10 | 0.76 | 3.96 | 0.72 | 4.15 | 0.75 | 4.08 | 0.67 | 4.10 | 0.71 |
| Distrust | 2.91 | 0.68 | 2.89 | 1.08 | 3.13 | 0.82 | 3.32 | 0.85 | 3.24 | 0.77 | 3.10 | 0.87 |
| Perceived risk | 3.19 | 0.77 | 3.80 | 0.83 | 3.47 | 0.77 | 3.30 | 1.02 | 3.44 | 0.70 | 3.44 | 0.85 |
| Perceived benefit | 3.53 | 0.77 | 3.33 | 0.96 | 3.49 | 0.77 | 3.77 | 0.81 | 3.57 | 0.82 | 3.54 | 0.84 |
| Pro-consumption | 2.92 | 0.94 | 2.80 | 1.04 | 2.92 | 0.83 | 3.05 | 0.89 | 2.88 | 1.04 | 2.91 | 0.95 |
| Anti-consumption | 3.38 | 0.90 | 3.80 | 0.76 | 3.40 | 0.74 | 3.36 | 0.79 | 3.54 | 0.85 | 3.50 | 0.83 |
| Medicine concerns | 3.18 | 0.94 | 3.90 | 0.89 | 3.46 | 0.85 | 3.72 | 0.91 | 3.45 | 0.87 | 3.54 | 0.92 |
| Independent assurance | 3.31 | 0.51 | 3.51 | 0.79 | 3.50 | 0.73 | 3.58 | 0.73 | 3.42 | 0.65 | 3.46 | 0.69 |
| Treatment | 3.02 | 0.76 | 2.82 | 0.88 | 3.07 | 0.69 | 3.52 | 0.80 | 3.57 | 0.64 | 3.20 | 0.81 |
| Attitude | 2.57 | 0.85 | 2.18 | 0.92 | 2.65 | 0.73 | 2.69 | 0.75 | 2.71 | 0.81 | 2.56 | 0.84 |
| Non-risks | 2.63 | 0.72 | 2.81 | 0.98 | 2.86 | 0.92 | 2.79 | 0.97 | 2.84 | 0.92 | 2.79 | 0.91 |

Two less factors are presented than obtained during the exploratory factor analysis, as two factors contained variables that did not load sufficiently (i.e. less than the cut-off of 0.4), therefore could not have mean values calculated.

|  | 1 | 2 | 3 |
| --- | --- | --- | --- |
| **Non-benefits** |  |  |  |
| Q8_6: Improved human food quality | 0.874 |  |  |
| Q8_7: Improved nutritional quality of human food | 0.856 |  |  |
| Q8_8: Improved consumer health | 0.835 |  |  |
| Q8_1: Reduced animal stress | 0.814 |  |  |
| Q8_5: Improved human food safety | 0.799 |  |  |
| Q8_21: Increased consumer trust in the food they buy | 0.784 |  |  |
| Q8_22: A natural production method | 0.781 |  |  |
| Q8_4: Improved animal welfare monitoring | 0.760 |  |  |
| Q8_2: Reduced incidence of animal diseases | 0.753 |  |  |
| Q8_12: A more sustainable approach to animal production | 0.725 |  |  |
| Q8_3 Faster treatment of animal diseases | 0.701 |  |  |
| Q8_19: Benefits to your family | 0.685 |  |  |
| Q8_18: Benefits to you personally | 0.668 |  |  |
| Q8_20: Benefits to consumers | 0.655 |  |  |
| Q8_16: More professionally run livestock farms | 0.652 |  |  |
| Q8_11: Benefits to the environment e.g. reduced CO2 footprint | 0.624 |  |  |
| Q8_17: Benefits to agriculture | 0.516 |  |  |
| **Concern** |  |  |  |
| Q10_13: Impacts of animal diseases on food safety |  | 0.909 |  |
| Q10_12: Impacts of animal diseases on food quality |  | 0.875 |  |
| Q10_10: Impacts of animal diseases on human health |  | 0.844 |  |
| Q10_16: Animal production diseases in general |  | 0.702 |  |
| Q10_15: Antibiotic resistance as a result of the use of antibiotics in animals |  | 0.695 |  |
| Q10_9: Antibiotic residues in food |  | 0.689 |  |
| Q10_11: Impacts of animal diseases on animal welfare |  | 0.677 |  |
| Q10_3: Use of antibiotics in production animals as a growth promoter |  | 0.633 |  |
| Q10_14: Impact of animal diseases on the environment |  | 0.630 |  |
| **Proactive** |  |  |  |
| Q13_8: Improvements in pigs' diet composition |  |  | 0.900 |
| Q13_7: Enhanced control of air movement in pig houses |  |  | 0.886 |
| Q13_11: Improvements in housing design |  |  | 0.805 |
| Q13_6: Efficient monitoring of pigs and pig housing conditions |  |  | 0.801 |
| Q13_14: Providing enrichment materials so pigs can perform natural behaviours |  |  | 0.767 |
| Q13_13: Reducing the number of pigs in a given area |  |  | 0.730 |
| Q13_12: Housing that protects pigs from adverse natural conditions |  |  | 0.646 |
| Q13_9: Adjustments in the quantity of pig feed available |  |  | 0.643 |
| Q13_15: Providing farmers with a price premium that encourages enhanced animal health |  |  | 0.623 |
| Q13_1: Enhanced hygiene and disease prevention measures |  |  | 0.585 |
|  |  |  |  |
| **Cronbach's alpha** | 0.975 | 0.944 | 0.930 |

***Table T Explanatory factor analysis results for the pigs survey.***

|  | 4 | 5 | 6 | 7 | 8 |
| --- | --- | --- | --- | --- | --- |
| **Distrust** |  |  |  |  |  |
| Q6_7: Animal breeding companies | 0.868 |  |  |  |  |
| Q6_8: Animal feed producers | 0.812 |  |  |  |  |
| Q6_12: Slaughterhouses | 0.797 |  |  |  |  |
| Q6_14: Food manufacturers | 0.791 |  |  |  |  |
| Q6_10: Farmers | 0.784 |  |  |  |  |
| Q6_11: Animal transporters | 0.729 |  |  |  |  |
| Q6_9: Animal housing manufacturers | 0.696 |  |  |  |  |
| Q6_15: Food retailers | 0.655 |  |  |  |  |
| Q6_6: Veterinary medicine producers | 0.639 |  |  |  |  |
| **Perceived risk** |  |  |  |  |  |
| Q9_6: Reduced human food quality |  | 0.781 |  |  |  |
| Q9_20: Risks to consumers |  | 0.777 |  |  |  |
| Q9_19: Risks to your family |  | 0.768 |  |  |  |
| Q9_18: Risks to you personally |  | 0.754 |  |  |  |
| Q9_7: Reduced nutritional quality of human food |  | 0.742 |  |  |  |
| Q9_21: Decreased consumer trust in the food they buy |  | 0.735 |  |  |  |
| Q9_8: Negative effects on consumer health |  | 0.735 |  |  |  |
| Q9_5: Reduced human food safety |  | 0.709 |  |  |  |
| Q9_2: Increased incidence of animal diseases |  | 0.600 |  |  |  |
| Q9_11: Risks to the environment |  | 0.598 |  |  |  |
| Q9_12: An unsustainable approach to animal production |  | 0.594 |  |  |  |
| Q9_17: Risks to agriculture |  | 0.593 |  |  |  |
| Q9_4: Compromised animal welfare monitoring |  | 0.585 |  |  |  |
| Q9_1: Increased animal stress |  | 0.575 |  |  |  |
| Q9_3: Slower treatment of animal diseases |  | 0.560 |  |  |  |
| Q9_22: An unnatural production method |  | 0.553 |  |  |  |
| Q9_16: Less professionally run livestock farms |  | 0.430 |  |  |  |
| **Perceived benefit** |  |  |  |  |  |
| Q8_9: Cheaper food of animal origin |  |  | 0.672 |  |  |
| Q8_13: A more cost efficient production method |  |  | 0.595 |  |  |
| Q8_10: Increased availability of animal based products |  |  | 0.572 |  |  |
| Q8_14: Greater protection from predators |  |  | 0.546 |  |  |
| Q8_15: Greater protection from bad weather |  |  | 0.516 |  |  |
| **Pro-consumption** |  |  |  |  |  |
| Q16_2: I intend to purchase foods produced using IAP |  |  |  | 0.804 |  |
| Q16_1: I purchase foods produced using IAP |  |  |  | 0.785 |  |
| Q16_3: I would consider purchasing foods produced by IAP |  |  |  | 0.743 |  |
| **Anti-consumption** |  |  |  |  |  |
| Q16_8: I think of myself as someone who is concerned about IAP |  |  |  |  | 0.789 |
| Q16_9: I think of myself as someone who is concerned about FAW |  |  |  |  | 0.691 |
| Q16_7: I feel that I have an ethical obligation to avoid animal products from IAP |  |  |  |  | 0.633 |
| Q16_5: I avoid purchasing foods from IAP |  |  |  |  | 0.595 |
| Q16_4: I plan to reduce my consumption of foods from IAP |  |  |  |  | 0.546 |
|  |  |  |  |  |  |
| **Cronbach's alpha** | 0.935 | 0.962 | 0.846 | 0.894 | 0.844 |

|  | 9 | 10 | 11 | 12 | 13 | 14 | 15 |
| --- | --- | --- | --- | --- | --- | --- | --- |
| **Medicine concerns** |  |  |  |  |  |  |  |
| Q10_7: Use of other veterinary medicines to treat animal diseases | -0.827 |  |  |  |  |  |  |
| Q10_6: Use of vaccinations to prevent animal diseases | -0.799 |  |  |  |  |  |  |
| Q10_5: Use of antibiotics in production animals to treat diseases | -0.691 |  |  |  |  |  |  |
| Q10_8: Use of probiotics to prevent animal diseases | -0.687 |  |  |  |  |  |  |
| Q10_4: Use of antibiotics in production animals to prevent diseases | -0.471 |  |  |  |  |  |  |
| **Independent assurance** |  |  |  |  |  |  |  |
| Q6_20: Traditional media |  | -0.669 |  |  |  |  |  |
| Q6_16: Consumer organisations |  | -0.608 |  |  |  |  |  |
| Q6_1: Animal welfare organisations |  | -0.573 |  |  |  |  |  |
| Q6_2: European commission |  | -0.560 |  |  |  |  |  |
| Q6_3: National government |  | -0.471 |  |  |  |  |  |
| Q6_19: Social media |  | -0.434 |  |  |  |  |  |
| Q6_13: Quality assurance schemes |  | -0.428 |  |  |  |  |  |
| Q6_5: Animal health authorities |  | -0.401 |  |  |  |  |  |
| **Treatment** |  |  |  |  |  |  |  |
| Q13_3: The preventive use of veterinary drugs including antibiotics |  |  | -0.675 |  |  |  |  |
| Q13_2: Using medicines and antibiotics to treat sick picks |  |  | -0.638 |  |  |  |  |
| Q13_5: The use of vaccination |  |  | -0.605 |  |  |  |  |
| Q13_4: Use of feed supplements e.g. probiotics |  |  | -0.578 |  |  |  |  |
| **Attitude** |  |  |  |  |  |  |  |
| Q7_2: bad/ good |  |  |  |  |  | 0.649 |  |
| Q7_5: unsafe/ safe |  |  |  |  |  | 0.644 |  |
| Q7_1: unpleasant/pleasant |  |  |  |  |  | 0.622 |  |
| Q7_3: worthless/valuable |  |  |  |  |  | 0.621 |  |
| Q7_6: unethical/ ethical |  |  |  |  |  | 0.619 |  |
| Q7_4: useless/ useful |  |  |  |  |  | 0.455 |  |
| **Non-risks** |  |  |  |  |  |  |  |
| Q9_14: Less protection from predators |  |  |  |  |  |  | -0.666 |
| Q9_15: Less protection from bad weather |  |  |  |  |  |  | -0.660 |
| Q9_10: Decreased availability of animal based foods |  |  |  |  |  |  | -0.613 |
| Q9_13: A non-cost efficient production method |  |  |  |  |  |  | -0.578 |
| Q9_9: More expensive food of animal origin |  |  |  |  |  |  | -0.562 |
|  |  |  |  |  |  |  |  |
| **Cronbach's alpha** | 0.887 | 0.833 | 0.751 | * | * | 0.878 | 0.862 |

Sample size n-751. EFA was conducted using principle factor extraction and a direct-oblimin rotation. A KMO of 0.946 and Bartlett’s test of sphericity led to the rejection of the null hypothesis (χ^2^(5886)=71867.560, *p=0.000*, indicating that there was sufficient correlation in the data to proceed with factor analysis.

*These factors had no items with factor loadings greater than 0.4 therefore Cronbach’s alpha values were not calculated

IAP stands for intensive animal production system

## F: MSEM additional data tables

***Table U Results of the tests for measurement invariance by country for broilers (n=789).***

| Model | χ^2^ | Df | *P* | Δχ^2^ | CFI | ΔCFI | RMSEA |
| --- | --- | --- | --- | --- | --- | --- | --- |
| Model 1 | 4870.9 | 2395 | - | - | 0.883 | - | 0.081 |
| Model 2 | 5073.3 | 2507 | <0.001 | 202.35 | 0.879 | 0.004 | 0.081 |
| Model 3 | 5395.3 | 2619 | <0.001 | 321.97 | 0.869 | 0.010 | 0.082 |
| Model 4 | 5479.8 | 2639 | <0.001 | 84.52 | 0.866 | 0.003 | 0.083 |

***Table V Results of the tests for measurement invariance by country for layers (n=790)***

| Model | χ^2^ | df | *P* | Δχ^2^ | CFI | ΔCFI | RMSEA |
| --- | --- | --- | --- | --- | --- | --- | --- |
| Model 1 | 3219.3 | 1675 | - | - | 0.905 | - | 0.076 |
| Model 2 | 3344.5 | 1767 | 0.0122 | 125.195 | 0.903 | 0.002 | 0.075 |
| Model 3 | 3626.7 | 1859 | <0.001 | 282.228 | 0.892 | 0.012 | 0.078 |
| Model 4 | 3705.8 | 1879 | <0.001 | 79.096 | 0.888 | 0.004 | 0.078 |

***Table W Results of the tests for measurement invariance by country for pigs (n=751)***

| Model | χ^2^ | Df | *P* | Δχ^2^ | CFI | ΔCFI | RMSEA |
| --- | --- | --- | --- | --- | --- | --- | --- |
| Model 1 | 7627.5 | 3605 | - | - | 0.85 | - | 0.086 |
| Model 2 | 7844.5 | 3745 | <0.001 | 217.06 | 0.847 | 0.003 | 0.085 |
| Model 3 | 8323.7 | 3885 | <0.001 | 479.17 | 0.834 | 0.013 | 0.087 |
| Model 4 | 8427.2 | 3905 | <0.001 | 103.51 | 0.831 | 0.003 | 0.088 |

# References

1. Rosati S, Saba A. The perception of risks associated with food‐related hazards and the perceived reliability of sources of information. International journal of food science & technology. 2004;39(5):491-500.

2. Clark B, Stewart GB, Panzone LA, Kyriazakis I, Frewer LJ. A Systematic Review of Public Attitudes, Perceptions and Behaviours Towards Production Diseases Associated with Farm Animal Welfare. Journal of Agricultural and Environmental Ethics. 2016:1-24. doi: 10.1007/s10806-016-9615-x.

3. Clark B, Stewart GB, Panzone LA, Kyriazakis I, Frewer LJ. Citizens, consumers and farm animal welfare: A meta-analysis of willingness-to-pay studies. Food Policy. 2017;68:112-27. doi: <http://dx.doi.org/10.1016/j.foodpol.2017.01.006>.

4. Poínhos R, van der Lans IA, Rankin A, Fischer ARH, Bunting B, Kuznesof S, et al. Psychological determinants of consumer acceptance of personalised nutrition in 9 European countries. PloS one. 2014;9(10):e110614.

5. van Dijk H, Fischer AR, Frewer LJ. Consumer Responses to Integrated Risk-Benefit Information Associated with the Consumption of Food. Risk Analysis. 2011;31(3):429-39. doi: 10.1111/j.1539-6924.2010.01505.x.

6. Fischer ARH, Frewer LJ. Consumer familiarity with foods and the perception of risks and benefits. Food Quality and Preference. 2009;20(8):576-85. doi: <http://dx.doi.org/10.1016/j.foodqual.2009.06.008>.

7. Frewer LJ, Shepherd R, Sparks P. THE INTERRELATIONSHIP BETWEEN PERCEIVED KNOWLEDGE, CONTROL AND RISK ASSOCIATED WITH A RANGE OF FOOD-RELATED HAZARDS TARGETED AT THE INDIVIDUAL, OTHER PEOPLE AND SOCIETY. Journal of Food Safety. 1994;14(1):19-40. doi: 10.1111/j.1745-4565.1994.tb00581.x.

8. Frewer LJ, Howard C, Hedderley D, Shepherd R. What determines trust in information about food‐related risks? Underlying psychological constructs. Risk analysis. 1996;16(4):473-86.

9. Poortinga W, Bickerstaff K, Langford I, Niewöhner J, Pidgeon N. The British 2001 Foot and Mouth crisis: a comparative study of public risk perceptions, trust and beliefs about government policy in two communities. Journal of Risk Research. 2004;7(1):73-90. doi: 10.1080/1366987042000151205.

10. Poortinga W, Pidgeon NF. Exploring the dimensionality of trust in risk regulation. Risk analysis. 2003;23(5):961-72.

11. Poortinga W, Pidgeon NF. Trust in risk regulation: Cause or consequence of the acceptability of GM food? Risk analysis. 2005;25(1):199-209.

12. Crites SL, Fabrigar LR, Petty RE. Measuring the affective and cognitive properties of attitudes: Conceptual and methodological issues. Personality and Social Psychology Bulletin. 1994;20(6):619-34.

13. Ajzen I. From intentions to actions: A theory of planned behavior. Action control: Springer; 1985. p. 11-39.

14. Ajzen I. The theory of planned behavior. Organizational behavior and human decision processes. 1991;50(2):179-211.

15. Ajzen I. The theory of planned behaviour: reactions and reflections. Taylor & Francis; 2011.

16. Melnyk V, Herpen Ev, Fischer ARH, van Trijp H. To think or not to think: the effect of cognitive deliberation on the influence of injunctive versus descriptive social norms. Psychology & Marketing. 2011;28(7):709-29.

17. Latvala T, Niva M, Mäkelä J, Pouta E, Heikkilä J, Kotro J, et al. Diversifying meat consumption patterns: Consumers' self-reported past behaviour and intentions for change. Meat Science. 2012;92(1):71-7.

18. Verbeke W, Viaene J. Beliefs, attitude and behaviour towards fresh meat consumption in Belgium: empirical evidence from a consumer survey. Food Quality and Preference. 1999;10(6):437-45.

19. Jones P, Niemi JK, Tranter R. List of stakeholder preferred interventions. . 2016.

20. Shaw D, Shiu E. The role of ethical obligation and self‐identity in ethical consumer choice. International Journal of Consumer Studies. 2002;26(2):109-16.

21. Sparks P, Shepherd R, Frewer LJ. Assessing and Structuring Attitudes Toward the Use of Gene Technology in Food Production: The Role of Perceived Ethical Obligation. Basic and Applied Social Psychology. 1995;16(3):267-85. doi: 10.1207/s15324834basp1603_1.

22. Bennett R, Blaney R. Social consensus, moral intensity and willingness to pay to address a farm animal welfare issue. Journal of Economic Psychology. 2002;23(4):501-20. doi: 10.1016/s0167-4870(02)00098-3. PubMed PMID: WOS:000176904600005.

23. Bennett RM, Anderson J, Blaney RJP. Moral intensity and willingness to pay concerning farm animal welfare issues and the implications for agricultural policy. Journal of Agricultural and Environmental Ethics. 2002;15(2):187-202.

24. Population data [Internet]. European Commission. Available from: <http://ec.europa.eu/eurostat/web/population-demography-migration-projections/population-data/database>

25. Nations U. Provisional guidelines on standard international age classifications. New York: United Nations, 1982.

26. ESS-ERIC. Appendix A2. Income, ESS6 - 2012 ed. 2.0 2012. Available from: <https://www.europeansocialsurvey.org/docs/round6/survey/ESS6_appendix_a2_e02_0.pdf>.

27. United Nations Educational SaCO. International Standard Classification of Education. Montreal, Canada: UNESCO Institute for Statistics, 2012.

28. Eurostat. Glossary:Degree of urbanisation 2015. Available from: <http://ec.europa.eu/eurostat/statistics-explained/index.php/Glossary:Degree_of_urbanisation>.

29. Kendall HA, Lobao LM, Sharp JS. Public Concern with Animal Well‐Being: Place, Social Structural Location, and Individual Experience*. Rural Sociology. 2006;71(3):399-428.

30. Commission E. Discrimination in the EU in 2012. 2012 Contract No.: Special Eurobarometer 393.
